# SARS-CoV-2 binding and neutralizing antibody levels after Ad26.COV2.S vaccination predict durable protection in rhesus macaques

**DOI:** 10.1038/s41467-021-26117-x

**Published:** 2021-10-07

**Authors:** Ramon Roozendaal, Laura Solforosi, Daniel J. Stieh, Jan Serroyen, Roel Straetemans, Anna Dari, Muriel Boulton, Frank Wegmann, Sietske K. Rosendahl Huber, Joan E. M. van der Lubbe, Jenny Hendriks, Mathieu Le Gars, Liesbeth Dekking, Dominika N. Czapska-Casey, Nuria Guimera, Sarah Janssen, Sarah Tete, Abishek Chandrashekar, Noe B. Mercado, Jingyou Yu, Wouter Koudstaal, Juan J. Perez-Ruixo, Jerry Sadoff, Dan H. Barouch, Hanneke Schuitemaker, Roland Zahn

**Affiliations:** 1 Janssen Vaccines & Prevention B.V., Leiden, The Netherlands; 2 Janssen R&D, Beerse, Belgium; 3 Center for Virology and Vaccine Research, Beth Israel Deaconess Medical Center, Harvard Medical School, Boston, MA USA; 4 Lucidity Biomedical Consulting, Granada, Spain; 5 Ragon Institute of MGH, MIT and Harvard, Cambridge, MA USA; 6 Harvard Medical School, Boston, MA USA; 7 Massachusetts Consortium on Pathogen Readiness, Boston, MA USA

**Keywords:** Viral infection, Vaccines, SARS-CoV-2, Predictive markers

## Abstract

Several COVID-19 vaccines have recently gained authorization for emergency use. Limited knowledge on duration of immunity and efficacy of these vaccines is currently available. Data on other coronaviruses after natural infection suggest that immunity to SARS-CoV-2 might be short-lived, and preliminary evidence indicates waning antibody titers following SARS-CoV-2 infection. In this work, we model the relationship between immunogenicity and protective efficacy of a series of Ad26 vectors encoding stabilized variants of the SARS-CoV-2 Spike protein in rhesus macaques and validate the analyses by challenging macaques 6 months after immunization with the Ad26.COV2.S vaccine candidate that has been selected for clinical development. We show that Ad26.COV2.S confers durable protection against replication of SARS-CoV-2 in the lungs that is predicted by the levels of Spike-binding and neutralizing antibodies, indicating that Ad26.COV2.S could confer durable protection in humans and immunological correlates of protection may enable the prediction of durability of protection.

## Abstract (web summary)

Several COVID-19 vaccines have received emergency approval, but durability of protection is unclear. Here, the authors describe correlates of protection (CoP) for the Ad26.COV2.S vaccine in rhesus macaques and report that CoP predict the protection observed 6 months post vaccination.

## Introduction

We previously characterized the immunogenicity and protective efficacy of various Ad26-based vaccine candidates in a rhesus macaque (Macaca mulatta) challenge model of SARS-CoV-2^1–3^ that resulted in the selection of Ad26.COV2.S as the lead vaccine candidate that has recently shown an early indication of efficacy of 85% against severe/critical disease in humans with a median follow-up of participants of two months^4^. While binding and neutralizing antibody levels appear to correlate with protection against SARS-CoV-2 across multiple vaccine platforms^5^, the comparison across clinical trials is complicated by the representation of different virus variants. In addition, the proposed correlates of protection remain to be confirmed in efficacy studies. Thus, immunobridging based on a nonhuman primate model is currently especially relevant, because participants who were randomized to receive placebo in ongoing phase-3 efficacy trials are crossed over to receive study vaccine once vaccine efficacy is demonstrated, and placebo recipients are lost to follow-up due to eligibility for vaccination in national vaccine campaigns. As a consequence, it will be challenging to evaluate long-term efficacy in a blinded, placebo-controlled setting. To get an early understanding on the potential durability of protection mediated by Ad26.COV2.S, we explored whether immunological markers can also be used to predict duration of protection against SARS-CoV-2 in macaques.

Two complementary types of modeling were used to describe the relationship between an immunological marker and protection early after vaccination. The first analysis, logistic modeling (A), leads to a readily interpretable biological outcome (no detectable viral load) by only considering viral load as a binary variable. In a similar manner, this approach was previously used for anthrax^6^ and Ebola virus disease^7^ vaccines. In the second analysis (B), quantitative viral-load information was considered in a mechanistic modeling approach and the reduction of the viral load was used as a measure of protection. We subsequently assessed to what extent the constructed models could predict the outcome of challenge six months after vaccination, based on the same immunological marker measured just prior to challenge. Here, we show that Ad26.COV2.S confers durable protection against replication of SARS-CoV-2 in the lungs that is accurately predicted based on the levels of SARS-CoV-2 Spike-protein binding and neutralizing antibodies using two independent modeling approaches.

## Results

Immunogenicity and protective efficacy data from various Ad26-based vaccine candidates were used to build models to predict durability of protection against SARS-CoV-2 infection. A total of 7 Ad26-based vaccines were tested in a rhesus macaque challenge model of SARS-CoV-2^1,3^. Among these, Ad26.COV2.S is referred to as lead candidate as it is the vaccine selected for clinical development, the remaining six are referred to as prototypes or vaccine candidates as they were evaluated during the vaccine-discovery phase, but were not further selected for clinical development. A schematic representation of the three animal studies from which these data are taken is displayed in Supplementary Table 1. Immunogenicity and candidate characteristics are described elsewhere^1^. We assumed that the immune responses induced by the lead candidate (Ad26.COV2.S) and the prototypes (Ad26NCOV002, Ad26NCOV004, Ad26NCOV006, Ad26NCOV008, Ad26NCOV014, and Ad26NCOV028) are qualitatively similar, which was considered in the analysis by comparing the dataset of all Ad26-based vaccine candidates combined with that of Ad26.COV2.S alone.

Data of SARS-CoV-2 Spike (S) protein immunogenicity as assessed by pseudotyped virus-neutralization assay (psVNA) and enzyme-linked immunospot assay (ELISpot) at four weeks after vaccination were previously reported^1^. Here, we report additional spike protein enzyme-linked immunosorbent assay (S-ELISA)-binding antibody data from these studies (Supplementary Fig. 1), using the same antigen and comparable setup as the one utilized for human immunogenicity assessment^4^, to support the current correlate analysis.

### Logistic and mechanistic modeling approach

Two complementary analyses were conducted. In the first analysis (A), absence of detectable viral load was used as a measure of protection, and logistic models were built based on the relationship between immunological markers and protection. In this analysis, logistic regression was used to estimate mean probability of protection against detectable viral load (subgenomic mRNA (sgRNA)) as a function of the level of different immune responses, together with a 95% confidence interval (CI). This approach has the advantage that it is straightforward and leads to a readily interpretable biological outcome, though it disregards quantitative viral-load information. In the second analysis (B), quantitative viral-load information was considered in a mechanistic modeling approach where the reduction of the virus basic reproductive ratio (*R*_0_) below 1, which is associated with virus extinction instead of expansion, was used as a measure of protection. In this analysis, sigmoid-Emax models were used to estimate fractional reduction of *R*_0_ as a function of the level of different immunological markers, together with a 95% confidence interval (CI).

### Humoral correlates of protection against viral replication in the lung and nose based on a logistic model

**Fig. 1:**
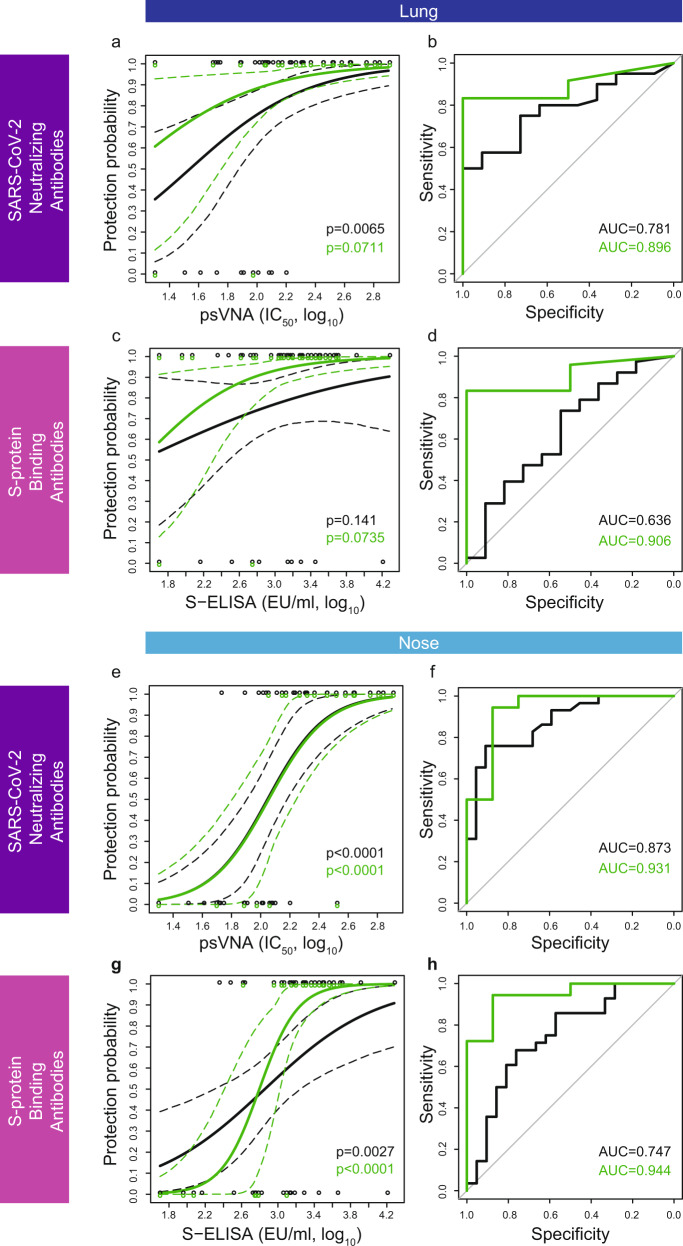
Logistic models of Ad26.COV2.S and prototype vaccine-induced levels of SARS-CoV-2-binding and -neutralizing antibodies correlate with protection against viral replication in the lung and the nose. **a**, **e** Logistic models of the correlation between the level of SARS-CoV-2-neutralizing antibodies (psVNA at four weeks after vaccination, IC_50_, log_10_) and protection against viral load in lung [constructed based on data of 81 NHP obtained in three independent experiments, (**a**)] and nose (**e**), based on the dataset of all Ad26-based vaccine candidates combined (black line) and Ad26.COV2.S alone (green line). In all, 95% confidence intervals are represented by dashed lines in the same color. Individual datapoints (*y* = 0: detectable viral load; *y* = 1 undetectable viral load) are represented by open circles in the same color. **b** ROC curves of the data presented in panel **a**. **f** ROC curves of the data presented in panel e. Area under the ROC curve (AUC) represents a measure of logistic model sensitivity and specificity. **c**, **g** Logistic models of the correlation between the level of S-protein-binding antibodies (S ELISA at four weeks after vaccination, EU/ml, log10) and protection against viral load in lung (**c**) and nose (**g**), based on the dataset of all vaccine candidates combined (black line) and Ad26.COV2.S alone (green line). In all, 95% confidence intervals are represented by dashed lines in the same color. Individual datapoints (*y* = 0: detectable viral load; *y* = 1 undetectable viral load) are represented by open circles in the same color. **d** ROC curves of the data presented in panel **c**. AUC under the ROC is indicated. **h** ROC curves of the data presented in panel **g**. AUC under the ROC is indicated. Exact p-value of the SARS-CoV-2-neutralizing antibody analysis for all Ad26-based vaccine candidates combined (black line) is 1.23 × 10^−^^06^ (**e**); exact p-value of the SARS-CoV-2-neutralizing antibody analysis for Ad26.COV2.S alone (green line) is 9.56 × 10^−05^ (**e**); exact *p*-value of the spike protein-binding antibody analysis for Ad26.COV2.S alone (green line) is 7.11 × 10^−05^ (**g**). The reported *p*-values correspond to two-sided testing of the slopes of the logistic-regression models, based on the Likelihood ratio test (chi-square test). P-values have not been corrected for multiple testing. psVNA: pseudotyped virus-neutralization assay; IC_50_: 50% neutralization antibody titer; S-ELISA: spike protein enzyme-linked immunosorbent assay; EU/mL: ELISA units per milliliter.

Higher levels of SARS-CoV-2-neutralizing antibodies were associated with increased protection against viral replication in the lung (Fig. 1a) and the nose (Fig. 1e), both for all vaccine candidates (black), and for Ad26.COV2.S alone (green). Both the model based on Ad26.COV2.S and the model based on all vaccine candidates have highly significant slopes (*p* < 0.0001) in the nose. The slope of the logistic model based on lung viral-load data for Ad26.COV2.S alone was not significantly different from 0 (*p* = 0.071), in contrast to the slope of the combined model based on all Ad26-based vaccine candidates (*p* = 0.0065). The lack of significance for Ad26.COV2.S alone is likely due to the low number of animals that had breakthrough infection in the lung (*n* = 2), which provides limited contrast for the analysis. The logistic model based on Ad26.COV2.S had similar high discriminatory capacity in both the lung and the nose, as observed by the area under the receiver-operating characteristic (ROC) curve (AUC = 0.896 in the lung and 0.931 in the nose).

Similar to neutralizing antibody levels, higher levels of S-protein-binding antibodies were associated with increased protection against viral replication in the lung (Fig. 1c) and nose (Fig. 1g). The logistic models based on the nasal viral-load data had significant slopes for both all vaccine candidates combined (black, *p* = 0.0027) and Ad26.COV2.S alone (*p* < 0.0001). The logistic models based on lung viral-load data did not have significant slopes due to the high frequency of complete protection in the lung for Ad26.COV2.S. In addition, the logistic models based on S-ELISA appear to be more sensitive to antigenic changes contributed by the different vaccine candidates than the models based on psVNA, as observed by the more pronounced differences between models based on all vaccine candidates combined compared with Ad26.COV2.S alone (to compare Fig. 1g with Fig. 1e).

Logistic models based on Ad26.COV2.S alone have a higher ROC AUC, both with regard to protection probability in the lung (Fig. 1d, AUC = 0.906 for Ad26.COV2.S vs 0.636 for all vaccine candidates) and the nose (Fig. 1h, AUC = 0.944 for Ad26.COV2.S vs 0.747 for all vaccine candidates). Thus, all logistic models based on Ad26.COV2.S alone had a higher discriminatory capacity than the models based on all vaccine candidates combined (Fig. 1b, d, f, and h). The high AUC (up to 0.944) indicates that these models should have substantial discriminatory capacity for predicting protection against viral replication. Remarkably, the logistic models based on Ad26.COV2.S alone predict 60% protection based on immune-response levels at the limit of detection, without distinction between neutralizing and binding antibodies.

### Humoral correlates of protection against viral replication in the lung and nose based on a mechanistic model

**Fig. 2:**
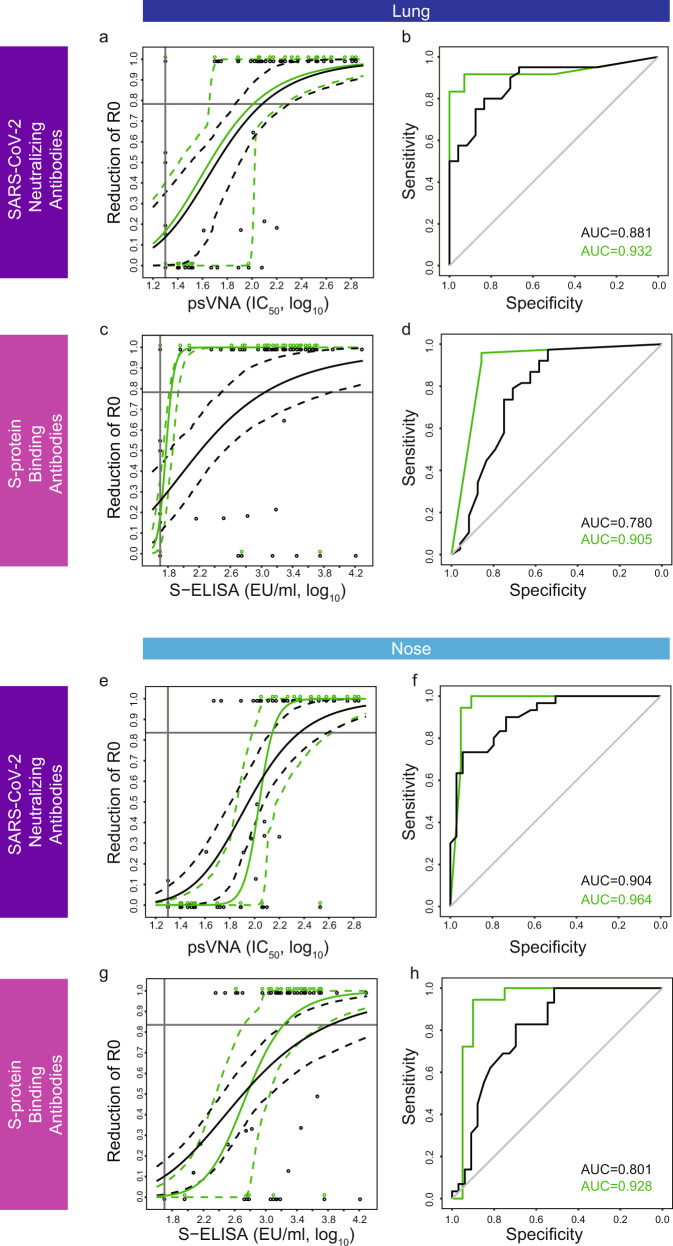
Mechanistic models of Ad26.COV2.S and prototype vaccine-induced levels of SARS-CoV-2-binding and -neutralizing antibodies correlate with basic reproductive-ratio reduction (R_0_) in the lung and the nose. **a**, **e** Sigmoid-Emax models of the correlation between the level of SARS-CoV-2-neutralizing antibodies (psVNA at four weeks after vaccination, IC_50_, log_10_) and fractional reduction of the virus basic reproductive ratio in lung (**a**) and nose (**e**), were constructed based on data of 81 NHP obtained in three independent experiments^1, 3^. The data were analyzed for all Ad26-based vaccine candidates combined (black line) and Ad26.COV2.S alone (green line). In all, 95% confidence intervals are represented by dashed lines in the same color. Individual datapoints (*y* = 0, no reduction of *R*_0_; *y* = 1, 100% reduction of R_0_) are represented by open circles in the same color. **b** ROC curves for the sigmoid-Emax models presented in panel **a**. **f** ROC curves for the sigmoid-Emax models presented in panel e. Area under the ROC curve (AUC) represents a measure of the sensitivity and specificity of the sigmoid-Emax model. **c**, **g** Sigmoid-Emax models of the correlation between the level of S-protein-binding antibodies (S ELISA at four weeks after vaccination, EU/ml, log10) and fractional reduction of the virus basic reproductive ratio in lung (**c**) and nose (**g**), based on the dataset of all vaccine candidates combined (black line) and Ad26.COV2.S alone (green line). In all, 95% confidence intervals are represented by dashed lines in the same color. Individual datapoints (*y* = 0, no reduction of *R*_0_; *y* = 1, 100% reduction of *R*_0_) are represented by open circles in the same color. **d** ROC curves for the sigmoid Emax models presented in panel **c**. AUC under the ROC is indicated. **h** ROC curves for the sigmoid Emax models presented in panel **g**. AUC under the ROC is indicated. Assay LOD is indicated by a vertical line, and *R*_0_ = 1 is indicated by a horizontal line in figure a, c, e, and g. psVNA: pseudotyped virus-neutralization assay; IC_50_: 50% neutralization antibody titer; S-ELISA: spike protein enzyme-linked immunosorbent assay; EU/mL: ELISA units per milliliter; *R*_0_: virus basic reproductive ratio.

Higher levels of SARS-CoV-2-neutralizing antibodies were associated with increased protection against viral replication in the lung (Fig. 2a) and the nose (Fig. 2e), both for all vaccine candidates (black), and for Ad26.COV2.S alone (green). The models based on Ad26.COV2.S alone had similar high discriminatory capacity in both the lung and the nose, as observed by the area under the ROC curve (AUC = 0.932 in the lung and 0.964 in the nose). These findings were obtained using a sigmoid-Emax model, and they are comparable to what has been observed using the logistic model reported above.

In addition to neutralizing antibody levels, higher levels of S-protein-binding antibodies were associated with an increased protection against viral replication in the lung (Fig. 2c) and nose (Fig. 2g). Models based on Ad26.COV2.S alone had a higher ROC AUC in the lung (Fig. 2d, AUC = 0.905 for Ad26.COV2.S vs 0.780 for all vaccine candidates) and the nose (Fig. 2h, AUC = 0.928 for Ad26.COV2.S vs 0.801 for all vaccine candidates). Thus, the models based on Ad26.COV2.S alone had a higher discriminatory capacity than the models based on all vaccine candidates combined in all instances (Fig. 2b, d, f, and h). The high AUC (up to 0.964) indicates that these models should have substantial discriminatory capacity for predicting protection against viral replication.

### Cellular correlates of protection against viral replication in the lung and nose based on logistic and mechanistic models

Cellular immune responses measured by IFN-γ ELISpot poorly correlated with protection in both lung and nose when analyzed across all vaccine candidates, both when using the logistic model (Supplementary Fig. 2a and c respectively, black curves) and using the mechanistic model (Supplementary Fig. 2e and g respectively, black curves). The dataset based on Ad26.COV2.S alone shows that higher cellular responses are associated with decreased viral load in both the lung and the nose (green curves). However, even for Ad26.COV2.S alone, the logistic models do not have a significant slope, and only the model for protection in the lung has a good discriminatory capacity (AUC = 0.875) (Supplementary Fig. 2b). Cellular immunity as measured by IFN-γ ELISpot also did not significantly improve the logistic models based on S-ELISA or psVNA in a multivariate analysis. While it cannot formally be excluded that other antigen-specific T-cell subsets would show a better correlation with protection, it is considered unlikely that these would exceed the high correlations observed for humoral responses. We therefore focused on the models for binding and neutralizing antibodies as these have a higher discriminatory capacity and are consistent between the dataset across all vaccine candidates and the dataset for Ad26.COV2.S alone.

### Results of six-month durability and efficacy study in rhesus macaques vaccinated with Ad26.COV2.S

**Fig. 3:**
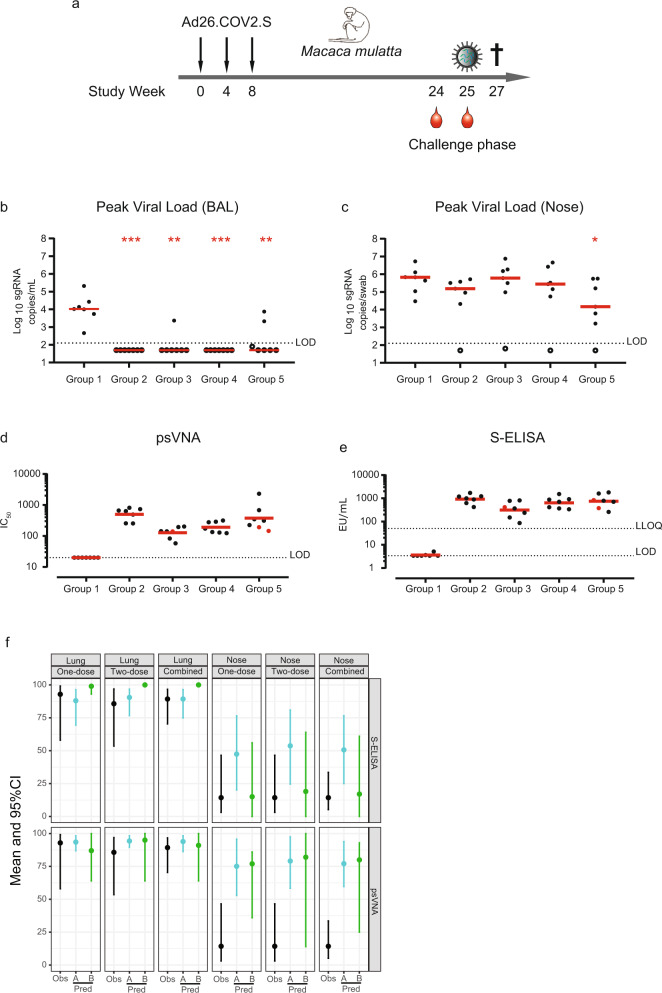
Durable protection against SARS-CoV-2 in the lower airways after vaccination with Ad26.COV2.S is predicted by binding and neutralizing antibody levels. **a** Schematic representation of the 6-month durability study. Data represent results from a single study and were obtained as technical duplicates. About 25 weeks after immunization, animals were challenged with 1 × 10^5^ TCID_50_ SARS-CoV-2 (intranasal and intratracheal routes). Peak viral loads in bronchoalveolar lavage (BAL) (**b**) and nose (**c**) were assessed by reverse-transcription PCR (RT–PCR) specific for subgenomic mRNA (sgRNA, copies/mL, log_10_). Assay limit of detection (LOD) is indicated by a dashed line. Red line represents group median. **d** SARS-CoV-2-neutralizing antibodies (psVNA,) were determined at 24 weeks post vaccination (nine days prior to challenge). The three red dots indicate the animals that had detectable viral load (>LOD) in the lung (BAL), as indicated in panel b (one animal in Group 3 and two animals in Group 5). Assay LOD is shown as a dashed line. Red line represents group geometric mean titers (GMT). **e** S-protein-binding antibody levels (S-ELISA) were determined at 25 weeks post-vaccination (one day prior to challenge). The three red dots indicate the animals that had detectable viral load (>LOD) in the lung. Assay LOD and lower limit of quantification (LLOQ) are shown as dashed lines. Red line represents group GMT. **f** Comparison of the observed (Obs, one-dose *n* = 14, two-dose *n* = 14, combined *n* = 28) protection proportion with the predicted protection probability at six months after vaccination with Ad26.COV2.S. These analyses were based on prechallenge binding (S-ELISA) and neutralizing antibody (psVNA) levels and correlates of protection models (logistic models [Pred. method A] and sigmoid Emax models [Pred. method B]) constructed on immunogenicity data obtained four weeks after Ad26.COV2.S immunization, in a total of 81 NHP from three independent studies. The statistical analysis was performed using a 2-sided Mann–Whitney U test, without correction for multiple testing. Asterisks indicate significant difference (*p < 0.05, **p < 0.01, ***p < 0.001). Exact *p*-values for panel b are: 0.00058 (Group 2), 0.00116 (Group 3), 0.000583 (Group 4), and 0.00350 (Group 5). Exact *p*-values for panel **c** are: 0.0728 (Group 2), 1 (Group 3), 0.535 (Group 4), and 0.0379 (Group 5). CI: confidence intervals, pred.: predicted, EU: ELISA units, psVNA: pseudotyped virus-neutralization assay, S-ELISA: spike protein enzyme-linked immunosorbent assay.

We subsequently assessed whether vaccination with Ad26.COV2.S provides protection in macaques at six months after the first vaccination, and whether the degree of protection could have been anticipated based on the derived correlates of protection. Groups of seven macaques were vaccinated with either a one-dose (5 × 10^10^ vp or 1 × 10^11^ vp) or two-dose regimens (5 × 10^10^ vp per dose) of Ad26.COV2.S with either a 4-week or an 8-week interval between doses (Fig. 3a and Supplementary Table 2). Twenty-five (25) weeks after the first vaccination, animals were challenged with 1 × 10^5^ TCID_50_ SARS-CoV-2 via the intranasal and intratracheal routes^8,9^. Viral loads in BAL and nasal swabs were assessed by reverse-transcription PCR (RT–PCR) specific for sgRNA, which predominantly detects replicating virus^8,10^. All controls had detectable virus in the upper and lower airways at comparable peak levels as in the previous studies^1,3^. Ad26.COV2.S-vaccinated macaques were highly protected against viral replication in the lung six months after the first vaccination (Fig. 3b) and the limited lung-breakthrough infection in three out of 28 macaques appeared to be unrelated to either a single- or two-dose vaccine regimen being applied (Supplementary Fig. 3b to e). In contrast, most vaccinated animals (24 out of 28) had detectable virus in the nose (Fig. 3c and Supplementary Fig. 4b to e), though the duration of virus shedding was significantly lower in the one-dose 1 × 10^11^ vp group (*p* = 0.012, Group 2) and in the 8-week 2-dose regimen group (*p* = 0.012, Group 5) (Supplementary Fig. 4f). This latter group also showed significantly lower peak viral load (*p* = 0.038) compared with the control group (Fig. 3c). The outcome of statistical analysis is shown in Supplementary Table 3.

Immune-response kinetics of this cohort up to 14 weeks after the first immunization are described elsewhere^2^. For the purpose of this project, we only consider the levels of SARS-CoV-2-neutralizing antibodies (Fig. 3d) and S-protein-binding antibodies (Fig. 3e), measured just prior to SARS-CoV-2 challenge, using the same assays used in the construction of both the logistic and the sigmoid-Emax models.

### Validation of logistic and mechanistic models in the lung and nose with the six-month durability and efficacy study data

Data from the durability study were grouped by one-dose regimes (one dose, *n* = 14), two-dose regimens (two dose, *n* = 14), or analyzed together (combined, *n* = 28). We focus on models based on Ad26.COV2.S, as this is the same vaccine candidate as used in the durability study. For each group, the observed proportion of protection (Obs) (indicated with a black dot in Fig. 3f), together with its 95% CI (indicated with black-line interval in Fig. 3f) after challenge is compared with the predicted (Pred) mean probability of protection and a bootstrap-derived 95% CI, based on their prechallenge immunogenicity results and the previously developed logistic-regression models (analysis A, blue dot and blue-line interval in Fig. 3f) or sigmoid-Emax model (analysis B, green dot and green-line interval in Fig. 3f).

For the one-dose regimens, there is excellent agreement between the prediction from the logistic and sigmoid-Emax regression model and the observed protection probabilities in the lung (Table 1), both when based on S-protein-binding antibodies [Pred-A: 88.0% (69.4–96.4%)] vs Pred-B [99.0% (93.0–100%)] vs Obs [92.9% (58.0–99.2%)] as well as based on neutralizing antibodies [Pred-A: 93.5% (86.9–98.3%)] vs Pred-B [87.0% (64.0–100%)] vs Obs [92.9% (58.0–99.2%)]. The observed proportion of the protection for the two-dose regimens is similar, and within the 95% CI of the predicted probability for S-protein-binding antibodies, when based on approach A [Obs: 85.7% (53.5–96.9%) vs Pred-A: 90.5% (76.7–96.8%)]. The observed proportion of the protection for the two-dose regimens [Obs: 85.7% (53.5–96.9%)] is slightly lower than predicted using approach B [100% (100–100%)]. Based on neutralizing antibodies, both the logistic model and the mechanistic model are strikingly close to the observed protection [Obs: 89.3% (70.4–96.7%), Pred-A: 93.9% (86.3–98.3%), and Pred-B: 91.0% (64.0–100%)]. Overall, the predictions based on binding and neutralizing antibodies show a remarkable correspondence to the observed protection proportion in the lung, indicating that the potential correlates of protection identified early after vaccination can be used to predict durable protection against infection of the lower airways in rhesus macaques.

**Table 1:** Comparison of the observed protection proportion with the predicted protection probability using logistic (analysis A) and sigmoid Emax (analysis B) models.

		Lung one-dose	Lung two-dose	Lung combined	Nose one-dose	Nose two-dose	Nose combined
S-ELISA	Observed (Mean)	92.9%	85.7%	89.3%	14.3%	14.3%	14.3%
	[95% CI]	[58.0–99.2%]	[53.5–96.9%]	[70.4–96.7%]	[3.1–46.5%]	[3.1–46.5%]	[5.2–33.5%]
	Prediction analysis A (Mean)	88.0%	90.5%	89.3%	47.4%	53.7%	50.6%
	[95% CI]	[69.4–96.4%]	[76.7–96.8%]	[74.9–96.4%]	[20.1–76.5%]	[24.6–80.9%]	[25.0–76.6%]
	Prediction analysis B (Mean)	99.0%	100%	100%	15.0%	19.0%	17.0%
	[95% CI]	[93.0–100%]	[100–100%]	[100–100%]	[0.0–56.0%]	[0.0–64.0%]	[0.0–61.0%]
psVNA	Observed (Mean)	92.9%	85.7%	89.3%	14.3%	14.3%	14.3%
	[95% CI]	[58–99.2%]	[53.5–96.9%]	[70.4–96.7%]	[3.1–46.5%]	[3.1–46.5%]	[5.2–33.5%]
	Prediction analysis A (Mean)	93.5%	94.3%	93.9%	75.1%	79.1%	77.1%
	[95% CI]	[86.9–98.3%]	[89.5–98.3%]	[86.3–98.3%]	[52.9–95.7%]	[58.4–97.5%]	[59.7–93.9%]
	Prediction analysis B (Mean)	87%	95.0%	91.0%	77.0%	82.0%	80.0%
	[95% CI]	[64.0–100%]	[64.0–100%]	[64.0–100%]	[36.0–86.0%]	[14.0–100%]	[25.0–93.0%]

psVNA pseudotyped virus neutralization assay, S-ELISA spike protein enzyme-linked immunosorbent assay.

The observed protection probability in the nose is substantially lower than predicted based on the logistic model (Analysis A) using either prechallenge binding or neutralizing antibody levels in the circulation. While the predicted protection probability was approximately 50% based on S-protein-binding antibody levels and 75% based on neutralizing antibody levels, nose protection was only observed in 4/28 animals (14.3%). Based on neutralizing antibody levels, the Sigmoid Emax models (Analysis B) also over-predict protection in the nose [80% (25.0–93.0%), combined], suggesting that systemic binding and neutralizing antibody levels likely are associated with a distinct mechanistic correlate of protection in the nose early after vaccination, rather than being a mechanistic correlate of protection themselves^11,12^. However, the predicted protection probability based on the sigmoid-Emax model was approximately 17% (0.0–61.0%, combined) based on S-protein-binding antibody levels, in line with the observed protection, indicating that the sigmoid-Emax model based on S-protein-binding antibodies may more accurately predict protection in the nose than the logistic models. The basic reproductive ratio (*R*_0_) is approximately 1.5-fold higher in the nose than in the lung, indicating that higher antibody levels are required for protection in the nose. The lower degree of protection in the nose could indicate an earlier waning of protection against upper airway-disease, while protection against lower-airway disease is maintained.

Predictions for the probability of protection, using logistic models (A) based on data of all vaccine candidates combined (Supplementary Fig. 5) largely confirm predictions based on Ad26.COV2.S (Fig. 3f), demonstrating the robustness of predicting durable protection in the lower airways based on binding and neutralizing antibody levels. Predictions based on neutralizing antibodies at six months and the mechanistic model (B) built on data from all vaccine candidates were also similar to those based on the model based on data of Ad26.COV2.S vaccination alone. However, the predictions based on binding antibody data of all the candidates underpredicted the protection observed at six months. Given that the observations were only based on data after the Ad26.COV2.S vaccine, the results show that the other vaccine candidates would require higher binding antibody concentrations to control the virus than those required by Ad26.COV2.S, reinforcing the selection of Ad26.COV2.S for full development.

## Discussion

One or two doses of Ad26.COV2.S confer a high degree of protection against SARS-CoV-2 replication in the lower airways of macaques for at least six months. It is conceivable that this would translate into durable protection against COVID-19 in vaccinated humans, where protection may be even more durable as exposure with SARS-CoV-2 is likely to trigger a beneficial anamnestic response^13^, which may contribute to protection due to the incubation time between viral exposure and development of symptoms. In the macaque SARS-CoV-2 model used here, an anamnestic response will have no added value for protection, due to the relatively high-challenge virus inoculum resulting in immediate upper and lower respiratory-tract infection, and rapid clearance of the virus. Accordingly, durable protection in macaques could be predicted based on circulating antibody levels prior to virus inoculation using two different modeling approaches. Passive transfer studies using convalescent or vaccinee serum recently confirmed antibody responses as one correlate of protection in rhesus macaques^14,15^. Binding and neutralizing antibody levels appear to be a more universal correlate across multiple vaccine platforms^15^ and may allow prediction of protection beyond six months. Ad26.COV2.S has recently shown an early indication of efficacy of 85% against severe/critical disease in humans with a median follow-up of participants of two months^4^. Based on immunological follow-up, it will be ascertained whether predictions based on the macaque model are in line with observed protection in humans. If confirmed, or when correlates of protection are identified in humans, durability of protection in humans could be estimated based on immunogenicity data. This will be especially relevant in the current situation where participants who were randomized to receive placebo in ongoing phase-3 efficacy trials are crossed over to receive study vaccine, or when placebo recipients are lost to follow-up due to eligibility for vaccination in national vaccine campaigns and long-term efficacy can no longer be evaluated in a blinded, placebo-controlled setting. Finally, if it can be established how efficacy in macaques relates to clinical benefit, macaque models could be used to show a likelihood of protection against new SARS-CoV-2 variants for which placebo-controlled trials can no longer be easily conducted.

## Methods

### Statistical methods: analysis A (logistic modeling)

Peak and duration of viral load in BAL and nasal-swab samples of each vaccinated group were compared with the control group using Mann–Whitney U test.

Protection outcome is binary and driven by the viral-load observation (i.e., defined as 0 if the macaque had a detectable viral load as measured by sgRNA and as 1 if the macaque had an undetectable viral load). The effect of the different immunogenicity-response markers on the measure of protection is investigated using a logistic-regression modeling approach. Penalized logistic models were built using Firth’s method^16^, with protection outcome as the dependent variable and immune-response level as the independent variable. A bootstrap procedure was applied where the dataset was resampled 10,000 times with replacement and the logistic model was fitted to each resampled dataset. The 2.5th and 97.5th percentiles of the fitted protection probabilities were calculated over a grid of immune-response values to generate the pointwise 95% confidence band.

The above-developed logistic model can be used to predict the probability of protection for a set of newly measured immunogenicity samples for which we want to estimate their probability of protection without having the actually observed protection status, an example of such a set of new samples are the prechallenge immunogenicity results for the vaccinated animals. This was done using a double-bootstrap procedure, to capture both the variation contributed by the model dataset and the variation contributed by the test dataset, ensuring that the precision of the prediction is accurately reflected. In this procedure, both the macaque dataset used to fit the logistic model and the dataset with new samples of interest are resampled 10,000 times each with replacement. The logistic-regression model describing the effect of the immunogenicity marker on the measure of protection, is refitted to each resampled macaque dataset and used to predict the probability of protection for the resampled dataset with new samples. As a result, 10,000 mean-predicted protection probabilities for the resampled dataset with new samples are obtained. The 95% CI is then derived by taking the 2.5^th^ and 97.5^th^ percentiles of the 10,000 mean-predicted protection probabilities.

The estimated mean population probability of protection for the challenged macaques, together with a 95%CI based on their viral load measurements after challenge, is analyzed using a logistic-regression model with overall intercept only using the glm functionality (lme4 package R)^17^, and compared with the observed proportion of protection.

Simulations reported here were conducted using R version 3.6.0^18^ on an x86_64-redhat-linux-gnu (64-bit) platform running under a Red Hat Enterprise Linux Server 7.4 (Maipo) using Rstudio Version 1.1.453^19^. The seed was set to allow reproducibility.

### Statistical methods: analysis B (mechanistic modeling)

The time course of the viral load in BAL and nasal-swab samples of each vaccinated and nonvaccinated animal was analyzed with published mixed-effect viral kinetic model^20^ using NONMEM software. After estimation of the model parameters, the *R*_0_ was defined as the product of the production rates of the target and infected cells and of the virions divided by the product of the decay rates of the infected cells and virions^20,21^. Of note, *R*_0_ in the nose was approximately 1.5-fold higher than in the lung, indicating that higher levels of immunological markers are required for protection in the nose than in the lung. The effect of the immunological markers measured 28 days after vaccination on reducing the basic reproductive ratio (*R*_0_) was modeled using a sigmoid-Emax model. The minimal immunogenicity-response marker (MIM) to SARS-COV-2 vaccine that reduces the *R*_0_ < 1 at day 28 was determined as EC50 * (*R*_0_−1)^(1/h), where EC50 represents the concentration of the immunogenicity-response marker that reduces *R*_0_ to half and h is the Hill coefficient of the sigmoid-Emax model.

The above model was used to predict the probability of *R*_0_ < 1 (i.e., virus extinction instead of growth) for a set of prechallenge immunogenicity-response makers for the vaccinated animals. This was done using the bootstrap procedure in R software. In this procedure, the macaque dataset used for model fitting and to determine the MIM was resampled 1000 times each with replacement. As a result, 1000 MIM were used to predict the 1000 predicted probabilities of *R*_0_ < 1 for the resampled dataset. The mean population probability and its 95% CI (derived by taking the 2.5th and 97.5th percentiles of the 1000 predicted probabilities of *R*_0_ < 1) were compared with the observed and predicted proportion of protection.

The viral kinetic analysis was performed using NONMEM 7.4 (ICON plc, Hanover, MD, USA) in a Windows 7 operating system. Perl-speaks-NONMEM (PsN, version 4.2.0, [http://psn.sourceforge.net/docs.php]) was used to run NONMEM whenever possible. Data management, exploratory analyses, diagnostic graphics, and postprocessing of the data and NONMEM outputs, as well as simulations reported here were conducted using R version 3.6.2^18^ on an x86_64-pc-linux-gnu (64-bit) platform running under a Red Hat Enterprise Linux Server 7.4 (Maipo) using Rstudio Version 1.2.1335-1^19^ The seed was set to allow reproducibility.

### Description of the 6-month durability study

The NHP study of adult animals was conducted at Charles River Laboratories Montreal ULC, Laval Site (CA). Animals were obtained from Kunmings Biomed International Ltd, China. Prior to transfer from the test-facility colony, all animals were subjected to a health assessment. The evaluations were performed in accordance with standard operating procedures by technical staff. Animal experiment approval was provided by the Institutional Animal Care and Use Committee at Charles River Laboratories Montreal ULC, Laval Site (CA). Animal experiments were performed in compliance with guidelines published by the Canadian Council on Animal Care and the Guide for the Care and Use of Laboratory Animals published by the National Research Council Canada. The Test Facility is accredited by the Canadian Council on Animal Care and the American Association for Accreditation of Laboratory Animal Care. In addition, the study was conducted according to European Medicines Agency guideline International Conference on Harmonization M3(R2): Guidance on Non- Clinical Safety Studies for the Conduct of Human Clinical Trials and Marketing Authorization for Pharmaceuticals and Food and Drug Administration guideline, Redbook 2000: General Guidelines for Designing and Conducting Toxicity Studies.

Sixty (60) (57 females and three males, three males were allocated to test Groups 3, 4, and 5, 1 male in each group) rhesus macaques (M. mulatta) from Chinese origin, between 3.3 and 5.0 year of age and weighing between 2.9 and 8.1 kg, were assigned to five groups by a randomizing stratification system based on body weights. In total, 14 animals were included in each vaccine regimen, and four animals were included in the sham-control group. Group 1 (*n* = 4) is the sham-control group and received saline injection at Week 0 and Week 8. Groups 2 and 3 (*n* = 14 in each group) received one immunization with 1 × 10^11^ vp and 5 × 10^10^ vp of Ad26.COV2.S, respectively, at Week 0. Groups 4 and 5 (*n* = 14 in each group) received two immunizations with 5 × 10^10^ vp of Ad26.COV.2 spaced by 4 (Week 0 and Week 4) and eight weeks (Week 0 and Week 8), respectively. All immunizations were performed via the intramuscular route in the quadriceps muscle of the left hind leg. Blood for serum was obtained before the first vaccine dose and every two weeks subsequently up to Week 14 of the study. Before the study ended, three treatment naïve animals were added to the study, were allocated to an independent group and not included in the immunogenicity analysis. Immunogenicity data up to week 14 were previously reported elsewhere^2^.

In total, 32 animals included in the immunogenicity analysis, together with the three treatment-naive animals (total of 35 animals) were transferred on Week 19 to a United States Department of Agriculture (USDA) quarantine facility in the US (Primera Science Center LLC, Florida) where they stayed for a minimum quarantine period of 35 days. On Week 24, the NHPs were transferred from Primera to Bioqual for SARS-CoV-2 challenge study permitted by the Bioqual Institutional Animal Care and Use Committee, 12301, Parklawn Drive, Rockville, MD 20852. On Week 25, the animals were inoculated with SARS-CoV-2 USA-WA1/2020, six months after receiving the first immunization/injection at Charles River Laboratories. In total, five groups of seven rhesus macaques received either one dose of Ad26.COV2.S [1 × 10^11^ vp (Group 2) or 5 × 10^10^ vp (Group 3)] or two doses of Ad26.COV2.S (5 × 10^10^ vp/dose) with either a 4-week (Group 4) or an 8-week (Group 5) interval between doses. A group of 7 controls (Group 1) was included in the study, of which four received two doses of saline, and three were treatment naive. By Week 27, the in-life period of the challenge study ended, and all 35 animals were sacrificed. Whole blood, serum, bronchoalveolar lavage, and nasal-swab samples collected during the challenge study and at sacrifice were shipped to Beth Israel Deaconess Medical Center (BIDMC) for analysis.

### SARS-CoV-2 pseudotyped virus neutralization assay on HEK293T-hACE2 cells

The SARS-CoV-2 pseudoviruses expressing a luciferase reporter gene are generated in an approach similar to that as described previously^8,9,22^. Briefly, the packaging construct psPAX2, luciferase reporter plasmid pLenti-CMV Puro-Luc, and spike protein expressing pcDNA3.1-SARS CoV-2 SΔCT are cotransfected into HEK293T cells. The supernatants containing the pseudotype viruses are collected and stored at -80 °C until use. To determine the neutralization activity of the antisera from vaccinated animals, HEK293T-hACE2 stable cells are seeded in 96-well tissue culture plates. Serial dilutions of serum samples are prepared and mixed with pseudovirus. The mixture is incubated at 37 °C for 1 h before adding to HEK293T-hACE2 cells. About 48 h after infection, cells are lysed in Steady-Glo Luciferase Assay according to the manufacturer’s instructions. SARS-CoV-2-neutralization titers are defined as the sample dilution at which a 50% reduction in RLU is observed relative to the average of the virus control wells. Assay LOD = 20 IC50 (1.3 log10). This assay was previously shown to strongly correlate with wtVNA and SARS-CoV-2 Spike IgG ELISA^2^.

### SARS-CoV-2 Spike IgG ELISA

SARS-CoV-2 Spike-specific binding antibody concentrations were determined using the human SARS-CoV-2 Spike immunoglobulin G (IgG) ELISA. The SARS-CoV-2 antigen used is a stabilized prefusion spike protein ((2 P), Δfurin, T4 foldon, His-Tag) produced in ES-293 cells. The ELISA was performed at Nexelis (Seattle, WA, USA).

In brief, the SARS-CoV-2 Pre-Spike IgG ELISA is an indirect ELISA that is based on the antibody/antigen interactions. Purified SARS-CoV-2 Pre-Spike Antigen is adsorbed to the wells of a microplate. Diluted serum samples (test samples, standard, and quality controls) are added in the wells. Anti-SARS-CoV-2 Pre-Spike antibodies if present in the serum samples bind to the immobilized SARS-CoV-2 Pre-Spike antigen. Unbound sample is then washed from the wells, and enzyme-conjugated anti-human IgG is added. The anti-human IgG enzyme conjugate binds to the antigen–antibody complex. Excess conjugate is washed away, and 3,3′,5,5′-Tetramethylbenzidine (TMB) colorimetric substrate is added. Bound enzyme catalyzes a hydrolytic reaction, which causes color development. After the established time period, the reaction is stopped. The intensity of the generated color is proportional to the amount of anti-SARS-CoV-2 Pre-Spike antibodies bound to the wells. The optical density results are read on a spectrophotometer (ELISA plate reader). A reference standard on each tested plate is used to quantify the amount of antibodies against SARS-CoV-2 Pre-Spike in the sample according to the unit assigned by the standard (ELISA Laboratory Unit per milliliter: ELU/mL). Assay LOD is 3.4 EU/ml (0.53 log_10_ EU/ml) and LLOQ is 50.3 EU/ml (1.7 log_10_ EU/ml). This assay was previously shown to strongly correlate with wtVNA and psVNA^2^.

### Viral load determination

The presence of virus in BAL and nasal-swab samples is measured by real-time polymerase chain reaction (RT-PCR) of SARS-CoV-2 E-gene subgenomic ribonucleic acid (sgmRNA). The assay (based on Wölfel et al., 2020^10^) thus detects replicating virus and largely distinguishes between virus present in the inoculum, and infected cells. A standard curve is generated using RNA transcribed from the SARS-CoV-2 E-gene sgmRNA cloned into a pcDNA3.1 expression plasmid. Prior to RT-PCR, samples are thawed at room temperature or 4 °C and reverse-transcribed using Superscript III VILO (Invitrogen) according to the manufacturer’s instructions. The primer sequences were ordered as a Taqman custom gene expression assay (Thermo Fisher Scientific; Forward primer: sgLeadSARSCoV2-F, CGATCTCTTGTAGATCTGTTCTC, Reverse primer: E_Sarbeco_R, ATATTGCAGCAGTACGCACACA, Probe: E_Sarbeco_P1, VIC-ACACTAGCCATCCTTACTGCGCTTCG-MGBNFQ)^10^. The complementary deoxyribonucleic acid (cDNA) is stored at 4°C until use. A Taqman custom gene expression assay (ThermoFisher Scientific) was designed using the sequences targeting the E-gene sgmRNA. The PCR is carried out on a QuantStudio 6 and 7 Flex Real-Time PCR System (Applied Biosystems) according to the manufacturer’s specifications. Standard curves were used to calculate sgRNA copies and were multiplied by 125 to get to copies per mL or per swab. Quantitative sensitivity (LOQ) per PCR reaction was determined as one copy, corresponding to a limit of detection (LOD) of 125 copies per sample.

### Reporting summary

Further information on research design is available in the Nature Research Reporting Summary linked to this article.

## Supplementary information

Supplementary Information

Reporting Summary

## Data Availability

The data-sharing policy of Janssen, Pharmaceutical Companies of Johnson & Johnson, is available at https://www.janssen.com/transparency. All data presented in the paper are available in Supplementary Tables 4, 5, 6, 7, 8, 9, 10 and 11.
