# Supplementary material for: SARS-CoV-2 binding and neutralizing antibody levels after Ad26.COV2.S vaccination predict durable protection in rhesus macaques
Source: Nat Commun. 2021 Oct 7;12:5877. doi: 10.1038/s41467-021-26117-x (PMC8497464; doi:10.1038/s41467-021-26117-x)
Supplement: Supplementary file 1 — Supplementary Information [file 41467_2021_26117_MOESM1_ESM.pdf]

SUPPLEMENTARY INFORMATION

SARS-CoV-2 binding and neutralizing antibody levels after Ad26.COV2.S vaccination predict durable protection in rhesus macaques

Ramon Roozendaal[1#], Laura Solforosi[1#], Daniel J. Stieh[1#], Jan Serroyen[1], Roel Straetemans[2], Anna Dari[2], Muriel Boulton[2], Frank Wegmann[1], Sietske K. Rosendahl Huber[1], Joan E. M. van der Lubbe[1], Jenny Hendriks[1], Mathieu Le Gars[1], Liesbeth Dekking[1], Dominika N. Czapska-Casey[1], Nuria Guimera[1], Sarah Janssen[1], Sarah Tete[1], Abishek Chandrashekar[3], Noe B. Mercado[3], Jingyou Yu[3], Wouter Koudstaal[4], Juan J. Perez-Ruixo[2], Jerry Sadoff[1], Dan H. Barouch[3,5,6,7], Hanneke Schuitemaker[1], Roland Zahn[1*]

[1]Janssen Vaccines & Prevention B.V., Leiden, The Netherlands

[2]Janssen R&D, Beerse, Belgium

[3]Center for Virology and Vaccine Research, Beth Israel Deaconess Medical Center, Harvard Medical School, Boston, MA, USA.

[4]Lucidity Biomedical Consulting, , Granada, Spain.

[5]Ragon Institute of MGH, MIT and Harvard, Cambridge, MA, USA

[6]Harvard Medical School, Boston, MA, USA

[7]Massachusetts Consortium on Pathogen Readiness, Boston, MA, USA.

[#]These authors contributed equally

*Corresponding Author: Roland Zahn (RZahn@its.jnj.com)

SUPPLEMENTARY FIGURES

Supplementary Figure 1: S-protein binding antibody levels from preceding vaccine studies

Supplementary Figure 2: Correlation of cellular immune responses with protection against SARS-CoV-2 in the lung and the nose

Supplementary Figure 3: Viral load kinetics and duration in the lungs of individual animals of the six-month durability study

Supplementary Figure 4: Viral load kinetics in the nose of individual animals of the six-month durability study

Supplementary Figure 5: Comparison between observed and predicted protection based on correlate models obtained with data of all vaccine candidates combined

SUPPLEMENTARY TABLES

Supplementary Table 1: Schematic representation of the three NHP studies used to build the models

Supplementary Table 2: Six-month durability study design

Supplementary Table 3: Statistical comparison between vaccinated vs control groups in the six-month durability study

Supplementary Table 4: Source data file for Figure 1

Supplementary Table 5: Source data file for Figure 2

Supplementary Table 6: Source data file for Figure 3

Supplementary Table 7: Source data file for Supplementary Figure 1

Supplementary Table 8: Source data file for Supplementary Figure 2

Supplementary Table 9: Source data file for Supplementary Figure 3

Supplementary Table 10: Source data file for Supplementary Figure 4

Supplementary Table 11: Source data file for Supplementary Figure 5

SUPPLEMENTARY FIGURES

Supplementary Figure 1: S-protein binding antibody levels from preceding vaccine studies

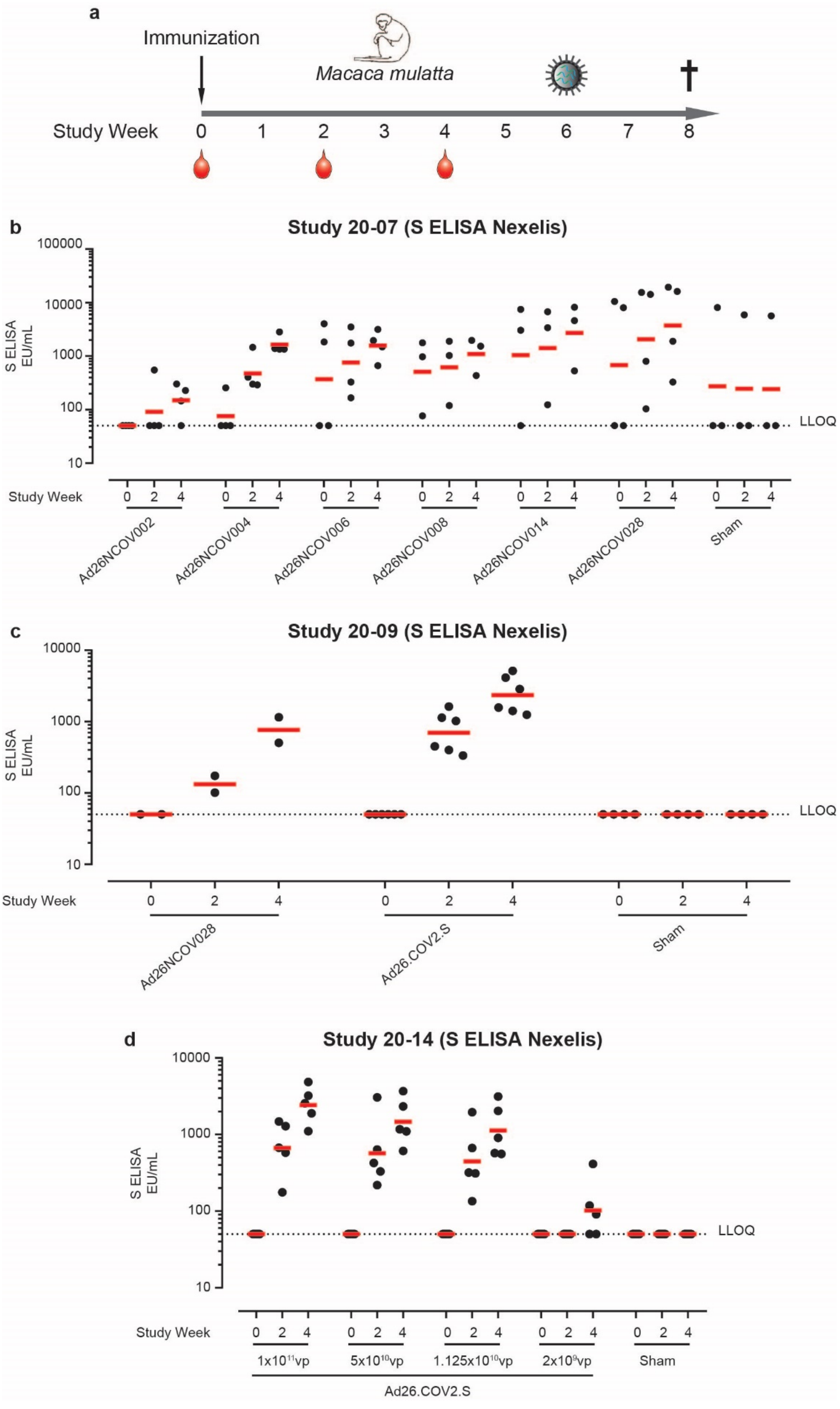

(a) Schematic representation of studies 20-07, 20-09 and 20-14, three independent NHP studies testing the immunogenicity and efficacy of Ad26-based vaccine candidates. A total of 52 monkeys received a one-dose regimen of Ad26 based vaccine candidates. In total a group of 30 unvaccinated controls was included in the studies. Six weeks after the vaccination animals were challenged with $1x10^5$ $TCID_{50}$ SARS-CoV-2 via the intranasal and intratracheal routes. Ad26 constructs: tPA.S (Ad26NCOV002), full length spike protein (S) in which the wild-type (wt) signal peptide (SP) is replaced by tissue plasminogen activator (tPA) SP; tPA.S.PP (Ad26NCOV004), full length S in which SP is replaced by tPA and in which the furin cleavage site mutations and proline substitutions (K986P, V987P) have been introduced; S (Ad26NCOV006), native full length spike protein; S.dCT (Ad26NCOV008), full length S in which the cytoplasmic tail (CT) is deleted; tPA.WT.S (Ad26NCOV014), full-length S in which tPA SP is added upstream of the wt SP; S.dTM.PP (Ad26NCOV028), full length S in which the transmembrane domain (TM) is deleted and the furin cleavage site mutations and proline substitutions (K986P, V987P) have been introduced; S.PP (Ad26NCOV030, later named Ad26.COV2.S), full length S in which furin cleavage site mutations and proline substitutions have been introduced.. **(b, c, d)** S-protein binding antibody levels (S ELISA, EU/ml, log10) were determined at baseline, 2-, and 4-weeks post-vaccination. Some animals in the candidate selection study (20-07) had background reactivity prior to vaccination (Week 0). Background reactivity was not observed in psVNA, and likely represents aspecific binding. Assay LLOQ is shown as dashed lines. Data were obtained as technical duplicates. Horizontal bars indicate geometric mean of response within each group.

Supplementary Figure 2: Correlation of cellular immune responses with protection against SARS-CoV-2 in the lung and the nose.

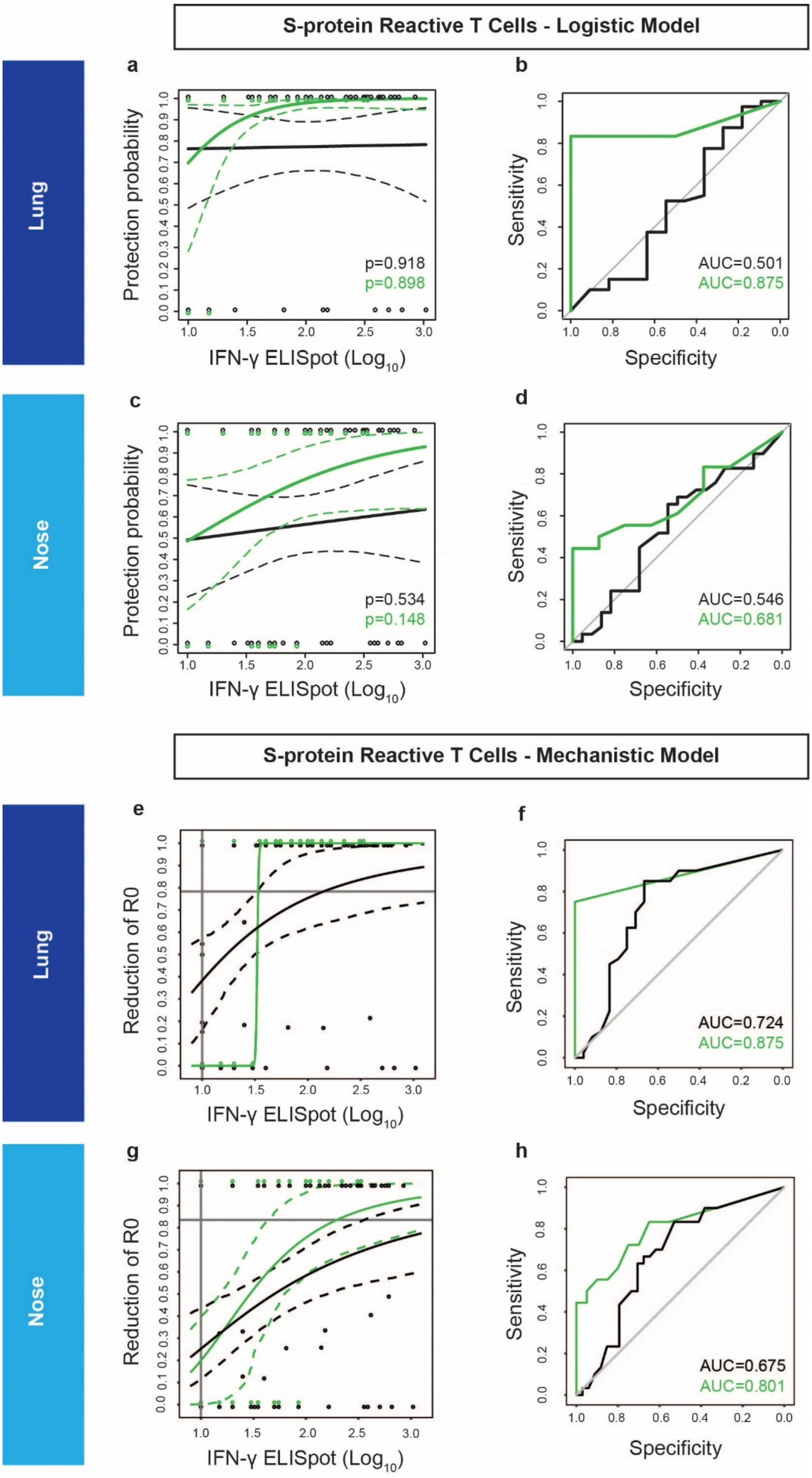

(**a, c**) Logistic models of the correlation between the level of S-protein specific T cells (IFN-gamma ELISpot, SFU/10^6 PBMC, $\log_{10}$) and protection against viral load in lung (a, BAL) and nose (c, swabs), based on the dataset of all Ad26-based vaccine candidates combined (black line) and Ad26.COV2.S alone (green line), a total of 81 NHP from 3 independent experiments. 95% confidence intervals are represented by dashed lines in the same color. Individual datapoints (y=0: detectable viral load; y=1 undetectable viral load) are represented by open circles in the same color. (**b, d**) ROC curves for the logistic models presented in panels a and c, respectively. Area under the ROC curve (AUC) is indicated and represents a measure of the sensitivity and specificity of the logistic model. (**e, g**) Mechanistic models of the correlation between the level of S-protein specific T cells (IFN-gamma ELISpot, SFU/10^6 PBMC, $\log_{10}$) and protection against viral load in lung (e, BAL) and nose (g, swabs), based on the dataset of all Ad26-based vaccine candidates combined (black line) and Ad26.COV2.S alone (green line). 95% confidence intervals are represented by dashed lines in the same color. Individual datapoints are represented by open circles in the same color. (**f, h**) ROC curves for the mechanistic models presented in panels e and g, respectively. Area under the ROC curve (AUC) is indicated and represents a measure of the sensitivity and specificity of the logistic model. The reported p-values correspond to two-sided testing of the slopes of the logistic regression models, based on the Likelihood ratio test (chi-square test). P-values have not been corrected for multiple testing.

Supplementary Figure 3: Viral load kinetics and duration in the lungs of individual animals of the 6M durability study

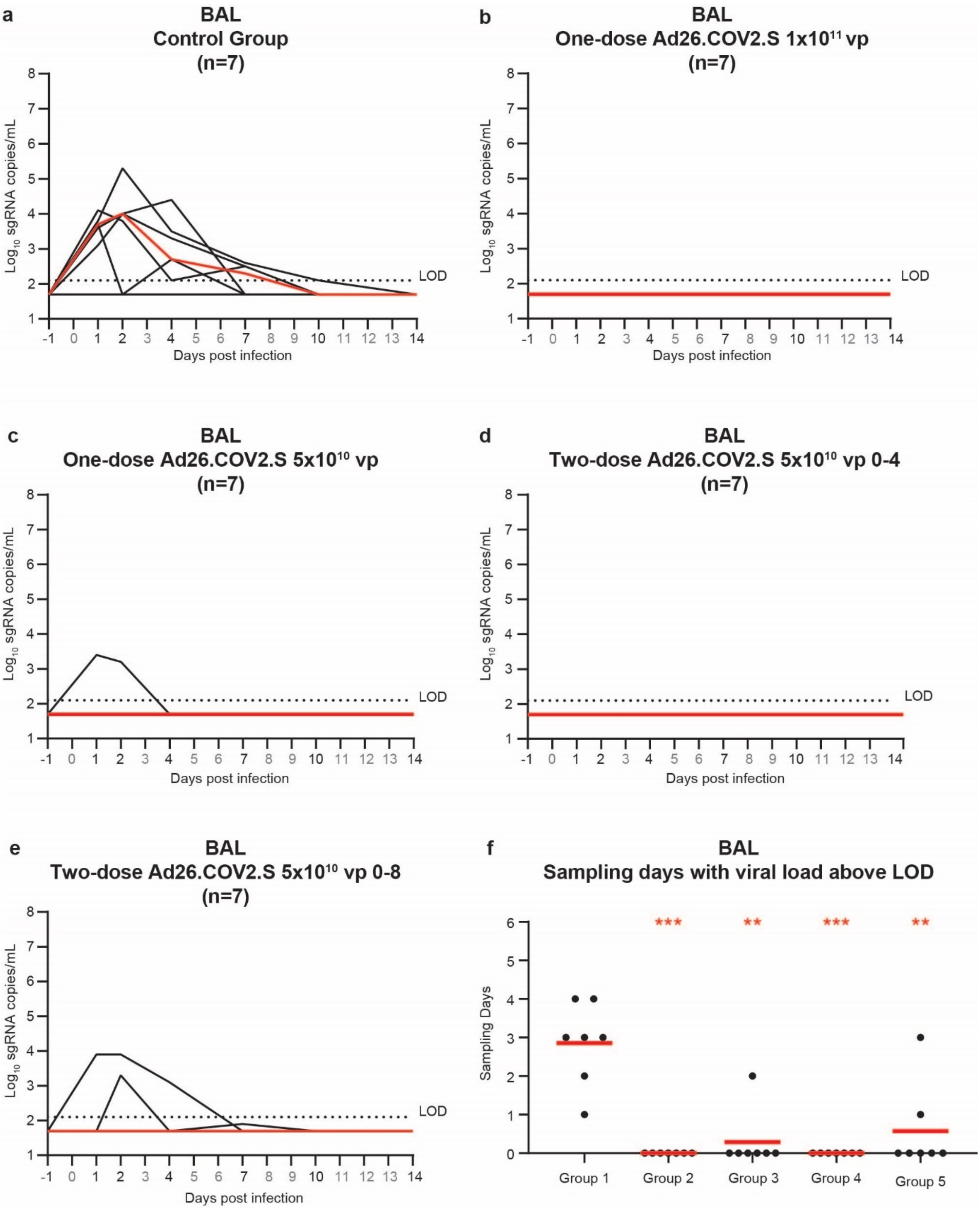

(**a**-**e**) Six months (25 weeks) post immunization the NHPs were inoculated with SARS-CoV-2. Viral load was analyzed in BAL samples taken from NHPs immunized with a single dose of Ad26.COV2.S at a dose level of $1x10^{11}$ vp (N=7; Group 2; panel **b**), a single dose of Ad26.COV2.S at a dose level of $5x10^{10}$ vp (N=7; Group 3; panel **c**), two doses of Ad26.COV2.S at a dose level of $5x10^{10}$ vp with a 4-week interval (N=7; Group 4; panel **d**), two doses of Ad26.COV2.S at a dose level of $5x10^{10}$ vp with an 8-week interval (N=7; Group 5; panel **e**) or control animals (N=4 immunized with saline, N=3 treatment naïve; Group 1; panel **a**) at Day -1, 1, 2, 4, 7, 10 and at sacrifice (Day 13 or 14 depending on animal cohort assignment) after SARS-CoV-2 challenge. In these graphs viral load values observed at sacrifice are shown at Day 14 for all animals. RNA was extracted from BAL, followed by RT-qPCR using sequences targeting the E gene sgRNA of virus replication cellular intermediates. LOD is set at 125 copies/mL (Log_{10} = 2.1) based on the limit of detection being 1 RNA copy per reaction multiplied by 125 to correct for sample dilutions. The median viral load per group is indicated in red, viral load in individual animals is indicated in black. On the x axis, days post infections on which samples were collected are shown in black; days post infections on which samples were not collected as per study plan are shown in grey. **(f)** Duration (days) of viral load in BAL samples after SARS-CoV-2 challenged of control and vaccinated macaques. Black dots represent individual animals, red lines the group mean. Data represent results from a single study, and were obtained as technical duplicates. The statistical analysis was performed using a 2-sided Mann-Whitney U test, without correction for multiple testing. Red asterisks indicate the statistical significance. (*p<0.05, ** p<0.01, *** p<0.001). Exact p-values for panel **f** are 0.000583 for Group 2, 0.00175 for Group 3, 0.000583 for Group 4 and 0.00641 for Group 5.

BAL = bronchoalveolar lavage; LOD = Limit of detection; LRT = lower respiratory track; N = number of animals; NHPs = nonhuman primates; RNA = ribonucleic acid; RT-qPCR = reverse transcriptase quantitative polymerase chain reaction; sgRNA = subgenomic ribonucleic acid; vp = virus particles.

Supplementary Figure 4: Viral load kinetics in the nose of individual animals of the 6M durability study

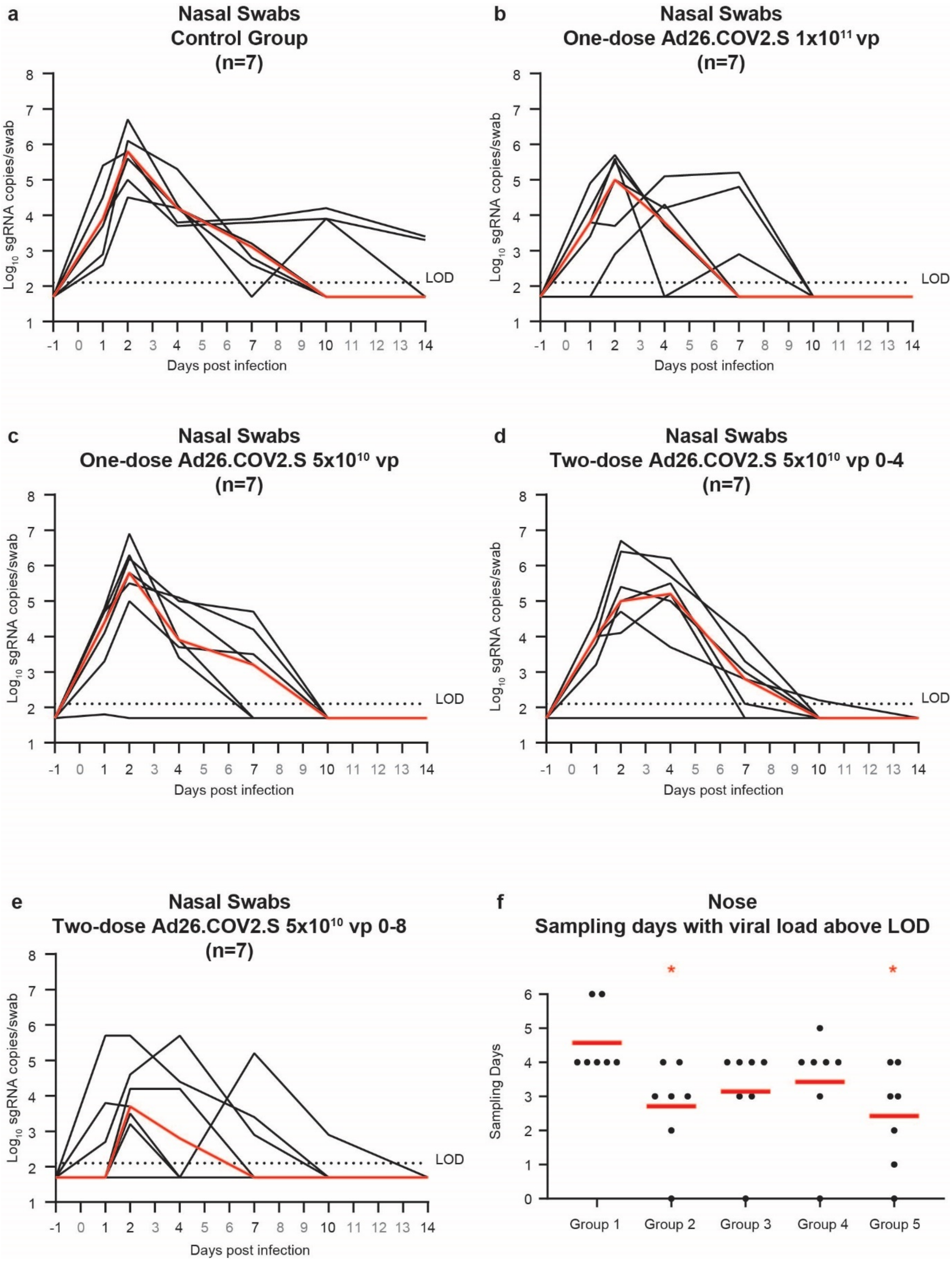

(**a**-**e**) Six months (25 weeks) post immunization the NHPs were inoculated with SARS-CoV-2. Viral load was analyzed in nasal swabs taken from NHPs immunized with a single dose of Ad26.COV2.S at a dose level of $1x10^{11}$ vp (N=7; Group 2; panel **b**), a single dose of Ad26.COV2.S at a dose level of $5x10^{10}$ vp (N=7; Group 3; panel **c**), two doses of Ad26.COV2.S at a dose level of $5x10^{10}$ vp with a 4-week interval (N=7; Group 4; panel **d**), two doses of Ad26.COV2.S at a dose level of $5x10^{10}$ vp with an 8-week interval (N=7; Group 5; panel **e**) or control animals (N=4 immunized with saline, N=3 treatment naïve; Group 1; panel **a**) at Day -1, 1, 2, 4, 7, 10 and at sacrifice (Day 13 or 14 depending on animal cohort assignment) after SARS-CoV-2 challenge. In these graphs viral load values observed at sacrifice are shown at Day 14 for all animals. RNA was extracted from nasal swabs, followed by RT-qPCR using sequences targeting the E gene sgRNA of virus replication cellular intermediates. LOD is set at 125 copies/mL (Log_{10} = 2.1) based on the limit of detection being 1 RNA copy per reaction multiplied by 125 to correct for sample dilutions. The median viral load per group is indicated in red, viral load in individual animals is indicated in black. On the x axis, days post infections on which samples were collected are shown in black; days post infections on which samples were not collected as per study plan are shown in grey. **(f)** Duration (days) of viral load in nasal swabs after SARS-CoV-2 challenged of control and vaccinated macaques. Black dots represent individual animals, red lines the group mean. Data represent results from a single study, and were obtained as technical duplicates. The statistical analysis was performed using a two-sided Mann-Whitney U test. Red asterisks indicate the statistical significance. (*p<0.05, ** p<0.01, *** p<0.001). Exact p-values for panel **f** are 0.0122 for Group 2, 0.0734 for Group 3, 0.196 for Group 4 and 0.0122 for Group 5.

LOD = Limit of detection; LRT = lower respiratory track; N = number of animals; NHPs = nonhuman primates; RNA = ribonucleic acid; RT-qPCR = reverse transcriptase quantitative polymerase chain reaction; sgRNA = subgenomic ribonucleic acid; vp = virus particles.

Supplementary Figure 5: Comparison between observed and predicted protection based on correlate models obtained with data of all vaccine candidates combined.

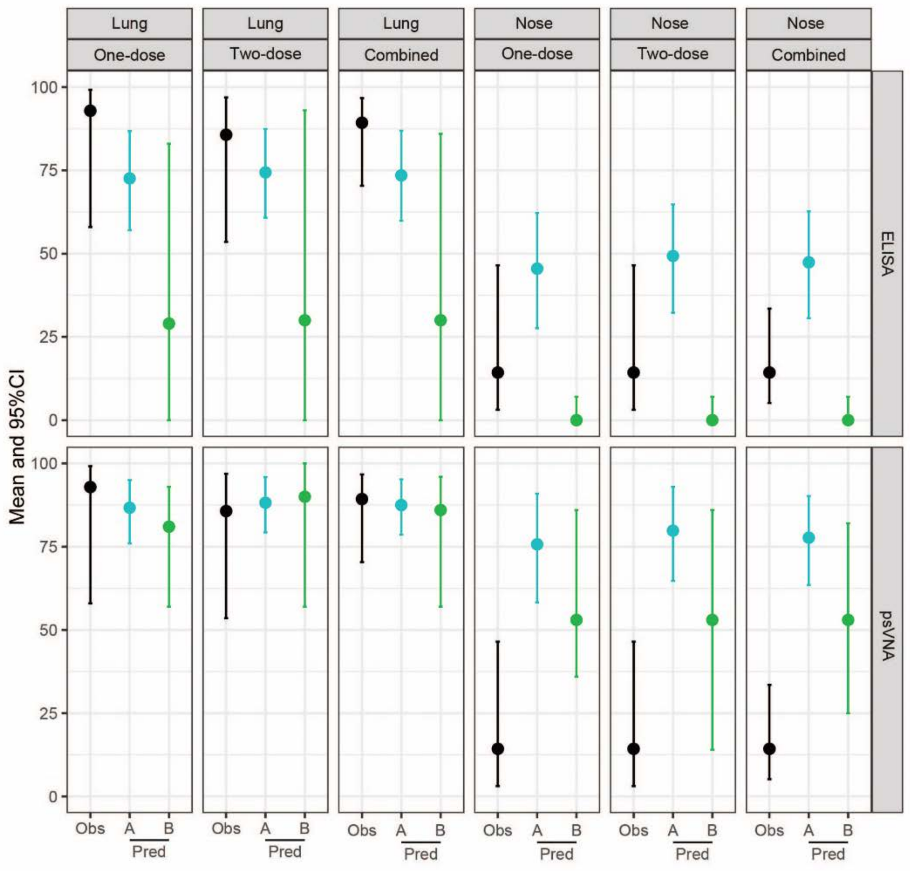

Comparison of the observed (Obs, One-Dose n=14, Two-Dose n=14, Combined n=28) protection probability 6 months after vaccination with Ad26.COV2.S with the predicted (Pred) protection probability based on pre-challenge binding (S-ELISA) and neutralizing antibody (psVNA) levels and correlate of protection models (logistic models (Pred A) and sigmoid Emax models (Pred B)) constructed based on immunogenicity data of all Ad26-based vaccine candidates, obtained 4 weeks after vaccination in a total of 81 NHP from 3-independent studies. Dots are the mean values and the vertical lines represent the 95% CI. Predictions are shown for both models based on data of all vaccine candidates in both the lung and the nose.

SUPPLEMENTARY TABLES

Supplementary Table 1: Schematic representation of the three NHP studies used to build the models

Study	Immunization (Week 0)	Construct	Dose (vp)	SARS-CoV-2 Challenge (Week 6)	Number of NHP
20-07	Ad26NCOV002	tPA.S	$1x10^{11}$	$1x10^5$ $TCID_{50}$ USA-WA1/2020	4
	Ad26NCOV004	tPA.S.PP	$1x10^{11}$		4
	Ad26NCOV006	S	$1x10^{11}$		4
	Ad26NCOV008	S.dCT	$1x10^{11}$		4
	Ad26NCOV014	tPA.WT.S	$1x10^{11}$		4
	Ad26NCOV028	S.dTM.PP	$1x10^{11}$		4
20-09	Ad26NCOV028	S.dTM.PP	$1x10^{11}$		2
	Ad26.COV2.S (Ad26NCOV030)	S.PP	$1x10^{11}$		6
20-14	Ad26.COV2.S (Ad26NCOV030)	S.PP	$1x10^{11}$		5
			$5x10^{10}$		5
			$1.125x10^{10}$		5
			$2x10^9$		5
20-07	N/A	N/A	N/A		10
20-09	N/A	N/A	N/A		10
20-14	N/A	N/A	N/A		10

Vp: viral particles; NHP: nonhuman primates; tPA.S: full length spike protein (S) in which the wild-type (wt) signal peptide (SP) is replaced by tissue plasminogen activator (tPA) SP; tPA.S.PP: full length S in which SP is replaced by tPA and in which the furin cleavage site mutations and proline substitutions (K986P, V987P) have been introduced; S: native full length spike protein; S.dCT: full length S in which the cytoplasmic tail (CT) is deleted; tPA.WT.S: full-length S in which tPA SP is added upstream of the wt SP; S.dTM.PP: full length S in which the transmembrane domain (TM) is deleted and the furin cleavage site mutations and proline substitutions (K986P, V987P) have been introduced; S.PP: full length S in which furin cleavage site mutations and proline substitutions have been introduced; $TCID_{50}$: 50% Tissue Culture Infectious Dose; N/A: not applicable, which refer to the placebo animal group.

Supplementary Table 2: Six-month durability study design

**Group**	**N**	**Treatment**	**Dose 1 (vp)**	**Dose 2 (vp)**	**Interval (Weeks)**	**SARS-CoV-2 Inoculation $1x10^5$ $TCID_{50}$ USA-WA1/2020 (Week^)**
1	7	Control (Placebo)[a]	Saline	Saline	8/-	25
2	7	Single dose of Ad26.COV2.S	$1 x 10^{11}$	N/A	0	25
3	7	Single dose of Ad26.COV2.S	$5 x 10^{10}$	N/A	0	25
4	7	Two doses of Ad26.COV2.S	$5 x 10^{10}$	$5 x 10^{10}$	4	25
5	7	Two doses of Ad26.COV2.S	$5 x 10^{10}$	$5 x 10^{10}$	8	25

[a]In the control group, four animals in the control group received saline whereas three were added to the control group after immunization and did not receive saline. ^Week number refers to the total number of weeks that passed between the first day of immunization (Ad26.COV2.S or saline) and the SARS-CoV-2 challenge day. Week 0 is considered the week of the first immunization.
N/A: not applicable; PFU: plaque forming units; $TCID_{50}$: 50% Tissue Culture Infectious Dose; vp: viral particles.

Supplementary Table 3: Statistical comparison between vaccinated vs control groups in the six-month durability study

**Lung**		
**Ad26.COV2.S dose level and regimen**	**Peak viral load**	**Number of sampling days with viral load above LOD**
$1x10^{11}$ vp	0.000583 ***	0.000583 ***
$5x10^{10}$ vp	0.00116 **	0.00175 **
$5x10^{10}$ vp Week 0,4	0.000583 ***	0.000583 ***
$5x10^{10}$ vp Week 0,8	0.00350 **	0.00641**
**Nose**		
**Ad26.COV2.S dose level and regimen**	**Peak viral load**	**Number of sampling days with viral load above LOD**
$1x10^{11}$ vp	0.0730	0.0122 *
$5x10^{10}$ vp	1.000	0.0734
$5x10^{10}$ vp Week 0,4	0.535	0.196
$5x10^{10}$ vp Week 0,8	0.0379 *	0.0122 *

Comparison of peak viral load and number of sampling days with viral load above lower limit of detection (LOD) of lung and nose samples taken at Day 13 after SARS-CoV-2 inoculation. Pairwise comparison of nonhuman primates (NHPs) immunized with different Ad26.COV2.S dose levels and regimens versus NHPs included in the control group was done with a 2-sided Mann-Whitney test. Significant difference is indicated by asterisks: ***: $p \leq 0.001$; **: $p \leq 0.01$; *: $p \leq 0.05$.

Supplementary Table 4: Source data file for Figure 1

Panel a and b

Animal ID	Study #	Vaccine construct	Lung peak viral load (copies/mL)	psVNA (log_{10} IC_{50})	Protection_lung
6418	20-07	Sham	4.543	1.415	0
6768	20-07	S.dCT	≤LOD	1.987	1
6770	20-07	tPA.WT.S	≤LOD	2.079	1
6855	20-07	tPA.S	≤LOD	1.892	1
6856	20-07	tPA.S	5.097	1.613	0
6905	20-07	S.dTM.PP	≤LOD	2.225	1
6906	20-07	S.dTM.PP	3.812	1.908	0
7028	20-07	tPA.S.PP	3.341	2.083	0
7056	20-07	tPA.S.PP	4.641	1.892	0
7123	20-07	Sham	5.575	NA	0
7130	20-07	Sham	4.895	NA	0
7145	20-07	Sham	6.103	NA	0
7154	20-07	Sham	4.615	NA	0
7157	20-07	Sham	4.871	NA	0
7245	20-07	Sham	6.288	NA	0
7261	20-07	Sham	5.400	NA	0
BM39	20-07	tPA.S	4.170	1.505	0
BO49	20-07	tPA.S	≤LOD	1.732	1
BO93	20-07	tPA.S.PP	≤LOD	2.236	1
BO95	20-07	tPA.S.PP	≤LOD	2.072	1
BP11	20-07	S.dCT	3.514	2.100	0
BS64	20-07	Sham	4.036	1.491	0
BS80	20-07	Sham	4.927	1.398	0
BS82	20-07	S	3.636	2.009	0
BV16	20-07	S	≤LOD	2.017	1
BV35	20-07	S.dCT	≤LOD	1.987	1
BV36	20-07	S.dCT	≤LOD	2.037	1
BV58	20-07	tPA.WT.S	≤LOD	2.017	1
BV79	20-07	tPA.WT.S	≤LOD	2.312	1
BV95	20-07	S.dTM.PP	≤LOD	2.111	1
BV96	20-07	S.dTM.PP	3.070	1.724	0
T781	20-07	S	3.733	2.201	0
T796	20-07	S	≤LOD	2.305	1
T799	20-07	tPA.WT.S	≤LOD	1.748	1
171170	20-09	S.dTM.PP	≤LOD	1.716	1
171219	20-09	Sham	4.934	1.505	0
171264	20-09	Sham	3.958	1.301	0
180662	20-09	S.PP	≤LOD	2.751	1
180689	20-09	S.PP	≤LOD	2.318	1
181267	20-09	S.PP	≤LOD	2.637	1
181302	20-09	S.PP	≤LOD	2.808	1

Animal ID	Study #	Vaccine construct	Lung peak viral load (copies/mL)	psVNA (log_{10} IC_{50})	Protection_lung
181343	20-09	S.PP	≤LOD	2.581	1
181363	20-09	S.PP	≤LOD	2.525	1
191358	20-09	Sham	5.912	1.519	0
191372	20-09	Sham	4.443	1.462	0
T414	20-09	Sham	3.846	NA	0
T423	20-09	Sham	3.587	NA	0
T436	20-09	Sham	6.510	NA	0
T440	20-09	Sham	5.316	NA	0
T457	20-09	Sham	4.614	NA	0
T463	20-09	Sham	4.592	NA	0
15-072	20-14	S.PP	≤LOD	2.369	1
15-084	20-14	S.PP	≤LOD	2.840	1
15-086	20-14	S.PP	≤LOD	2.643	1
15-104	20-14	S.PP	≤LOD	2.061	1
15-108	20-14	S.PP	≤LOD	1.886	1
15-114	20-14	S.PP	2.623	1.973	0
15-115	20-14	S.PP	≤LOD	2.173	1
15-117	20-14	S.PP	≤LOD	2.910	1
15-118	20-14	S.PP	≤LOD	1.301	1
15-131	20-14	S.PP	≤LOD	2.455	1
15-139	20-14	S.PP	≤LOD	2.519	1
15-140	20-14	S.PP	3.962	1.301	0
15-143	20-14	S.PP	≤LOD	2.267	1
15-145	20-14	S.PP	≤LOD	2.840	1
15-146	20-14	Sham	4.691	1.301	0
15-148	20-14	Sham	3.164	1.301	0
15-157	20-14	S.PP	≤LOD	2.456	1
15-159	20-14	S.PP	≤LOD	2.910	1
15-161	20-14	S.PP	≤LOD	2.146	1
15-169	20-14	S.PP	≤LOD	1.301	1
15-171	20-14	S.PP	≤LOD	2.053	1
15-173	20-14	S.PP	≤LOD	1.699	1
15-175	20-14	Sham	5.064	1.301	0
15-179	20-14	Sham	3.877	1.301	0
15-183	20-14	Sham	3.888	1.301	0
T434F	20-14	Sham	2.696	NA	0
T435F	20-14	Sham	4.277	NA	0
T455M	20-14	Sham	3.905	NA	0
T458M	20-14	Sham	6.487	NA	0
T459M	20-14	Sham	4.182	NA	0

psVNA: pseudotyped virus neutralization assay; IC_{50}: 50% neutralization antibody titer; LOD: limit of detection; NA: not available

Panel c and d

Animal ID	Study #	Vaccine construct	Lung peak viral load (copies/mL)	ELISA (log_{10} EU/ml)	Protection_lung
6418	20-07	Sham	4.543	1.702	0
6768	20-07	S.dCT	≤LOD	NA	1
6770	20-07	tPA.WT.S	≤LOD	NA	1
6855	20-07	tPA.S	≤LOD	2.479	1
6856	20-07	tPA.S	5.097	2.163	0
6905	20-07	S.dTM.PP	≤LOD	3.279	1
6906	20-07	S.dTM.PP	3.812	2.515	0
7028	20-07	tPA.S.PP	3.341	3.452	0
7056	20-07	tPA.S.PP	4.641	3.142	0
7123	20-07	Sham	5.575	NA	0
7130	20-07	Sham	4.895	NA	0
7145	20-07	Sham	6.103	NA	0
7154	20-07	Sham	4.615	NA	0
7157	20-07	Sham	4.871	NA	0
7245	20-07	Sham	6.288	NA	0
7261	20-07	Sham	5.400	NA	0
BM39	20-07	tPA.S	4.170	1.702	0
BO49	20-07	tPA.S	≤LOD	2.357	1
BO93	20-07	tPA.S.PP	≤LOD	3.130	1
BO95	20-07	tPA.S.PP	≤LOD	3.132	1
BP11	20-07	S.dCT	3.514	3.185	0
BS64	20-07	Sham	4.036	1.702	0
BS80	20-07	Sham	4.927	3.752	0
BS82	20-07	S	3.636	3.290	0
BV16	20-07	S	≤LOD	3.500	1
BV35	20-07	S.dCT	≤LOD	2.637	1
BV36	20-07	S.dCT	≤LOD	3.293	1
BV58	20-07	tPA.WT.S	≤LOD	3.662	1
BV79	20-07	tPA.WT.S	≤LOD	3.914	1
BV95	20-07	S.dTM.PP	≤LOD	4.288	1
BV96	20-07	S.dTM.PP	3.070	4.209	0
T781	20-07	S	3.733	2.822	0
T796	20-07	S	≤LOD	3.174	1
T799	20-07	tPA.WT.S	≤LOD	2.724	1
171170	20-09	S.dTM.PP	≤LOD	3.060	1
171219	20-09	Sham	4.934	1.702	0
171264	20-09	Sham	3.958	1.702	0
180662	20-09	S.PP	≤LOD	3.615	1
180689	20-09	S.PP	≤LOD	3.149	1
181267	20-09	S.PP	≤LOD	3.455	1
181302	20-09	S.PP	≤LOD	3.710	1
181343	20-09	S.PP	≤LOD	3.198	1
181363	20-09	S.PP	≤LOD	3.097	1

Animal ID	Study #	Vaccine construct	Lung peak viral load (copies/mL)	ELISA (log_{10} EU/ml)	Protection_lung
191358	20-09	Sham	5.912	1.702	0
191372	20-09	Sham	4.443	1.702	0
T414	20-09	Sham	3.846	NA	0
T423	20-09	Sham	3.587	NA	0
T436	20-09	Sham	6.510	NA	0
T440	20-09	Sham	5.316	NA	0
T457	20-09	Sham	4.614	NA	0
T463	20-09	Sham	4.592	NA	0
15-072	20-14	S.PP	≤LOD	3.407	1
15-084	20-14	S.PP	≤LOD	3.687	1
15-086	20-14	S.PP	≤LOD	3.567	1
15-104	20-14	S.PP	≤LOD	2.785	1
15-108	20-14	S.PP	≤LOD	2.759	1
15-114	20-14	S.PP	2.623	2.746	0
15-115	20-14	S.PP	≤LOD	3.042	1
15-117	20-14	S.PP	≤LOD	3.507	1
15-118	20-14	S.PP	≤LOD	2.073	1
15-131	20-14	S.PP	≤LOD	3.277	1
15-139	20-14	S.PP	≤LOD	3.368	1
15-140	20-14	S.PP	3.962	1.702	0
15-143	20-14	S.PP	≤LOD	3.068	1
15-145	20-14	S.PP	≤LOD	3.041	1
15-146	20-14	Sham	4.691	1.702	0
15-148	20-14	Sham	3.164	1.702	0
15-157	20-14	S.PP	≤LOD	3.308	1
15-159	20-14	S.PP	≤LOD	3.496	1
15-161	20-14	S.PP	≤LOD	2.956	1
15-169	20-14	S.PP	≤LOD	1.702	1
15-171	20-14	S.PP	≤LOD	2.617	1
15-173	20-14	S.PP	≤LOD	1.958	1
15-175	20-14	Sham	5.064	1.702	0
15-179	20-14	Sham	3.877	1.702	0
15-183	20-14	Sham	3.888	1.702	0
T434F	20-14	Sham	2.696	NA	0
T435F	20-14	Sham	4.277	NA	0
T455M	20-14	Sham	3.905	NA	0
T458M	20-14	Sham	6.487	NA	0
T459M	20-14	Sham	4.182	NA	0

ELISA: enzyme-linked immunosorbent assay; EU/mL: ELISA unit per milliliter; LOD: limit of detection; NA: not available

Panel e and f

Animal ID	Study #	Vaccine construct	Nose peak viral load (copies/swab)	psVNA (log_{10} IC_{50})	Protection_nose
6418	20-07	Sham	6.209	1.415	0
6768	20-07	S.dCT	≤LOD	1.987	1
6770	20-07	tPA.WT.S	6.162	2.079	0
6855	20-07	tPA.S	≤LOD	1.892	1
6856	20-07	tPA.S	5.087	1.613	0
6905	20-07	S.dTM.PP	≤LOD	2.225	1
6906	20-07	S.dTM.PP	6.104	1.908	0
7028	20-07	tPA.S.PP	5.965	2.083	0
7056	20-07	tPA.S.PP	5.701	1.892	0
7123	20-07	Sham	6.345	NA	0
7130	20-07	Sham	5.386	NA	0
7145	20-07	Sham	5.632	NA	0
7154	20-07	Sham	NA	NA	NA
7157	20-07	Sham	5.958	NA	0
7245	20-07	Sham	4.726	NA	0
7261	20-07	Sham	6.089	NA	0
BM39	20-07	tPA.S	6.513	1.505	0
BO49	20-07	tPA.S	≤LOD	1.732	1
BO93	20-07	tPA.S.PP	≤LOD	2.236	1
BO95	20-07	tPA.S.PP	5.031	2.072	0
BP11	20-07	S.dCT	5.930	2.100	0
BS64	20-07	Sham	4.485	1.491	0
BS80	20-07	Sham	5.111	1.398	0
BS82	20-07	S	5.078	2.009	0
BV16	20-07	S	≤LOD	2.017	1
BV35	20-07	S.dCT	≤LOD	1.987	1
BV36	20-07	S.dCT	≤LOD	2.037	1
BV58	20-07	tPA.WT.S	5.620	2.017	0
BV79	20-07	tPA.WT.S	≤LOD	2.312	1
BV95	20-07	S.dTM.PP	≤LOD	2.111	1
BV96	20-07	S.dTM.PP	5.785	1.724	0
T781	20-07	S	6.220	2.201	0
T796	20-07	S	≤LOD	2.305	1
T799	20-07	tPA.WT.S	5.951	1.748	0
171170	20-09	S.dTM.PP	3.858	1.716	0
171219	20-09	Sham	6.342	1.505	0
171264	20-09	Sham	6.705	1.301	0
180662	20-09	S.PP	≤LOD	2.751	1
180689	20-09	S.PP	≤LOD	2.318	1
181267	20-09	S.PP	≤LOD	2.637	1
181302	20-09	S.PP	≤LOD	2.808	1
181343	20-09	S.PP	≤LOD	2.581	1
181363	20-09	S.PP	3.604	2.525	0

Animal ID	Study #	Vaccine construct	Nose peak viral load (copies/swab)	psVNA (log_{10} IC_{50})	Protection_nose
191358	20-09	Sham	8.010	1.519	0
191372	20-09	Sham	6.838	1.462	0
T414	20-09	Sham	4.989	NA	0
T423	20-09	Sham	5.199	NA	0
T436	20-09	Sham	5.587	NA	0
T440	20-09	Sham	4.073	NA	0
T457	20-09	Sham	3.781	NA	0
T463	20-09	Sham	5.709	NA	0
15-072	20-14	S.PP	≤LOD	2.369	1
15-084	20-14	S.PP	≤LOD	2.840	1
15-086	20-14	S.PP	≤LOD	2.643	1
15-104	20-14	S.PP	4.265	2.061	0
15-108	20-14	S.PP	4.981	1.886	0
15-114	20-14	S.PP	3.639	1.973	0
15-115	20-14	S.PP	≤LOD	2.173	1
15-117	20-14	S.PP	≤LOD	2.910	1
15-118	20-14	S.PP	5.460	1.301	0
15-131	20-14	S.PP	≤LOD	2.455	1
15-139	20-14	S.PP	≤LOD	2.519	1
15-140	20-14	S.PP	5.569	1.301	0
15-143	20-14	S.PP	≤LOD	2.267	1
15-145	20-14	S.PP	≤LOD	2.840	1
15-146	20-14	Sham	6.154	1.301	0
15-148	20-14	Sham	6.895	1.301	0
15-157	20-14	S.PP	≤LOD	2.456	1
15-159	20-14	S.PP	≤LOD	2.910	1
15-161	20-14	S.PP	≤LOD	2.146	1
15-169	20-14	S.PP	6.187	1.301	0
15-171	20-14	S.PP	≤LOD	2.053	1
15-173	20-14	S.PP	4.763	1.699	0
15-175	20-14	Sham	5.786	1.301	0
15-179	20-14	Sham	3.215	1.301	0
15-183	20-14	Sham	6.093	1.301	0
T434F	20-14	Sham	5.096	NA	0
T435F	20-14	Sham	5.926	NA	0
T455M	20-14	Sham	5.239	NA	0
T458M	20-14	Sham	5.324	NA	0
T459M	20-14	Sham	6.465	NA	0

psVNA: pseudotyped virus neutralization assay; IC_{50}: 50% neutralization antibody titer; LOD: limit of detection; NA: not available

Panel g and h

Animal ID	Study #	Vaccine construct	Nose peak viral load (copies/swab)	ELISA (log_{10} EU/ml)	Protection_nose
6418	20-07	Sham	6.209	1.702	0
6768	20-07	S.dCT	≤LOD	NA	1
6770	20-07	tPA.WT.S	6.162	NA	0
6855	20-07	tPA.S	≤LOD	2.479	1
6856	20-07	tPA.S	5.087	2.163	0
6905	20-07	S.dTM.PP	≤LOD	3.279	1
6906	20-07	S.dTM.PP	6.104	2.515	0
7028	20-07	tPA.S.PP	5.965	3.452	0
7056	20-07	tPA.S.PP	5.701	3.142	0
7123	20-07	Sham	6.345	NA	0
7130	20-07	Sham	5.386	NA	0
7145	20-07	Sham	5.632	NA	0
7154	20-07	Sham	NA	NA	NA
7157	20-07	Sham	5.958	NA	0
7245	20-07	Sham	4.726	NA	0
7261	20-07	Sham	6.089	NA	0
BM39	20-07	tPA.S	6.513	1.702	0
BO49	20-07	tPA.S	≤LOD	2.357	1
BO93	20-07	tPA.S.PP	≤LOD	3.130	1
BO95	20-07	tPA.S.PP	5.031	3.132	0
BP11	20-07	S.dCT	5.930	3.185	0
BS64	20-07	Sham	4.485	1.702	0
BS80	20-07	Sham	5.111	3.752	0
BS82	20-07	S	5.078	3.290	0
BV16	20-07	S	≤LOD	3.500	1
BV35	20-07	S.dCT	≤LOD	2.637	1
BV36	20-07	S.dCT	≤LOD	3.293	1
BV58	20-07	tPA.WT.S	5.620	3.662	0
BV79	20-07	tPA.WT.S	≤LOD	3.914	1
BV95	20-07	S.dTM.PP	≤LOD	4.288	1
BV96	20-07	S.dTM.PP	5.785	4.209	0
T781	20-07	S	6.220	2.822	0
T796	20-07	S	≤LOD	3.174	1
T799	20-07	tPA.WT.S	5.951	2.724	0
171170	20-09	S.dTM.PP	3.858	3.060	0
171219	20-09	Sham	6.342	1.702	0
171264	20-09	Sham	6.705	1.702	0
180662	20-09	S.PP	≤LOD	3.615	1
180689	20-09	S.PP	≤LOD	3.149	1
181267	20-09	S.PP	≤LOD	3.455	1
181302	20-09	S.PP	≤LOD	3.710	1
181343	20-09	S.PP	≤LOD	3.198	1
181363	20-09	S.PP	3.604	3.097	0

Animal ID	Study #	Vaccine construct	Nose peak viral load (copies/swab)	ELISA (log_{10} EU/ml)	Protection_nose
191358	20-09	Sham	8.010	1.702	0
191372	20-09	Sham	6.838	1.702	0
T414	20-09	Sham	4.989	NA	0
T423	20-09	Sham	5.199	NA	0
T436	20-09	Sham	5.587	NA	0
T440	20-09	Sham	4.073	NA	0
T457	20-09	Sham	3.781	NA	0
T463	20-09	Sham	5.709	NA	0
15-072	20-14	S.PP	≤LOD	3.407	1
15-084	20-14	S.PP	≤LOD	3.687	1
15-086	20-14	S.PP	≤LOD	3.567	1
15-104	20-14	S.PP	4.265	2.785	0
15-108	20-14	S.PP	4.981	2.759	0
15-114	20-14	S.PP	3.639	2.746	0
15-115	20-14	S.PP	≤LOD	3.042	1
15-117	20-14	S.PP	≤LOD	3.507	1
15-118	20-14	S.PP	5.460	2.073	0
15-131	20-14	S.PP	≤LOD	3.277	1
15-139	20-14	S.PP	≤LOD	3.368	1
15-140	20-14	S.PP	5.569	1.702	0
15-143	20-14	S.PP	≤LOD	3.068	1
15-145	20-14	S.PP	≤LOD	3.041	1
15-146	20-14	Sham	6.154	1.702	0
15-148	20-14	Sham	6.895	1.702	0
15-157	20-14	S.PP	≤LOD	3.308	1
15-159	20-14	S.PP	≤LOD	3.496	1
15-161	20-14	S.PP	≤LOD	2.956	1
15-169	20-14	S.PP	6.187	1.702	0
15-171	20-14	S.PP	≤LOD	2.617	1
15-173	20-14	S.PP	4.763	1.958	0
15-175	20-14	Sham	5.786	1.702	0
15-179	20-14	Sham	3.215	1.702	0
15-183	20-14	Sham	6.093	1.702	0
T434F	20-14	Sham	5.096	NA	0
T435F	20-14	Sham	5.926	NA	0
T455M	20-14	Sham	5.239	NA	0
T458M	20-14	Sham	5.324	NA	0
T459M	20-14	Sham	6.465	NA	0

ELISA: enzyme-linked immunosorbent assay; EU/mL: ELISA unit per milliliter; LOD: limit of detection; NA: not available

Supplementary Table 5: Source data file for Figure 2

Panel a and b

Animal ID	Study #	Vaccine construct	psVNA (log_{10} IC_{50})	Lung_reduction_R0	Protection_lung
6418	20-07	Sham	1.415	0.001	0
6768	20-07	S.dCT	1.987	0.973	1
6770	20-07	tPA.WT.S	2.079	0.973	1
6855	20-07	tPA.S	1.892	0.973	1
6856	20-07	tPA.S	1.613	0.170	0
6905	20-07	S.dTM.PP	2.225	0.973	1
6906	20-07	S.dTM.PP	1.908	0.172	0
7028	20-07	tPA.S.PP	2.083	0.002	0
7056	20-07	tPA.S.PP	1.892	0.001	0
7123	20-07	Sham	NA	0.262	0
7130	20-07	Sham	NA	0.297	0
7145	20-07	Sham	NA	0.168	0
7154	20-07	Sham	NA	0.122	0
7157	20-07	Sham	NA	0.001	0
7245	20-07	Sham	NA	0.001	0
7261	20-07	Sham	NA	0.001	0
BM39	20-07	tPA.S	1.505	0.002	0
BO49	20-07	tPA.S	1.732	0.973	1
BO93	20-07	tPA.S.PP	2.236	0.973	1
BO95	20-07	tPA.S.PP	2.072	0.973	1
BP11	20-07	S.dCT	2.100	0.214	0
BS64	20-07	Sham	1.491	0.002	0
BS80	20-07	Sham	1.398	0.001	0
BS82	20-07	S	2.009	0.645	0
BV16	20-07	S	2.017	0.973	1
BV35	20-07	S.dCT	1.987	0.973	1
BV36	20-07	S.dCT	2.037	0.973	1
BV58	20-07	tPA.WT.S	2.017	0.973	1
BV79	20-07	tPA.WT.S	2.312	0.973	1
BV95	20-07	S.dTM.PP	2.111	0.973	1
BV96	20-07	S.dTM.PP	1.724	0.002	0
T781	20-07	S	2.201	0.183	0
T796	20-07	S	2.305	0.973	1
T799	20-07	tPA.WT.S	1.748	0.973	1
171170	20-09	S.dTM.PP	1.716	0.973	1
171219	20-09	Sham	1.505	0.001	0
171264	20-09	Sham	1.301	0.001	0
180662	20-09	S.PP	2.751	0.973	1

Animal ID	Study #	Vaccine construct	psVNA (log_{10} IC_{50})	Lung_reduction_R0	Protection_lung
180689	20-09	S.PP	2.318	0.973	1
181267	20-09	S.PP	2.637	0.973	1
181302	20-09	S.PP	2.808	0.973	1
181343	20-09	S.PP	2.581	0.973	1
181363	20-09	S.PP	2.525	0.973	1
191358	20-09	Sham	1.519	0.001	0
191372	20-09	Sham	1.462	0.001	0
T414	20-09	Sham	NA	0.481	0
T423	20-09	Sham	NA	0.001	0
T436	20-09	Sham	NA	0.000	0
T440	20-09	Sham	NA	0.001	0
T457	20-09	Sham	NA	0.001	0
T463	20-09	Sham	NA	0.001	0
15-072	20-14	S.PP	2.369	0.973	1
15-084	20-14	S.PP	2.840	0.973	1
15-086	20-14	S.PP	2.643	0.973	1
15-104	20-14	S.PP	2.061	0.973	1
15-108	20-14	S.PP	1.886	0.973	1
15-114	20-14	S.PP	1.973	0.001	0
15-115	20-14	S.PP	2.173	0.973	1
15-117	20-14	S.PP	2.910	0.973	1
15-118	20-14	S.PP	1.301	0.973	1
15-131	20-14	S.PP	2.455	0.973	1
15-139	20-14	S.PP	2.519	0.973	1
15-140	20-14	S.PP	1.301	0.152	0
15-143	20-14	S.PP	2.267	0.973	1
15-145	20-14	S.PP	2.840	0.973	1
15-146	20-14	Sham	1.301	0.002	0
15-148	20-14	Sham	1.301	0.547	0
15-157	20-14	S.PP	2.456	0.973	1
15-159	20-14	S.PP	2.910	0.973	1
15-161	20-14	S.PP	2.146	0.973	1
15-169	20-14	S.PP	1.301	0.973	1
15-171	20-14	S.PP	2.053	0.973	1
15-173	20-14	S.PP	1.699	0.973	1
15-175	20-14	Sham	1.301	0.001	0
15-179	20-14	Sham	1.301	0.499	0
15-183	20-14	Sham	1.301	0.194	0
T434F	20-14	Sham	NA	0.000	0
T435F	20-14	Sham	NA	0.001	0
T455M	20-14	Sham	NA	0.568	0

**Animal ID**	**Study #**	**Vaccine construct**	**psVNA (log_{10} IC_{50})**	**Lung_reduction_R0**	**Protection_lung**
T458M	20-14	Sham	NA	0.000	0
T459M	20-14	Sham	NA	0.001	0

psVNA: pseudotyped virus neutralization assay; IC_{50}: 50% neutralization antibody titer; R0: virus basic reproductive ratio; NA: not available

Panel c and d

Animal ID	Study #	Vaccine construct	ELISA (log_{10} EU/ml)	Lung_reduction_R0	Protection_lung
6418	20-07	Sham	1.702	0.001	0
6768	20-07	S.dCT	NA	0.973	1
6770	20-07	tPA.WT.S	NA	0.973	1
6855	20-07	tPA.S	2.479	0.973	1
6856	20-07	tPA.S	2.163	0.170	0
6905	20-07	S.dTM.PP	3.279	0.973	1
6906	20-07	S.dTM.PP	2.515	0.172	0
7028	20-07	tPA.S.PP	3.452	0.002	0
7056	20-07	tPA.S.PP	3.142	0.001	0
7123	20-07	Sham	NA	0.262	0
7130	20-07	Sham	NA	0.297	0
7145	20-07	Sham	NA	0.168	0
7154	20-07	Sham	NA	0.122	0
7157	20-07	Sham	NA	0.001	0
7245	20-07	Sham	NA	0.001	0
7261	20-07	Sham	NA	0.001	0
BM39	20-07	tPA.S	1.702	0.002	0
BO49	20-07	tPA.S	2.357	0.973	1
BO93	20-07	tPA.S.PP	3.130	0.973	1
BO95	20-07	tPA.S.PP	3.132	0.973	1
BP11	20-07	S.dCT	3.185	0.214	0
BS64	20-07	Sham	1.702	0.002	0
BS80	20-07	Sham	3.752	0.001	0
BS82	20-07	S	3.290	0.645	0
BV16	20-07	S	3.500	0.973	1
BV35	20-07	S.dCT	2.637	0.973	1
BV36	20-07	S.dCT	3.293	0.973	1
BV58	20-07	tPA.WT.S	3.662	0.973	1
BV79	20-07	tPA.WT.S	3.914	0.973	1
BV95	20-07	S.dTM.PP	4.288	0.973	1
BV96	20-07	S.dTM.PP	4.209	0.002	0
T781	20-07	S	2.822	0.183	0
T796	20-07	S	3.174	0.973	1
T799	20-07	tPA.WT.S	2.724	0.973	1
171170	20-09	S.dTM.PP	3.060	0.973	1
171219	20-09	Sham	1.702	0.001	0
171264	20-09	Sham	1.702	0.001	0
180662	20-09	S.PP	3.615	0.973	1
180689	20-09	S.PP	3.149	0.973	1
181267	20-09	S.PP	3.455	0.973	1
181302	20-09	S.PP	3.710	0.973	1
181343	20-09	S.PP	3.198	0.973	1
181363	20-09	S.PP	3.097	0.973	1

Animal ID	Study #	Vaccine construct	ELISA (log_{10} EU/ml)	Lung_reduction_R0	Protection_lung
191358	20-09	Sham	1.702	0.001	0
191372	20-09	Sham	1.702	0.001	0
T414	20-09	Sham	NA	0.481	0
T423	20-09	Sham	NA	0.001	0
T436	20-09	Sham	NA	0.000	0
T440	20-09	Sham	NA	0.001	0
T457	20-09	Sham	NA	0.001	0
T463	20-09	Sham	NA	0.001	0
15-072	20-14	S.PP	3.407	0.973	1
15-084	20-14	S.PP	3.687	0.973	1
15-086	20-14	S.PP	3.567	0.973	1
15-104	20-14	S.PP	2.785	0.973	1
15-108	20-14	S.PP	2.759	0.973	1
15-114	20-14	S.PP	2.746	0.001	0
15-115	20-14	S.PP	3.042	0.973	1
15-117	20-14	S.PP	3.507	0.973	1
15-118	20-14	S.PP	2.073	0.973	1
15-131	20-14	S.PP	3.277	0.973	1
15-139	20-14	S.PP	3.368	0.973	1
15-140	20-14	S.PP	1.702	0.152	0
15-143	20-14	S.PP	3.068	0.973	1
15-145	20-14	S.PP	3.041	0.973	1
15-146	20-14	Sham	1.702	0.002	0
15-148	20-14	Sham	1.702	0.547	0
15-157	20-14	S.PP	3.308	0.973	1
15-159	20-14	S.PP	3.496	0.973	1
15-161	20-14	S.PP	2.956	0.973	1
15-169	20-14	S.PP	1.702	0.973	1
15-171	20-14	S.PP	2.617	0.973	1
15-173	20-14	S.PP	1.958	0.973	1
15-175	20-14	Sham	1.702	0.001	0
15-179	20-14	Sham	1.702	0.499	0
15-183	20-14	Sham	1.702	0.194	0
T434F	20-14	Sham	NA	0.000	0
T435F	20-14	Sham	NA	0.001	0
T455M	20-14	Sham	NA	0.568	0
T458M	20-14	Sham	NA	0.000	0
T459M	20-14	Sham	NA	0.001	0

ELISA: enzyme-linked immunosorbent assay; EU/mL: ELISA unit per milliliter; R0: virus basic reproductive ratio; NA: not available

Panel e and f

Animal ID	Study #	Vaccine construct	psVNA (log_{10} IC_{50})	Nose_reduction_R0	Protection_nose
6418	20-07	Sham	1.415	0.000	0
6768	20-07	S.dCT	1.987	0.967	1
6770	20-07	tPA.WT.S	2.079	0.405	0
6855	20-07	tPA.S	1.892	0.967	1
6856	20-07	tPA.S	1.613	0.258	0
6905	20-07	S.dTM.PP	2.225	0.967	1
6906	20-07	S.dTM.PP	1.908	0.255	0
7028	20-07	tPA.S.PP	2.083	0.336	0
7056	20-07	tPA.S.PP	1.892	0.064	0
7123	20-07	Sham	NA	0.000	0
7130	20-07	Sham	NA	0.000	0
7145	20-07	Sham	NA	0.000	0
7154	20-07	Sham	NA	0.000	0
7157	20-07	Sham	NA	0.000	0
7245	20-07	Sham	NA	0.000	0
7261	20-07	Sham	NA	0.000	0
BM39	20-07	tPA.S	1.505	0.069	0
BO49	20-07	tPA.S	1.732	0.967	1
BO93	20-07	tPA.S.PP	2.236	0.967	1
BO95	20-07	tPA.S.PP	2.072	0.023	0
BP11	20-07	S.dCT	2.100	0.060	0
BS64	20-07	Sham	1.491	0.000	0
BS80	20-07	Sham	1.398	0.042	0
BS82	20-07	S	2.009	0.127	0
BV16	20-07	S	2.017	0.967	1
BV35	20-07	S.dCT	1.987	0.967	1
BV36	20-07	S.dCT	2.037	0.967	1
BV58	20-07	tPA.WT.S	2.017	0.489	0
BV79	20-07	tPA.WT.S	2.312	0.967	1
BV95	20-07	S.dTM.PP	2.111	0.967	1
BV96	20-07	S.dTM.PP	1.724	0.064	0
T781	20-07	S	2.201	0.331	0
T796	20-07	S	2.305	0.967	1
T799	20-07	tPA.WT.S	1.748	0.063	0
171170	20-09	S.dTM.PP	1.716	0.020	0
171219	20-09	Sham	1.505	0.000	0
171264	20-09	Sham	1.301	0.000	0
180662	20-09	S.PP	2.751	0.967	1
180689	20-09	S.PP	2.318	0.967	1
181267	20-09	S.PP	2.637	0.967	1
181302	20-09	S.PP	2.808	0.967	1
181343	20-09	S.PP	2.581	0.967	1
181363	20-09	S.PP	2.525	0.023	0

Animal ID	Study #	Vaccine construct	psVNA (log_{10} IC_{50})	Nose_reduction_R0	Protection_nose
191358	20-09	Sham	1.519	0.000	0
191372	20-09	Sham	1.462	0.000	0
T414	20-09	Sham	NA	0.000	0
T423	20-09	Sham	NA	0.015	0
T436	20-09	Sham	NA	0.044	0
T440	20-09	Sham	NA	0.099	0
T457	20-09	Sham	NA	0.000	0
T463	20-09	Sham	NA	0.000	0
15-072	20-14	S.PP	2.369	0.967	1
15-084	20-14	S.PP	2.840	0.967	1
15-086	20-14	S.PP	2.643	0.967	1
15-104	20-14	S.PP	2.061	0.026	0
15-108	20-14	S.PP	1.886	0.050	0
15-114	20-14	S.PP	1.973	0.322	0
15-115	20-14	S.PP	2.173	0.967	1
15-117	20-14	S.PP	2.910	0.967	1
15-118	20-14	S.PP	1.301	0.118	0
15-131	20-14	S.PP	2.455	0.967	1
15-139	20-14	S.PP	2.519	0.967	1
15-140	20-14	S.PP	1.301	0.039	0
15-143	20-14	S.PP	2.267	0.967	1
15-145	20-14	S.PP	2.840	0.967	1
15-146	20-14	Sham	1.301	0.000	0
15-148	20-14	Sham	1.301	0.000	0
15-157	20-14	S.PP	2.456	0.967	1
15-159	20-14	S.PP	2.910	0.967	1
15-161	20-14	S.PP	2.146	0.967	1
15-169	20-14	S.PP	1.301	0.033	0
15-171	20-14	S.PP	2.053	0.967	1
15-173	20-14	S.PP	1.699	0.070	0
15-175	20-14	Sham	1.301	0.000	0
15-179	20-14	Sham	1.301	0.000	0
15-183	20-14	Sham	1.301	0.000	0
T434F	20-14	Sham	NA	0.152	0
T435F	20-14	Sham	NA	0.047	0
T455M	20-14	Sham	NA	0.174	0
T458M	20-14	Sham	NA	0.126	0
T459M	20-14	Sham	NA	0.000	0

psVNA: pseudotyped virus neutralization assay; IC_{50}: 50% neutralization antibody titer; R0: virus basic reproductive ratio; NA: not available

Panel g and h

Animal ID	Study #	Vaccine construct	ELISA (log_{10} EU/ml)	Nose_reduction_R0	Protection_nose
6418	20-07	Sham	1.702	0.000	0
6768	20-07	S.dCT	NA	0.967	1
6770	20-07	tPA.WT.S	NA	0.405	0
6855	20-07	tPA.S	2.479	0.967	1
6856	20-07	tPA.S	2.163	0.258	0
6905	20-07	S.dTM.PP	3.279	0.967	1
6906	20-07	S.dTM.PP	2.515	0.255	0
7028	20-07	tPA.S.PP	3.452	0.336	0
7056	20-07	tPA.S.PP	3.142	0.064	0
7123	20-07	Sham	NA	0.000	0
7130	20-07	Sham	NA	0.000	0
7145	20-07	Sham	NA	0.000	0
7154	20-07	Sham	NA	0.000	0
7157	20-07	Sham	NA	0.000	0
7245	20-07	Sham	NA	0.000	0
7261	20-07	Sham	NA	0.000	0
BM39	20-07	tPA.S	1.702	0.069	0
BO49	20-07	tPA.S	2.357	0.967	1
BO93	20-07	tPA.S.PP	3.130	0.967	1
BO95	20-07	tPA.S.PP	3.132	0.023	0
BP11	20-07	S.dCT	3.185	0.060	0
BS64	20-07	Sham	1.702	0.000	0
BS80	20-07	Sham	3.752	0.042	0
BS82	20-07	S	3.290	0.127	0
BV16	20-07	S	3.500	0.967	1
BV35	20-07	S.dCT	2.637	0.967	1
BV36	20-07	S.dCT	3.293	0.967	1
BV58	20-07	tPA.WT.S	3.662	0.489	0
BV79	20-07	tPA.WT.S	3.914	0.967	1
BV95	20-07	S.dTM.PP	4.288	0.967	1
BV96	20-07	S.dTM.PP	4.209	0.064	0
T781	20-07	S	2.822	0.331	0
T796	20-07	S	3.174	0.967	1
T799	20-07	tPA.WT.S	2.724	0.063	0
171170	20-09	S.dTM.PP	3.060	0.020	0
171219	20-09	Sham	1.702	0.000	0
171264	20-09	Sham	1.702	0.000	0
180662	20-09	S.PP	3.615	0.967	1
180689	20-09	S.PP	3.149	0.967	1
181267	20-09	S.PP	3.455	0.967	1
181302	20-09	S.PP	3.710	0.967	1
181343	20-09	S.PP	3.198	0.967	1
181363	20-09	S.PP	3.097	0.023	0

Animal ID	Study #	Vaccine construct	ELISA (log_{10} EU/ml)	Nose_reduction_R0	Protection_nose
191358	20-09	Sham	1.702	0.000	0
191372	20-09	Sham	1.702	0.000	0
T414	20-09	Sham	NA	0.000	0
T423	20-09	Sham	NA	0.015	0
T436	20-09	Sham	NA	0.044	0
T440	20-09	Sham	NA	0.099	0
T457	20-09	Sham	NA	0.000	0
T463	20-09	Sham	NA	0.000	0
15-072	20-14	S.PP	3.407	0.967	1
15-084	20-14	S.PP	3.687	0.967	1
15-086	20-14	S.PP	3.567	0.967	1
15-104	20-14	S.PP	2.785	0.026	0
15-108	20-14	S.PP	2.759	0.050	0
15-114	20-14	S.PP	2.746	0.322	0
15-115	20-14	S.PP	3.042	0.967	1
15-117	20-14	S.PP	3.507	0.967	1
15-118	20-14	S.PP	2.073	0.118	0
15-131	20-14	S.PP	3.277	0.967	1
15-139	20-14	S.PP	3.368	0.967	1
15-140	20-14	S.PP	1.702	0.039	0
15-143	20-14	S.PP	3.068	0.967	1
15-145	20-14	S.PP	3.041	0.967	1
15-146	20-14	Sham	1.702	0.000	0
15-148	20-14	Sham	1.702	0.000	0
15-157	20-14	S.PP	3.308	0.967	1
15-159	20-14	S.PP	3.496	0.967	1
15-161	20-14	S.PP	2.956	0.967	1
15-169	20-14	S.PP	1.702	0.033	0
15-171	20-14	S.PP	2.617	0.967	1
15-173	20-14	S.PP	1.958	0.070	0
15-175	20-14	Sham	1.702	0.000	0
15-179	20-14	Sham	1.702	0.000	0
15-183	20-14	Sham	1.702	0.000	0
T434F	20-14	Sham	NA	0.152	0
T435F	20-14	Sham	NA	0.047	0
T455M	20-14	Sham	NA	0.174	0
T458M	20-14	Sham	NA	0.126	0
T459M	20-14	Sham	NA	0.000	0

ELISA: enzyme-linked immunosorbent assay; EU/mL: ELISA unit per milliliter; R0: virus basic reproductive ratio; NA: not available

Supplementary Table 6: Source data file for Figure 3

Panel a: graphical representation of the 6-month durability study; source data not applicable

Panel b: Peak Viral Load (bronchoalveolar lavage [BAL])

Animal Group	Animal_ID	Study	Vaccine construct	Lung (BAL) peak viral load (Log_{10} sgRNA copies/mL)
1	158274	6-month durability study	Sham	4.43
1	158411	6-month durability study	Sham	2.66
1	158422	6-month durability study	Sham	4.02
1	1606400	6-month durability study	Sham	4.13
1	1680940	6-month durability study	Sham	3.74
1	1682980	6-month durability study	Sham	4.00
1	1683040	6-month durability study	Sham	5.32
2	158270	6-month durability study	1-dose $1x10^{11}$ vp Ad26.COV2.S	1.70
2	158272	6-month durability study	1-dose $1x10^{11}$ vp Ad26.COV2.S	1.70
2	158300	6-month durability study	1-dose $1x10^{11}$ vp Ad26.COV2.S	1.70
2	1606460	6-month durability study	1-dose $1x10^{11}$ vp Ad26.COV2.S	1.70
2	1681960	6-month durability study	1-dose $1x10^{11}$ vp Ad26.COV2.S	1.70
2	1683000	6-month durability study	1-dose $1x10^{11}$ vp Ad26.COV2.S	1.70
2	1683360	6-month durability study	1-dose $1x10^{11}$ vp Ad26.COV2.S	1.70
3	1506260	6-month durability study	1-dose $5x10^{10}$ vp Ad26.COV2.S	1.70
3	158284	6-month durability study	1-dose $5x10^{10}$ vp Ad26.COV2.S	3.36
3	158298	6-month durability study	1-dose $5x10^{10}$ vp Ad26.COV2.S	1.70
3	1680780	6-month durability study	1-dose $5x10^{10}$ vp Ad26.COV2.S	1.70
3	1682300	6-month durability study	1-dose $5x10^{10}$ vp Ad26.COV2.S	1.70
3	1683160	6-month durability study	1-dose $5x10^{10}$ vp Ad26.COV2.S	1.70
3	1801551	6-month durability study	1-dose $5x10^{10}$ vp Ad26.COV2.S	1.70
4	158278	6-month durability study	2-dose $5x10^{10}$ vp Ad26.COV2.S (0-4)	1.70
4	158282	6-month durability study	2-dose $5x10^{10}$ vp Ad26.COV2.S (0-4)	1.70
4	1600560	6-month durability study	2-dose $5x10^{10}$ vp Ad26.COV2.S (0-4)	1.70
4	1680120	6-month durability study	2-dose $5x10^{10}$ vp Ad26.COV2.S (0-4)	1.70
4	1683440	6-month durability study	2-dose $5x10^{10}$ vp Ad26.COV2.S (0-4)	1.70
4	1701220	6-month durability study	2-dose $5x10^{10}$ vp Ad26.COV2.S (0-4)	1.70
4	1706171	6-month durability study	2-dose $5x10^{10}$ vp Ad26.COV2.S (0-4)	1.70
5	158280	6-month durability study	2-dose $5x10^{10}$ vp Ad26.COV2.S (0-8)	1.70
5	1606520	6-month durability study	2-dose $5x10^{10}$ vp Ad26.COV2.S (0-8)	1.70
5	1680260	6-month durability study	2-dose $5x10^{10}$ vp Ad26.COV2.S (0-8)	1.70

Animal Group	Animal_ID	Study	Vaccine construct	Lung (BAL) peak viral load (Log$_{10}$ sgRNA copies/mL)
5	1682671	6-month durability study	2-dose 5x10^{10} vp Ad26.COV2.S (0-8)	3.87
5	1683140	6-month durability study	2-dose 5x10^{10} vp Ad26.COV2.S (0-8)	1.70
5	1683520	6-month durability study	2-dose 5x10^{10} vp Ad26.COV2.S (0-8)	3.32
5	1683640	6-month durability study	2-dose 5x10^{10} vp Ad26.COV2.S (0-8)	1.70

sgRNA: subgenomic RNA

Panel c: Peak Viral Load (nose)

Animal Group	Animal_ID	Study	Vaccine construct	Nose peak viral load (Log_{10} sgRNA copies/swab)
1	158274	6-month durability study	Sham	6.11
1	158411	6-month durability study	Sham	5.83
1	158422	6-month durability study	Sham	4.47
1	1606400	6-month durability study	Sham	5.04
1	1680940	6-month durability study	Sham	5.83
1	1682980	6-month durability study	Sham	5.64
1	1683040	6-month durability study	Sham	6.72
2	158270	6-month durability study	1-dose $1x10^{11}$ vp Ad26.COV2.S	4.32
2	158272	6-month durability study	1-dose $1x10^{11}$ vp Ad26.COV2.S	2.10
2	158300	6-month durability study	1-dose $1x10^{11}$ vp Ad26.COV2.S	5.72
2	1606460	6-month durability study	1-dose $1x10^{11}$ vp Ad26.COV2.S	5.50
2	1681960	6-month durability study	1-dose $1x10^{11}$ vp Ad26.COV2.S	5.19
2	1683000	6-month durability study	1-dose $1x10^{11}$ vp Ad26.COV2.S	5.58
2	1683360	6-month durability study	1-dose $1x10^{11}$ vp Ad26.COV2.S	4.96
3	1506260	6-month durability study	1-dose $5x10^{10}$ vp Ad26.COV2.S	5.78
3	158284	6-month durability study	1-dose $5x10^{10}$ vp Ad26.COV2.S	6.27
3	158298	6-month durability study	1-dose $5x10^{10}$ vp Ad26.COV2.S	2.10
3	1680780	6-month durability study	1-dose $5x10^{10}$ vp Ad26.COV2.S	6.18
3	1682300	6-month durability study	1-dose $5x10^{10}$ vp Ad26.COV2.S	6.88
3	1683160	6-month durability study	1-dose $5x10^{10}$ vp Ad26.COV2.S	4.98
3	1801551	6-month durability study	1-dose $5x10^{10}$ vp Ad26.COV2.S	5.50
4	158278	6-month durability study	2-dose $5x10^{10}$ vp Ad26.COV2.S (0-4)	5.44
4	158282	6-month durability study	2-dose $5x10^{10}$ vp Ad26.COV2.S (0-4)	5.15
4	1600560	6-month durability study	2-dose $5x10^{10}$ vp Ad26.COV2.S (0-4)	6.66
4	1680120	6-month durability study	2-dose $5x10^{10}$ vp Ad26.COV2.S (0-4)	5.52
4	1683440	6-month durability study	2-dose $5x10^{10}$ vp Ad26.COV2.S (0-4)	4.74
4	1701220	6-month durability study	2-dose $5x10^{10}$ vp Ad26.COV2.S (0-4)	6.42
4	1706171	6-month durability study	2-dose $5x10^{10}$ vp Ad26.COV2.S (0-4)	2.10
5	158280	6-month durability study	2-dose $5x10^{10}$ vp Ad26.COV2.S (0-8)	5.74
5	1606520	6-month durability study	2-dose $5x10^{10}$ vp Ad26.COV2.S (0-8)	3.22
5	1680260	6-month durability study	2-dose $5x10^{10}$ vp Ad26.COV2.S (0-8)	3.78
5	1682671	6-month durability study	2-dose $5x10^{10}$ vp Ad26.COV2.S (0-8)	2.10
5	1683140	6-month durability study	2-dose $5x10^{10}$ vp Ad26.COV2.S (0-8)	4.16

Animal Group	Animal_ID	Study	Vaccine construct	Nose peak viral load (Log_{10} sgRNA copies/swab)
5	1683520	6-month durability study	2-dose $5x10^{10}$ vp Ad26.COV2.S (0-8)	5.75
5	1683640	6-month durability study	2-dose $5x10^{10}$ vp Ad26.COV2.S (0-8)	5.20

sgRNA: subgenomic RNA

Panel d: pseudotyped virus neutralization assay

Animal Group	Animal_ID	Study	Vaccine	psVNA (N50 titer)
1	158274	6-month durability study	Sham	20
1	158411	6-month durability study	Sham	20
1	158422	6-month durability study	Sham	20
1	1606400	6-month durability study	Sham	20
1	1680940	6-month durability study	Sham	20
1	1682980	6-month durability study	Sham	20
1	1683040	6-month durability study	Sham	20
2	158270	6-month durability study	1-dose $1x10^{11}$ vp Ad26.COV2.S	254
2	158272	6-month durability study	1-dose $1x10^{11}$ vp Ad26.COV2.S	491
2	158300	6-month durability study	1-dose $1x10^{11}$ vp Ad26.COV2.S	255
2	1606460	6-month durability study	1-dose $1x10^{11}$ vp Ad26.COV2.S	662
2	1681960	6-month durability study	1-dose $1x10^{11}$ vp Ad26.COV2.S	733
2	1683000	6-month durability study	1-dose $1x10^{11}$ vp Ad26.COV2.S	811
2	1683360	6-month durability study	1-dose $1x10^{11}$ vp Ad26.COV2.S	632
3	1506260	6-month durability study	1-dose $5x10^{10}$ vp Ad26.COV2.S	83
3	158284	6-month durability study	1-dose $5x10^{10}$ vp Ad26.COV2.S	140
3	158298	6-month durability study	1-dose $5x10^{10}$ vp Ad26.COV2.S	201
3	1680780	6-month durability study	1-dose $5x10^{10}$ vp Ad26.COV2.S	58
3	1682300	6-month durability study	1-dose $5x10^{10}$ vp Ad26.COV2.S	141
3	1683160	6-month durability study	1-dose $5x10^{10}$ vp Ad26.COV2.S	194
3	1801551	6-month durability study	1-dose $5x10^{10}$ vp Ad26.COV2.S	143
4	158278	6-month durability study	2-dose $5x10^{10}$ vp Ad26.COV2.S (0-4)	316
4	158282	6-month durability study	2-dose $5x10^{10}$ vp Ad26.COV2.S (0-4)	134
4	1600560	6-month durability study	2-dose $5x10^{10}$ vp Ad26.COV2.S (0-4)	173
4	1680120	6-month durability study	2-dose $5x10^{10}$ vp Ad26.COV2.S (0-4)	123
4	1683440	6-month durability study	2-dose $5x10^{10}$ vp Ad26.COV2.S (0-4)	277
4	1701220	6-month durability study	2-dose $5x10^{10}$ vp Ad26.COV2.S (0-4)	289
4	1706171	6-month durability study	2-dose $5x10^{10}$ vp Ad26.COV2.S (0-4)	129
5	158280	6-month durability study	2-dose $5x10^{10}$ vp Ad26.COV2.S (0-8)	224
5	1606520	6-month durability study	2-dose $5x10^{10}$ vp Ad26.COV2.S (0-8)	2306
5	1680260	6-month durability study	2-dose $5x10^{10}$ vp Ad26.COV2.S (0-8)	348
5	1682671	6-month durability study	2-dose $5x10^{10}$ vp Ad26.COV2.S (0-8)	145
5	1683140	6-month durability study	2-dose $5x10^{10}$ vp Ad26.COV2.S (0-8)	677
5	1683520	6-month durability study	2-dose $5x10^{10}$ vp Ad26.COV2.S (0-8)	192

**Animal Group**	**Animal_ID**	**Study**	**Vaccine**	**psVNA (N50 titer)**
5	1683640	6-month durability study	2-dose $5x10^{10}$ vp Ad26.COV2.S (0-8)	311

psVNA: pseudotyped virus neutralization assay

Panel e: Spike protein (S) Enzyme-linked immunosorbent assay (ELISA)

Animal Group	Animal_ID	Study	Vaccine	S ELISA (EU/mL)
1	1680940	6-month durability study	Sham	3.4
1	1682980	6-month durability study	Sham	3.6
1	1683040	6-month durability study	Sham	5.1
1	158274	6-month durability study	Sham	3.4
1	158411	6-month durability study	Sham	3.4
1	1606400	6-month durability study	Sham	3.4
1	158422	6-month durability study	Sham	3.4
2	1683000	6-month durability study	1-dose $1x10^{11}$ vp Ad26.COV2.S	1187.9
2	158300	6-month durability study	1-dose $1x10^{11}$ vp Ad26.COV2.S	894
2	1681960	6-month durability study	1-dose $1x10^{11}$ vp Ad26.COV2.S	1703.6
2	1683360	6-month durability study	1-dose $1x10^{11}$ vp Ad26.COV2.S	1223.5
2	158272	6-month durability study	1-dose $1x10^{11}$ vp Ad26.COV2.S	623
2	1606460	6-month durability study	1-dose $1x10^{11}$ vp Ad26.COV2.S	419.3
2	158270	6-month durability study	1-dose $1x10^{11}$ vp Ad26.COV2.S	999.6
3	1801551	6-month durability study	1-dose $5x10^{10}$ vp Ad26.COV2.S	775.4
3	158284	6-month durability study	1-dose $5x10^{10}$ vp Ad26.COV2.S	415
3	1683160	6-month durability study	1-dose $5x10^{10}$ vp Ad26.COV2.S	810.7
3	1682300	6-month durability study	1-dose $5x10^{10}$ vp Ad26.COV2.S	366
3	1506260	6-month durability study	1-dose $5x10^{10}$ vp Ad26.COV2.S	86.3
3	1680780	6-month durability study	1-dose $5x10^{10}$ vp Ad26.COV2.S	149.9
3	158298	6-month durability study	1-dose $5x10^{10}$ vp Ad26.COV2.S	237.7
4	1706171	6-month durability study	2-dose $5x10^{10}$ vp Ad26.COV2.S (0-4)	1524.2
4	158282	6-month durability study	2-dose $5x10^{10}$ vp Ad26.COV2.S (0-4)	365.8
4	1600560	6-month durability study	2-dose $5x10^{10}$ vp Ad26.COV2.S (0-4)	413.9
4	1701220	6-month durability study	2-dose $5x10^{10}$ vp Ad26.COV2.S (0-4)	914.8
4	1680120	6-month durability study	2-dose $5x10^{10}$ vp Ad26.COV2.S (0-4)	336.5
4	158278	6-month durability study	2-dose $5x10^{10}$ vp Ad26.COV2.S (0-4)	709
4	1683440	6-month durability study	2-dose $5x10^{10}$ vp Ad26.COV2.S (0-4)	875.1
5	1682671	6-month durability study	2-dose $5x10^{10}$ vp Ad26.COV2.S (0-8)	829.6
5	1683640	6-month durability study	2-dose $5x10^{10}$ vp Ad26.COV2.S (0-8)	259.3
5	1606520	6-month durability study	2-dose $5x10^{10}$ vp Ad26.COV2.S (0-8)	1608.7
5	1683520	6-month durability study	2-dose $5x10^{10}$ vp Ad26.COV2.S (0-8)	376.2
5	1683140	6-month durability study	2-dose $5x10^{10}$ vp Ad26.COV2.S (0-8)	1800.2
5	1680260	6-month durability study	2-dose $5x10^{10}$ vp Ad26.COV2.S (0-8)	780.9

Animal Group	Animal_ID	Study	Vaccine	S ELISA (EU/mL)
5	158280	6-month durability study	2-dose $5x10^{10}$ vp Ad26.COV2.S (0-8)	708.5

S-ELISA: Spike protein Enzyme-linked immunosorbent assay; EU/mL: ELISA units per milliliter

Panel f

		Lung One-dose	Lung Two-dose	Lung Combined	Nose One-dose	Nose Two-dose	Nose Combined
**S-ELISA**	Observed (Mean)	92.9%	85.7%	89.3%	14.3%	14.3%	14.30%
	[95% CI]	[58.0-99.2%]	[53.5-96.9%]	[70.4-96.7%]	[3.1-46.5%]	[3.1-46.5%]	[5.2-33.5%]
	Prediction Analysis A (Mean)	88.0%	90.5%	89.3%	47.4%	53.7%	50.60%
	[95% CI]	[69.4-96.4%]	[76.7-96.8%]	[74.9-96.4%]	[20.1-76.5%]	[24.6-80.9%]	[25.0-76.6%]
	Prediction Analysis B (Mean)	99.0%	100%	100%	15.0%	19.0%	17.00%
	[95% CI]	[93.0-100%]	[100-100%]	[100-100%]	[0.0-56.0%]	[0.0-64.0%]	[0.0-61.0%]
**psVNA**	Observed (Mean)	92.9%	85.7%	89.3%	14.3%	14.3%	14.30%
	[95% CI]	[58-99.2%]	[53.5-96.9%]	[70.4-96.7%]	[3.1-46.5%]	[3.1-46.5%]	[5.2-33.5%]
	Prediction Analysis A (Mean)	93.5%	94.3%	93.9%	75.1%	79.1%	77.10%
	[95% CI]	[86.9-98.3%]	[89.5-98.3%]	[86.3-98.3%]	[52.9-95.7%]	[58.4-97.5%]	[59.7-93.9%]
	Prediction Analysis B (Mean)	87%	95.0%	91.0%	77.0%	82.0%	80.00%
	[95% CI]	[64.0-100%]	[64.0-100%]	[64.0-100%]	[36.0-86.0%]	[14.0-100%]	[25.0-93.0%]

S-ELISA: Spike protein Enzyme-linked immunosorbent assay; psVNA: pseudotyped virus neutralization assay; CI: confidence interval

Supplementary Table 7: Source data file for Supplementary Figure 1

Panel a: graphical representation of studies 20-07, 20-09 and 20-124; source data not applicable

Panel b: Study 20-07 S ELISA Nexelis

Animal Group	Animal_ID	Study	Vaccine	Timepoint	S ELISA (EU/mL)
1	6855	20-07	Ad26NCOV002	0	50.3
1	6856	20-07	Ad26NCOV002	0	50.3
1	BM39	20-07	Ad26NCOV002	0	50.3
1	BO49	20-07	Ad26NCOV002	0	50.3
1	6855	20-07	Ad26NCOV002	2	551.3
1	6856	20-07	Ad26NCOV002	2	50.3
1	BM39	20-07	Ad26NCOV002	2	50.3
1	BO49	20-07	Ad26NCOV002	2	50.3
1	6855	20-07	Ad26NCOV002	4	301.5
1	6856	20-07	Ad26NCOV002	4	145.5
1	BM39	20-07	Ad26NCOV002	4	50.3
1	BO49	20-07	Ad26NCOV002	4	227.7
2	7028	20-07	Ad26NCOV004	0	50.3
2	7056	20-07	Ad26NCOV004	0	50.3
2	BO93	20-07	Ad26NCOV004	0	50.3
2	BO95	20-07	Ad26NCOV004	0	256.5
2	7028	20-07	Ad26NCOV004	2	1461
2	7056	20-07	Ad26NCOV004	2	291.7
2	BO93	20-07	Ad26NCOV004	2	299.6
2	BO95	20-07	Ad26NCOV004	2	403
2	7028	20-07	Ad26NCOV004	4	2828.9
2	7056	20-07	Ad26NCOV004	4	1387.5
2	BO93	20-07	Ad26NCOV004	4	1350.5
2	BO95	20-07	Ad26NCOV004	4	1354.1
3	BS82	20-07	Ad26NCOV006	0	1840.2
3	BV16	20-07	Ad26NCOV006	0	4018.6
3	T781	20-07	Ad26NCOV006	0	50.3
3	T796	20-07	Ad26NCOV006	0	50.3
3	BS82	20-07	Ad26NCOV006	2	1751.3
3	BV16	20-07	Ad26NCOV006	2	3497.2
3	T781	20-07	Ad26NCOV006	2	329
3	T796	20-07	Ad26NCOV006	2	166.5
3	BS82	20-07	Ad26NCOV006	4	1951.4
3	BV16	20-07	Ad26NCOV006	4	3164.4
3	T781	20-07	Ad26NCOV006	4	663.1
3	T796	20-07	Ad26NCOV006	4	1492.1
4	BP11	20-07	Ad26NCOV008	0	971.7
4	BV35	20-07	Ad26NCOV008	0	76.6
4	BV36	20-07	Ad26NCOV008	0	1770.1

Animal Group	Animal_ID	Study	Vaccine	Timepoint	S ELISA (EU/mL)
4	BP11	20-07	Ad26NCOV008	2	1028.3
4	BV35	20-07	Ad26NCOV008	2	119.5
4	BV36	20-07	Ad26NCOV008	2	1897.8
4	BP11	20-07	Ad26NCOV008	4	1532.6
4	BV35	20-07	Ad26NCOV008	4	433.5
4	BV36	20-07	Ad26NCOV008	4	1961.6
5	BV58	20-07	Ad26NCOV014	0	3050.3
5	BV79	20-07	Ad26NCOV014	0	7493.6
5	T799	20-07	Ad26NCOV014	0	50.3
5	BV58	20-07	Ad26NCOV014	2	3398.3
5	BV79	20-07	Ad26NCOV014	2	6754.2
5	T799	20-07	Ad26NCOV014	2	123.6
5	BV58	20-07	Ad26NCOV014	4	4590.7
5	BV79	20-07	Ad26NCOV014	4	8206.4
5	T799	20-07	Ad26NCOV014	4	529.5
7	6905	20-07	Ad26NCOV028	0	50.3
7	6906	20-07	Ad26NCOV028	0	50.3
7	BV95	20-07	Ad26NCOV028	0	8058
7	BV96	20-07	Ad26NCOV028	0	10480.8
7	6905	20-07	Ad26NCOV028	2	800
7	6906	20-07	Ad26NCOV028	2	103.7
7	BV95	20-07	Ad26NCOV028	2	14179.8
7	BV96	20-07	Ad26NCOV028	2	15499
7	6905	20-07	Ad26NCOV028	4	1900.9
7	6906	20-07	Ad26NCOV028	4	327.7
7	BV95	20-07	Ad26NCOV028	4	19423.7
7	BV96	20-07	Ad26NCOV028	4	16187.3
8	6418	20-07	sham	0	50.3
8	BS64	20-07	sham	0	50.3
8	BS80	20-07	sham	0	8099.5
8	6418	20-07	sham	2	50.3
8	BS64	20-07	sham	2	50.3
8	BS80	20-07	sham	2	5887.5
8	6418	20-07	sham	4	50.3
8	BS64	20-07	sham	4	50.3
8	BS80	20-07	sham	4	5651.8

S-ELISA: Spike protein Enzyme-linked immunosorbent assay; EU/mL: ELISA units per milliliter

Panel c: Study 20-09 (S ELISA Nexelis)

Animal Group	Animal_ID	Study	Vaccine	Timepoint	S ELISA (EU/mL)
1	171170	20-09	Ad26NCOV028	0	50.3
1	180215	20-09	Ad26NCOV028	0	50.3
1	171170	20-09	Ad26NCOV028	2	173.7
1	180215	20-09	Ad26NCOV028	2	101.2
1	171170	20-09	Ad26NCOV028	4	1149.1
1	180215	20-09	Ad26NCOV028	4	504.3
2	180662	20-09	Ad26NCOV030	0	50.3
2	180689	20-09	Ad26NCOV030	0	50.3
2	181267	20-09	Ad26NCOV030	0	50.3
2	181302	20-09	Ad26NCOV030	0	50.3
2	181343	20-09	Ad26NCOV030	0	50.3
2	181363	20-09	Ad26NCOV030	0	50.3
2	180662	20-09	Ad26NCOV030	2	1625.1
2	180689	20-09	Ad26NCOV030	2	450.1
2	181267	20-09	Ad26NCOV030	2	399.9
2	181302	20-09	Ad26NCOV030	2	1135.1
2	181343	20-09	Ad26NCOV030	2	1019.3
2	181363	20-09	Ad26NCOV030	2	334.4
2	180662	20-09	Ad26NCOV030	4	4122.7
2	180689	20-09	Ad26NCOV030	4	1410.6
2	181267	20-09	Ad26NCOV030	4	2851.5
2	181302	20-09	Ad26NCOV030	4	5124.3
2	181343	20-09	Ad26NCOV030	4	1576.1
2	181363	20-09	Ad26NCOV030	4	1249.4
3	171219	20-09	Sham	0	50.3
3	171264	20-09	Sham	0	50.3
3	191358	20-09	Sham	0	50.3
3	191372	20-09	Sham	0	50.3
3	171219	20-09	Sham	2	50.3
3	171264	20-09	Sham	2	50.3
3	191358	20-09	Sham	2	50.3
3	191372	20-09	Sham	2	50.3
3	171219	20-09	Sham	4	50.3
3	171264	20-09	Sham	4	50.3
3	191358	20-09	Sham	4	50.3
3	191372	20-09	Sham	4	50.3

S-ELISA: Spike protein Enzyme-linked immunosorbent assay; EU/mL: ELISA units per milliliter

Panel d: Study 20-14 (S ELISA Nexelis)

Animal Group	Animal_ID	Study	Vaccine	Timepoint	S ELISA (EU/mL)
1	15-072	20-14	$1x10^{11}$ vp Ad26NCOV030	0	50.3
1	15-084	20-14	$1x10^{11}$ vp Ad26NCOV030	0	50.3
1	15-115	20-14	$1x10^{11}$ vp Ad26NCOV030	0	50.3
1	15-117	20-14	$1x10^{11}$ vp Ad26NCOV030	0	50.3
1	15-131	20-14	$1x10^{11}$ vp Ad26NCOV030	0	50.3
1	15-072	20-14	$1x10^{11}$ vp Ad26NCOV030	2	1289.6
1	15-084	20-14	$1x10^{11}$ vp Ad26NCOV030	2	1483.2
1	15-115	20-14	$1x10^{11}$ vp Ad26NCOV030	2	176.1
1	15-117	20-14	$1x10^{11}$ vp Ad26NCOV030	2	580.2
1	15-131	20-14	$1x10^{11}$ vp Ad26NCOV030	2	671.4
1	15-072	20-14	$1x10^{11}$ vp Ad26NCOV030	4	2553.8
1	15-084	20-14	$1x10^{11}$ vp Ad26NCOV030	4	4859.6
1	15-115	20-14	$1x10^{11}$ vp Ad26NCOV030	4	1102.8
1	15-117	20-14	$1x10^{11}$ vp Ad26NCOV030	4	3216.8
1	15-131	20-14	$1x10^{11}$ vp Ad26NCOV030	4	1894.2
2	15-086	20-14	$5x10^{10}$ vp Ad26NCOV030	0	50.3
2	15-104	20-14	$5x10^{10}$ vp Ad26NCOV030	0	50.3
2	15-139	20-14	$5x10^{10}$ vp Ad26NCOV030	0	50.3
2	15-143	20-14	$5x10^{10}$ vp Ad26NCOV030	0	50.3
2	15-145	20-14	$5x10^{10}$ vp Ad26NCOV030	0	50.3
2	15-086	20-14	$5x10^{10}$ vp Ad26NCOV030	2	3061.8
2	15-104	20-14	$5x10^{10}$ vp Ad26NCOV030	2	218.7
2	15-139	20-14	$5x10^{10}$ vp Ad26NCOV030	2	330.2
2	15-143	20-14	$5x10^{10}$ vp Ad26NCOV030	2	636
2	15-145	20-14	$5x10^{10}$ vp Ad26NCOV030	2	425.4
2	15-086	20-14	$5x10^{10}$ vp Ad26NCOV030	4	3687.3
2	15-104	20-14	$5x10^{10}$ vp Ad26NCOV030	4	610
2	15-139	20-14	$5x10^{10}$ vp Ad26NCOV030	4	2332.7
2	15-143	20-14	$5x10^{10}$ vp Ad26NCOV030	4	1170
2	15-145	20-14	$5x10^{10}$ vp Ad26NCOV030	4	1099.8
3	15-108	20-14	$1.125x10^{10}$ vp Ad26NCOV030	0	50.3
3	15-114	20-14	$1.125x10^{10}$ vp Ad26NCOV030	0	50.3
3	15-157	20-14	$1.125x10^{10}$ vp Ad26NCOV030	0	50.3
3	15-159	20-14	$1.125x10^{10}$ vp Ad26NCOV030	0	50.3
3	15-161	20-14	$1.125x10^{10}$ vp Ad26NCOV030	0	50.3
3	15-108	20-14	$1.125x10^{10}$ vp Ad26NCOV030	2	318.9
3	15-114	20-14	$1.125x10^{10}$ vp Ad26NCOV030	2	310.4
3	15-157	20-14	$1.125x10^{10}$ vp Ad26NCOV030	2	1962.1

Animal Group	Animal_ID	Study	Vaccine	Timepoint	S ELISA (EU/mL)
3	15-159	20-14	$1.125x10^{10}$ vp Ad26NCOV030	2	665.7
3	15-161	20-14	$1.125x10^{10}$ vp Ad26NCOV030	2	134.8
3	15-108	20-14	$1.125x10^{10}$ vp Ad26NCOV030	4	573.5
3	15-114	20-14	$1.125x10^{10}$ vp Ad26NCOV030	4	557.5
3	15-157	20-14	$1.125x10^{10}$ vp Ad26NCOV030	4	2031.4
3	15-159	20-14	$1.125x10^{10}$ vp Ad26NCOV030	4	3134.3
3	15-161	20-14	$1.125x10^{10}$ vp Ad26NCOV030	4	904.5
4	15-118	20-14	$2x10^{9}$ vp Ad26NCOV030	0	50.3
4	15-140	20-14	$2x10^{9}$ vp Ad26NCOV030	0	50.3
4	15-169	20-14	$2x10^{9}$ vp Ad26NCOV030	0	50.3
4	15-171	20-14	$2x10^{9}$ vp Ad26NCOV030	0	50.3
4	15-173	20-14	$2x10^{9}$ vp Ad26NCOV030	0	50.3
4	15-118	20-14	$2x10^{9}$ vp Ad26NCOV030	2	50.3
4	15-140	20-14	$2x10^{9}$ vp Ad26NCOV030	2	50.3
4	15-169	20-14	$2x10^{9}$ vp Ad26NCOV030	2	50.3
4	15-171	20-14	$2x10^{9}$ vp Ad26NCOV030	2	50.3
4	15-173	20-14	$2x10^{9}$ vp Ad26NCOV030	2	50.3
4	15-118	20-14	$2x10^{9}$ vp Ad26NCOV030	4	118.3
4	15-140	20-14	$2x10^{9}$ vp Ad26NCOV030	4	50.3
4	15-169	20-14	$2x10^{9}$ vp Ad26NCOV030	4	50.3
4	15-171	20-14	$2x10^{9}$ vp Ad26NCOV030	4	413.7
4	15-173	20-14	$2x10^{9}$ vp Ad26NCOV030	4	90.8
5	15-146	20-14	Sham	0	50.3
5	15-148	20-14	Sham	0	50.3
5	15-175	20-14	Sham	0	50.3
5	15-179	20-14	Sham	0	50.3
5	15-183	20-14	Sham	0	50.3
5	15-146	20-14	Sham	2	50.3
5	15-148	20-14	Sham	2	50.3
5	15-175	20-14	Sham	2	50.3
5	15-179	20-14	Sham	2	50.3
5	15-183	20-14	Sham	2	50.3
5	15-146	20-14	Sham	4	50.3
5	15-148	20-14	Sham	4	50.3
5	15-175	20-14	Sham	4	50.3
5	15-179	20-14	Sham	4	50.3
5	15-183	20-14	Sham	4	50.3

S-ELISA: Spike protein Enzyme-linked immunosorbent assay; EU/mL: ELISA units per milliliter

Supplementary Table 8: Source data file for Supplementary Figure 2

Panel a and b

Animal ID	Study #	Vaccine construct	Lung peak viral load (copies/mL)	ELISpot (log_{10} spots/10^6 PBMC)	Protection_lung
6418	20-07	Sham	4.543	1.000	0
6768	20-07	S.dCT	≤LOD	2.631	1
6770	20-07	tPA.WT.S	≤LOD	2.618	1
6855	20-07	tPA.S	≤LOD	2.380	1
6856	20-07	tPA.S	5.097	2.146	0
6905	20-07	S.dTM.PP	≤LOD	2.756	1
6906	20-07	S.dTM.PP	3.812	1.813	0
7028	20-07	tPA.S.PP	3.341	2.183	0
7056	20-07	tPA.S.PP	4.641	3.020	0
7123	20-07	Sham	5.575	NA	0
7130	20-07	Sham	4.895	NA	0
7145	20-07	Sham	6.103	NA	0
7154	20-07	Sham	4.615	NA	0
7157	20-07	Sham	4.871	NA	0
7245	20-07	Sham	6.288	NA	0
7261	20-07	Sham	5.400	NA	0
BM39	20-07	tPA.S	4.170	2.703	0
BO49	20-07	tPA.S	≤LOD	2.183	1
BO93	20-07	tPA.S.PP	≤LOD	2.720	1
BO95	20-07	tPA.S.PP	≤LOD	2.553	1
BP11	20-07	S.dCT	3.514	2.588	0
BS64	20-07	Sham	4.036	1.000	0
BS80	20-07	Sham	4.927	1.477	0
BS82	20-07	S	3.636	1.398	0
BV16	20-07	S	≤LOD	2.929	1
BV35	20-07	S.dCT	≤LOD	2.789	1
BV36	20-07	S.dCT	≤LOD	2.423	1
BV58	20-07	tPA.WT.S	≤LOD	2.789	1
BV79	20-07	tPA.WT.S	≤LOD	2.626	1
BV95	20-07	S.dTM.PP	≤LOD	2.505	1
BV96	20-07	S.dTM.PP	3.070	2.818	0
T781	20-07	S	3.733	1.398	0
T796	20-07	S	≤LOD	2.656	1
T799	20-07	tPA.WT.S	≤LOD	1.512	1
171170	20-09	S.dTM.PP	≤LOD	2.217	1
171219	20-09	Sham	4.934	1.000	0
171264	20-09	Sham	3.958	1.000	0
180662	20-09	S.PP	≤LOD	2.491	1
180689	20-09	S.PP	≤LOD	2.041	1
181267	20-09	S.PP	≤LOD	2.130	1
181302	20-09	S.PP	≤LOD	1.845	1

Animal ID	Study #	Vaccine construct	Lung peak viral load (copies/mL)	ELISpot (log_{10} spots/10^6 PBMC)	Protection_lung
181343	20-09	S.PP	≤LOD	1.544	1
181363	20-09	S.PP	≤LOD	1.740	1
191358	20-09	Sham	5.912	1.176	0
191372	20-09	Sham	4.443	1.301	0
T414	20-09	Sham	3.846	NA	0
T423	20-09	Sham	3.587	NA	0
T436	20-09	Sham	6.510	NA	0
T440	20-09	Sham	5.316	NA	0
T457	20-09	Sham	4.614	NA	0
T463	20-09	Sham	4.592	NA	0
15-072	20-14	S.PP	≤LOD	2.525	1
15-084	20-14	S.PP	≤LOD	2.130	1
15-086	20-14	S.PP	≤LOD	2.217	1
15-104	20-14	S.PP	≤LOD	1.699	1
15-108	20-14	S.PP	≤LOD	1.544	1
15-114	20-14	S.PP	2.623	1.176	0
15-115	20-14	S.PP	≤LOD	1.301	1
15-117	20-14	S.PP	≤LOD	1.602	1
15-118	20-14	S.PP	≤LOD	1.602	1
15-131	20-14	S.PP	≤LOD	2.000	1
15-139	20-14	S.PP	≤LOD	2.342	1
15-140	20-14	S.PP	3.962	1.000	0
15-143	20-14	S.PP	≤LOD	1.000	1
15-145	20-14	S.PP	≤LOD	1.301	1
15-146	20-14	Sham	4.691	1.000	0
15-148	20-14	Sham	3.164	1.000	0
15-157	20-14	S.PP	≤LOD	1.544	1
15-159	20-14	S.PP	≤LOD	1.740	1
15-161	20-14	S.PP	≤LOD	1.000	1
15-169	20-14	S.PP	≤LOD	1.000	1
15-171	20-14	S.PP	≤LOD	1.000	1
15-173	20-14	S.PP	≤LOD	1.929	1
15-175	20-14	Sham	5.064	1.000	0
15-179	20-14	Sham	3.877	1.000	0
15-183	20-14	Sham	3.888	1.000	0
T434F	20-14	Sham	2.696	NA	0
T435F	20-14	Sham	4.277	NA	0
T455M	20-14	Sham	3.905	NA	0
T458M	20-14	Sham	6.487	NA	0
T459M	20-14	Sham	4.182	NA	0

ELISpot: enzyme-linked immunospot assay; PBMC: peripheral blood mononuclear cells; LOD: limit of detection; NA: not available

Panel c and d

Animal ID	Study #	Vaccine construct	Nose peak viral load (copies/swab)	ELISpot (log_{10} spots/10^6 PBMC)	Protection_nose
6418	20-07	Sham	6.209	1.000	0
6768	20-07	S.dCT	≤LOD	2.631	1
6770	20-07	tPA.WT.S	6.162	2.618	0
6855	20-07	tPA.S	≤LOD	2.380	1
6856	20-07	tPA.S	5.087	2.146	0
6905	20-07	S.dTM.PP	≤LOD	2.756	1
6906	20-07	S.dTM.PP	6.104	1.813	0
7028	20-07	tPA.S.PP	5.965	2.183	0
7056	20-07	tPA.S.PP	5.701	3.020	0
7123	20-07	Sham	6.345	NA	0
7130	20-07	Sham	5.386	NA	0
7145	20-07	Sham	5.632	NA	0
7154	20-07	Sham	NA	NA	NA
7157	20-07	Sham	5.958	NA	0
7245	20-07	Sham	4.726	NA	0
7261	20-07	Sham	6.089	NA	0
BM39	20-07	tPA.S	6.513	2.703	0
BO49	20-07	tPA.S	≤LOD	2.183	1
BO93	20-07	tPA.S.PP	≤LOD	2.720	1
BO95	20-07	tPA.S.PP	5.031	2.553	0
BP11	20-07	S.dCT	5.930	2.588	0
BS64	20-07	Sham	4.485	1.000	0
BS80	20-07	Sham	5.111	1.477	0
BS82	20-07	S	5.078	1.398	0
BV16	20-07	S	≤LOD	2.929	1
BV35	20-07	S.dCT	≤LOD	2.789	1
BV36	20-07	S.dCT	≤LOD	2.423	1
BV58	20-07	tPA.WT.S	5.620	2.789	0
BV79	20-07	tPA.WT.S	≤LOD	2.626	1
BV95	20-07	S.dTM.PP	≤LOD	2.505	1
BV96	20-07	S.dTM.PP	5.785	2.818	0
T781	20-07	S	6.220	1.398	0
T796	20-07	S	≤LOD	2.656	1
T799	20-07	tPA.WT.S	5.951	1.512	0
171170	20-09	S.dTM.PP	3.858	2.217	0
171219	20-09	Sham	6.342	1.000	0
171264	20-09	Sham	6.705	1.000	0
180662	20-09	S.PP	≤LOD	2.491	1
180689	20-09	S.PP	≤LOD	2.041	1
181267	20-09	S.PP	≤LOD	2.130	1
181302	20-09	S.PP	≤LOD	1.845	1
181343	20-09	S.PP	≤LOD	1.544	1

Animal ID	Study #	Vaccine construct	Nose peak viral load (copies/swab)	ELISpot (log_{10} spots/10^6 PBMC)	Protection_nose
181363	20-09	S.PP	3.604	1.740	0
191358	20-09	Sham	8.010	1.176	0
191372	20-09	Sham	6.838	1.301	0
T414	20-09	Sham	4.989	NA	0
T423	20-09	Sham	5.199	NA	0
T436	20-09	Sham	5.587	NA	0
T440	20-09	Sham	4.073	NA	0
T457	20-09	Sham	3.781	NA	0
T463	20-09	Sham	5.709	NA	0
15-072	20-14	S.PP	≤LOD	2.525	1
15-084	20-14	S.PP	≤LOD	2.130	1
15-086	20-14	S.PP	≤LOD	2.217	1
15-104	20-14	S.PP	4.265	1.699	0
15-108	20-14	S.PP	4.981	1.544	0
15-114	20-14	S.PP	3.639	1.176	0
15-115	20-14	S.PP	≤LOD	1.301	1
15-117	20-14	S.PP	≤LOD	1.602	1
15-118	20-14	S.PP	5.460	1.602	0
15-131	20-14	S.PP	≤LOD	2.000	1
15-139	20-14	S.PP	≤LOD	2.342	1
15-140	20-14	S.PP	5.569	1.000	0
15-143	20-14	S.PP	≤LOD	1.000	1
15-145	20-14	S.PP	≤LOD	1.301	1
15-146	20-14	Sham	6.154	1.000	0
15-148	20-14	Sham	6.895	1.000	0
15-157	20-14	S.PP	≤LOD	1.544	1
15-159	20-14	S.PP	≤LOD	1.740	1
15-161	20-14	S.PP	≤LOD	1.000	1
15-169	20-14	S.PP	6.187	1.000	0
15-171	20-14	S.PP	≤LOD	1.000	1
15-173	20-14	S.PP	4.763	1.929	0
15-175	20-14	Sham	5.786	1.000	0
15-179	20-14	Sham	3.215	1.000	0
15-183	20-14	Sham	6.093	1.000	0
T434F	20-14	Sham	5.096	NA	0
T435F	20-14	Sham	5.926	NA	0
T455M	20-14	Sham	5.239	NA	0
T458M	20-14	Sham	5.324	NA	0
T459M	20-14	Sham	6.465	NA	0

ELISpot: enzyme-linked immunospot assay; PBMC: peripheral blood mononuclear cells; LOD: limit of detection; NA: not available

Panel e and f

Animal ID	Study #	Vaccine construct	ELISpot (log_{10} spots/10^6 PBMC)	Lung_reduction_R0	Protection_lung
6418	20-07	Sham	1.000	0.001	0
6768	20-07	S.dCT	2.631	0.973	1
6770	20-07	tPA.WT.S	2.618	0.973	1
6855	20-07	tPA.S	2.380	0.973	1
6856	20-07	tPA.S	2.146	0.170	0
6905	20-07	S.dTM.PP	2.756	0.973	1
6906	20-07	S.dTM.PP	1.813	0.172	0
7028	20-07	tPA.S.PP	2.183	0.002	0
7056	20-07	tPA.S.PP	3.020	0.001	0
7123	20-07	Sham	NA	0.262	0
7130	20-07	Sham	NA	0.297	0
7145	20-07	Sham	NA	0.168	0
7154	20-07	Sham	NA	0.122	0
7157	20-07	Sham	NA	0.001	0
7245	20-07	Sham	NA	0.001	0
7261	20-07	Sham	NA	0.001	0
BM39	20-07	tPA.S	2.703	0.002	0
BO49	20-07	tPA.S	2.183	0.973	1
BO93	20-07	tPA.S.PP	2.720	0.973	1
BO95	20-07	tPA.S.PP	2.553	0.973	1
BP11	20-07	S.dCT	2.588	0.214	0
BS64	20-07	Sham	1.000	0.002	0
BS80	20-07	Sham	1.477	0.001	0
BS82	20-07	S	1.398	0.645	0
BV16	20-07	S	2.929	0.973	1
BV35	20-07	S.dCT	2.789	0.973	1
BV36	20-07	S.dCT	2.423	0.973	1
BV58	20-07	tPA.WT.S	2.789	0.973	1
BV79	20-07	tPA.WT.S	2.626	0.973	1
BV95	20-07	S.dTM.PP	2.505	0.973	1
BV96	20-07	S.dTM.PP	2.818	0.002	0
T781	20-07	S	1.398	0.183	0
T796	20-07	S	2.656	0.973	1
T799	20-07	tPA.WT.S	1.512	0.973	1
171170	20-09	S.dTM.PP	2.217	0.973	1
171219	20-09	Sham	1.000	0.001	0
171264	20-09	Sham	1.000	0.001	0
180662	20-09	S.PP	2.491	0.973	1
180689	20-09	S.PP	2.041	0.973	1
181267	20-09	S.PP	2.130	0.973	1
181302	20-09	S.PP	1.845	0.973	1
181343	20-09	S.PP	1.544	0.973	1

Animal ID	Study #	Vaccine construct	ELISpot (log_{10} spots/10^6 PBMC)	Lung_reduction_R0	Protection_lung
181363	20-09	S.PP	1.740	0.973	1
191358	20-09	Sham	1.176	0.001	0
191372	20-09	Sham	1.301	0.001	0
T414	20-09	Sham	NA	0.481	0
T423	20-09	Sham	NA	0.001	0
T436	20-09	Sham	NA	0.000	0
T440	20-09	Sham	NA	0.001	0
T457	20-09	Sham	NA	0.001	0
T463	20-09	Sham	NA	0.001	0
15-072	20-14	S.PP	2.525	0.973	1
15-084	20-14	S.PP	2.130	0.973	1
15-086	20-14	S.PP	2.217	0.973	1
15-104	20-14	S.PP	1.699	0.973	1
15-108	20-14	S.PP	1.544	0.973	1
15-114	20-14	S.PP	1.176	0.001	0
15-115	20-14	S.PP	1.301	0.973	1
15-117	20-14	S.PP	1.602	0.973	1
15-118	20-14	S.PP	1.602	0.973	1
15-131	20-14	S.PP	2.000	0.973	1
15-139	20-14	S.PP	2.342	0.973	1
15-140	20-14	S.PP	1.000	0.152	0
15-143	20-14	S.PP	1.000	0.973	1
15-145	20-14	S.PP	1.301	0.973	1
15-146	20-14	Sham	1.000	0.002	0
15-148	20-14	Sham	1.000	0.547	0
15-157	20-14	S.PP	1.544	0.973	1
15-159	20-14	S.PP	1.740	0.973	1
15-161	20-14	S.PP	1.000	0.973	1
15-169	20-14	S.PP	1.000	0.973	1
15-171	20-14	S.PP	1.000	0.973	1
15-173	20-14	S.PP	1.929	0.973	1
15-175	20-14	Sham	1.000	0.001	0
15-179	20-14	Sham	1.000	0.499	0
15-183	20-14	Sham	1.000	0.194	0
T434F	20-14	Sham	NA	0.000	0
T435F	20-14	Sham	NA	0.001	0
T455M	20-14	Sham	NA	0.568	0
T458M	20-14	Sham	NA	0.000	0
T459M	20-14	Sham	NA	0.001	0

ELISpot: enzyme-linked immunospot assay; PBMC: peripheral blood mononuclear cells; NA: not available

Panel g and h

Animal ID	Study #	Vaccine construct	ELISpot (log_{10} spots/10^6 PBMC)	Nose_reduction_R0	Protection_nose
6418	20-07	Sham	1.000	0.000	0
6768	20-07	S.dCT	2.631	0.967	1
6770	20-07	tPA.WT.S	2.618	0.405	0
6855	20-07	tPA.S	2.380	0.967	1
6856	20-07	tPA.S	2.146	0.258	0
6905	20-07	S.dTM.PP	2.756	0.967	1
6906	20-07	S.dTM.PP	1.813	0.255	0
7028	20-07	tPA.S.PP	2.183	0.336	0
7056	20-07	tPA.S.PP	3.020	0.064	0
7123	20-07	Sham	NA	0.000	0
7130	20-07	Sham	NA	0.000	0
7145	20-07	Sham	NA	0.000	0
7154	20-07	Sham	NA	0.000	0
7157	20-07	Sham	NA	0.000	0
7245	20-07	Sham	NA	0.000	0
7261	20-07	Sham	NA	0.000	0
BM39	20-07	tPA.S	2.703	0.069	0
BO49	20-07	tPA.S	2.183	0.967	1
BO93	20-07	tPA.S.PP	2.720	0.967	1
BO95	20-07	tPA.S.PP	2.553	0.023	0
BP11	20-07	S.dCT	2.588	0.060	0
BS64	20-07	Sham	1.000	0.000	0
BS80	20-07	Sham	1.477	0.042	0
BS82	20-07	S	1.398	0.127	0
BV16	20-07	S	2.929	0.967	1
BV35	20-07	S.dCT	2.789	0.967	1
BV36	20-07	S.dCT	2.423	0.967	1
BV58	20-07	tPA.WT.S	2.789	0.489	0
BV79	20-07	tPA.WT.S	2.626	0.967	1
BV95	20-07	S.dTM.PP	2.505	0.967	1
BV96	20-07	S.dTM.PP	2.818	0.064	0
T781	20-07	S	1.398	0.331	0
T796	20-07	S	2.656	0.967	1
T799	20-07	tPA.WT.S	1.512	0.063	0
171170	20-09	S.dTM.PP	2.217	0.020	0
171219	20-09	Sham	1.000	0.000	0
171264	20-09	Sham	1.000	0.000	0
180662	20-09	S.PP	2.491	0.967	1
180689	20-09	S.PP	2.041	0.967	1
181267	20-09	S.PP	2.130	0.967	1
181302	20-09	S.PP	1.845	0.967	1
181343	20-09	S.PP	1.544	0.967	1

Animal ID	Study #	Vaccine construct	ELISpot (log_{10} spots/10^6 PBMC)	Nose_reduction_R0	Protection_nose
181363	20-09	S.PP	1.740	0.023	0
191358	20-09	Sham	1.176	0.000	0
191372	20-09	Sham	1.301	0.000	0
T414	20-09	Sham	NA	0.000	0
T423	20-09	Sham	NA	0.015	0
T436	20-09	Sham	NA	0.044	0
T440	20-09	Sham	NA	0.099	0
T457	20-09	Sham	NA	0.000	0
T463	20-09	Sham	NA	0.000	0
15-072	20-14	S.PP	2.525	0.967	1
15-084	20-14	S.PP	2.130	0.967	1
15-086	20-14	S.PP	2.217	0.967	1
15-104	20-14	S.PP	1.699	0.026	0
15-108	20-14	S.PP	1.544	0.050	0
15-114	20-14	S.PP	1.176	0.322	0
15-115	20-14	S.PP	1.301	0.967	1
15-117	20-14	S.PP	1.602	0.967	1
15-118	20-14	S.PP	1.602	0.118	0
15-131	20-14	S.PP	2.000	0.967	1
15-139	20-14	S.PP	2.342	0.967	1
15-140	20-14	S.PP	1.000	0.039	0
15-143	20-14	S.PP	1.000	0.967	1
15-145	20-14	S.PP	1.301	0.967	1
15-146	20-14	Sham	1.000	0.000	0
15-148	20-14	Sham	1.000	0.000	0
15-157	20-14	S.PP	1.544	0.967	1
15-159	20-14	S.PP	1.740	0.967	1
15-161	20-14	S.PP	1.000	0.967	1
15-169	20-14	S.PP	1.000	0.033	0
15-171	20-14	S.PP	1.000	0.967	1
15-173	20-14	S.PP	1.929	0.070	0
15-175	20-14	Sham	1.000	0.000	0
15-179	20-14	Sham	1.000	0.000	0
15-183	20-14	Sham	1.000	0.000	0
T434F	20-14	Sham	NA	0.152	0
T435F	20-14	Sham	NA	0.047	0
T455M	20-14	Sham	NA	0.174	0
T458M	20-14	Sham	NA	0.126	0
T459M	20-14	Sham	NA	0.000	0

ELISpot: enzyme-linked immunospot assay; PBMC: peripheral blood mononuclear cells; NA: not available

Supplementary Table 9: Source data file for Supplementary Figure 3

Panel a

Animal Group	Animal_ID	Study	Vaccine	Timepoint	Lung Viral Load (Log10 sgRNA copies/mL)
1	158274	6-month durability study	Sham	-1	1.70
1	158411	6-month durability study	Sham	-1	1.70
1	158422	6-month durability study	Sham	-1	1.70
1	1606400	6-month durability study	Sham	-1	1.70
1	1680940	6-month durability study	Sham	-1	1.70
1	1682980	6-month durability study	Sham	-1	1.70
1	1683040	6-month durability study	Sham	-1	1.70
1	158274	6-month durability study	Sham	1	3.14
1	158411	6-month durability study	Sham	1	1.70
1	158422	6-month durability study	Sham	1	3.64
1	1606400	6-month durability study	Sham	1	4.13
1	1680940	6-month durability study	Sham	1	3.74
1	1682980	6-month durability study	Sham	1	3.73
1	1683040	6-month durability study	Sham	1	3.78
1	158274	6-month durability study	Sham	2	3.97
1	158411	6-month durability study	Sham	2	1.70
1	158422	6-month durability study	Sham	2	4.02
1	1606400	6-month durability study	Sham	2	3.82
1	1680940	6-month durability study	Sham	2	1.70
1	1682980	6-month durability study	Sham	2	4.00
1	1683040	6-month durability study	Sham	2	5.32
1	158274	6-month durability study	Sham	4	4.43
1	158411	6-month durability study	Sham	4	2.66
1	158422	6-month durability study	Sham	4	2.70
1	1606400	6-month durability study	Sham	4	2.06
1	1680940	6-month durability study	Sham	4	1.70
1	1682980	6-month durability study	Sham	4	3.29
1	1683040	6-month durability study	Sham	4	3.48
1	158274	6-month durability study	Sham	7	1.70
1	158411	6-month durability study	Sham	7	2.31
1	158422	6-month durability study	Sham	7	1.70
1	1606400	6-month durability study	Sham	7	2.49
1	1680940	6-month durability study	Sham	7	1.70
1	1682980	6-month durability study	Sham	7	2.47
1	1683040	6-month durability study	Sham	7	2.57
1	158274	6-month durability study	Sham	10	1.70
1	158411	6-month durability study	Sham	10	1.70
1	158422	6-month durability study	Sham	10	1.70
1	1606400	6-month durability study	Sham	10	1.70
1	1680940	6-month durability study	Sham	10	1.70
1	1682980	6-month durability study	Sham	10	1.70

Animal Group	Animal_ID	Study	Vaccine	Timepoint	Lung Viral Load (Log10 sgRNA copies/mL)
1	1683040	6-month durability study	Sham	10	2.09
1	158274	6-month durability study	Sham	14	1.70
1	158411	6-month durability study	Sham	14	1.70
1	158422	6-month durability study	Sham	14	1.70
1	1606400	6-month durability study	Sham	14	1.70
1	1680940	6-month durability study	Sham	14	1.70
1	1682980	6-month durability study	Sham	14	1.70
1	1683040	6-month durability study	Sham	14	1.70

sgRNA: subgenomic RNA

Panel b

Animal Group	Animal_ID	Study	Vaccine	Timepoint	Lung Viral Load (Log10 sgRNA copies/mL)
2	158270	6-month durability study	1-dose 1x10^{11} vp Ad26.COV2.S	-1	1.70
2	158272	6-month durability study	1-dose 1x10^{11} vp Ad26.COV2.S	-1	1.70
2	158300	6-month durability study	1-dose 1x10^{11} vp Ad26.COV2.S	-1	1.70
2	1606460	6-month durability study	1-dose 1x10^{11} vp Ad26.COV2.S	-1	1.70
2	1681960	6-month durability study	1-dose 1x10^{11} vp Ad26.COV2.S	-1	1.70
2	1683000	6-month durability study	1-dose 1x10^{11} vp Ad26.COV2.S	-1	1.70
2	1683360	6-month durability study	1-dose 1x10^{11} vp Ad26.COV2.S	-1	1.70
2	158270	6-month durability study	1-dose 1x10^{11} vp Ad26.COV2.S	1	1.70
2	158272	6-month durability study	1-dose 1x10^{11} vp Ad26.COV2.S	1	1.70
2	158300	6-month durability study	1-dose 1x10^{11} vp Ad26.COV2.S	1	1.70
2	1606460	6-month durability study	1-dose 1x10^{11} vp Ad26.COV2.S	1	1.70
2	1681960	6-month durability study	1-dose 1x10^{11} vp Ad26.COV2.S	1	1.70
2	1683000	6-month durability study	1-dose 1x10^{11} vp Ad26.COV2.S	1	1.70
2	1683360	6-month durability study	1-dose 1x10^{11} vp Ad26.COV2.S	1	1.70
2	158270	6-month durability study	1-dose 1x10^{11} vp Ad26.COV2.S	2	1.70
2	158272	6-month durability study	1-dose 1x10^{11} vp Ad26.COV2.S	2	1.70
2	158300	6-month durability study	1-dose 1x10^{11} vp Ad26.COV2.S	2	1.70
2	1606460	6-month durability study	1-dose 1x10^{11} vp Ad26.COV2.S	2	1.70
2	1681960	6-month durability study	1-dose 1x10^{11} vp Ad26.COV2.S	2	1.70
2	1683000	6-month durability study	1-dose 1x10^{11} vp Ad26.COV2.S	2	1.70
2	1683360	6-month durability study	1-dose 1x10^{11} vp Ad26.COV2.S	2	1.70
2	158270	6-month durability study	1-dose 1x10^{11} vp Ad26.COV2.S	4	1.70
2	158272	6-month durability study	1-dose 1x10^{11} vp Ad26.COV2.S	4	1.70
2	158300	6-month durability study	1-dose 1x10^{11} vp Ad26.COV2.S	4	1.70
2	1606460	6-month durability study	1-dose 1x10^{11} vp Ad26.COV2.S	4	1.70
2	1681960	6-month durability study	1-dose 1x10^{11} vp Ad26.COV2.S	4	1.70
2	1683000	6-month durability study	1-dose 1x10^{11} vp Ad26.COV2.S	4	1.70

Animal Group	Animal_ID	Study	Vaccine	Timepoint	Lung Viral Load (Log10 sgRNA copies/mL)
2	1683360	6-month durability study	1-dose $1x10^{11}$ vp Ad26.COV2.S	4	1.70
2	158270	6-month durability study	1-dose $1x10^{11}$ vp Ad26.COV2.S	7	1.70
2	158272	6-month durability study	1-dose $1x10^{11}$ vp Ad26.COV2.S	7	1.70
2	158300	6-month durability study	1-dose $1x10^{11}$ vp Ad26.COV2.S	7	1.70
2	1606460	6-month durability study	1-dose $1x10^{11}$ vp Ad26.COV2.S	7	1.70
2	1681960	6-month durability study	1-dose $1x10^{11}$ vp Ad26.COV2.S	7	1.70
2	1683000	6-month durability study	1-dose $1x10^{11}$ vp Ad26.COV2.S	7	1.70
2	1683360	6-month durability study	1-dose $1x10^{11}$ vp Ad26.COV2.S	7	1.70
2	158270	6-month durability study	1-dose $1x10^{11}$ vp Ad26.COV2.S	10	1.70
2	158272	6-month durability study	1-dose $1x10^{11}$ vp Ad26.COV2.S	10	1.70
2	158300	6-month durability study	1-dose $1x10^{11}$ vp Ad26.COV2.S	10	1.70
2	1606460	6-month durability study	1-dose $1x10^{11}$ vp Ad26.COV2.S	10	1.70
2	1681960	6-month durability study	1-dose $1x10^{11}$ vp Ad26.COV2.S	10	1.70
2	1683000	6-month durability study	1-dose $1x10^{11}$ vp Ad26.COV2.S	10	1.70
2	1683360	6-month durability study	1-dose $1x10^{11}$ vp Ad26.COV2.S	10	1.70
2	158270	6-month durability study	1-dose $1x10^{11}$ vp Ad26.COV2.S	14	1.70
2	158272	6-month durability study	1-dose $1x10^{11}$ vp Ad26.COV2.S	14	1.70
2	158300	6-month durability study	1-dose $1x10^{11}$ vp Ad26.COV2.S	14	1.70
2	1606460	6-month durability study	1-dose $1x10^{11}$ vp Ad26.COV2.S	14	1.70
2	1681960	6-month durability study	1-dose $1x10^{11}$ vp Ad26.COV2.S	14	1.70
2	1683000	6-month durability study	1-dose $1x10^{11}$ vp Ad26.COV2.S	14	1.70
2	1683360	6-month durability study	1-dose $1x10^{11}$ vp Ad26.COV2.S	14	1.70

sgRNA: subgenomic RNA; vp: viral particles

Panel c

Animal Group	Animal_ID	Study	Vaccine	Timepoint	Lung Viral Load (Log10 sgRNA copies/mL)
3	1506260	6-month durability study	1-dose $5x10^{10}$ vp Ad26.COV2.S	-1	1.70
3	158284	6-month durability study	1-dose $5x10^{10}$ vp Ad26.COV2.S	-1	1.70
3	158298	6-month durability study	1-dose $5x10^{10}$ vp Ad26.COV2.S	-1	1.70
3	1680780	6-month durability study	1-dose $5x10^{10}$ vp Ad26.COV2.S	-1	1.70
3	1682300	6-month durability study	1-dose $5x10^{10}$ vp Ad26.COV2.S	-1	1.70
3	1683160	6-month durability study	1-dose $5x10^{10}$ vp Ad26.COV2.S	-1	1.70
3	1801551	6-month durability study	1-dose $5x10^{10}$ vp Ad26.COV2.S	-1	1.70
3	1506260	6-month durability study	1-dose $5x10^{10}$ vp Ad26.COV2.S	1	1.70
3	158284	6-month durability study	1-dose $5x10^{10}$ vp Ad26.COV2.S	1	3.36
3	158298	6-month durability study	1-dose $5x10^{10}$ vp Ad26.COV2.S	1	1.70
3	1680780	6-month durability study	1-dose $5x10^{10}$ vp Ad26.COV2.S	1	1.70
3	1682300	6-month durability study	1-dose $5x10^{10}$ vp Ad26.COV2.S	1	1.70
3	1683160	6-month durability study	1-dose $5x10^{10}$ vp Ad26.COV2.S	1	1.70
3	1801551	6-month durability study	1-dose $5x10^{10}$ vp Ad26.COV2.S	1	1.70
3	1506260	6-month durability study	1-dose $5x10^{10}$ vp Ad26.COV2.S	2	1.70
3	158284	6-month durability study	1-dose $5x10^{10}$ vp Ad26.COV2.S	2	3.23
3	158298	6-month durability study	1-dose $5x10^{10}$ vp Ad26.COV2.S	2	1.70
3	1680780	6-month durability study	1-dose $5x10^{10}$ vp Ad26.COV2.S	2	1.70
3	1682300	6-month durability study	1-dose $5x10^{10}$ vp Ad26.COV2.S	2	1.70
3	1683160	6-month durability study	1-dose $5x10^{10}$ vp Ad26.COV2.S	2	1.70
3	1801551	6-month durability study	1-dose $5x10^{10}$ vp Ad26.COV2.S	2	1.70
3	1506260	6-month durability study	1-dose $5x10^{10}$ vp Ad26.COV2.S	4	1.70
3	158284	6-month durability study	1-dose $5x10^{10}$ vp Ad26.COV2.S	4	1.70
3	158298	6-month durability study	1-dose $5x10^{10}$ vp Ad26.COV2.S	4	1.70
3	1680780	6-month durability study	1-dose $5x10^{10}$ vp Ad26.COV2.S	4	1.70
3	1682300	6-month durability study	1-dose $5x10^{10}$ vp Ad26.COV2.S	4	1.70
3	1683160	6-month durability study	1-dose $5x10^{10}$ vp Ad26.COV2.S	4	1.70

Animal Group	Animal_ID	Study	Vaccine	Timepoint	Lung Viral Load (Log10 sgRNA copies/mL)
3	1801551	6-month durability study	1-dose $5x10^{10}$ vp Ad26.COV2.S	4	1.70
3	1506260	6-month durability study	1-dose $5x10^{10}$ vp Ad26.COV2.S	7	1.70
3	158284	6-month durability study	1-dose $5x10^{10}$ vp Ad26.COV2.S	7	1.70
3	158298	6-month durability study	1-dose $5x10^{10}$ vp Ad26.COV2.S	7	1.70
3	1680780	6-month durability study	1-dose $5x10^{10}$ vp Ad26.COV2.S	7	1.70
3	1682300	6-month durability study	1-dose $5x10^{10}$ vp Ad26.COV2.S	7	1.70
3	1683160	6-month durability study	1-dose $5x10^{10}$ vp Ad26.COV2.S	7	1.70
3	1801551	6-month durability study	1-dose $5x10^{10}$ vp Ad26.COV2.S	7	1.70
3	1506260	6-month durability study	1-dose $5x10^{10}$ vp Ad26.COV2.S	10	1.70
3	158284	6-month durability study	1-dose $5x10^{10}$ vp Ad26.COV2.S	10	1.70
3	158298	6-month durability study	1-dose $5x10^{10}$ vp Ad26.COV2.S	10	1.70
3	1680780	6-month durability study	1-dose $5x10^{10}$ vp Ad26.COV2.S	10	1.70
3	1682300	6-month durability study	1-dose $5x10^{10}$ vp Ad26.COV2.S	10	1.70
3	1683160	6-month durability study	1-dose $5x10^{10}$ vp Ad26.COV2.S	10	1.70
3	1801551	6-month durability study	1-dose $5x10^{10}$ vp Ad26.COV2.S	10	1.70
3	1506260	6-month durability study	1-dose $5x10^{10}$ vp Ad26.COV2.S	14	1.70
3	158284	6-month durability study	1-dose $5x10^{10}$ vp Ad26.COV2.S	14	1.70
3	158298	6-month durability study	1-dose $5x10^{10}$ vp Ad26.COV2.S	14	1.70
3	1680780	6-month durability study	1-dose $5x10^{10}$ vp Ad26.COV2.S	14	1.70
3	1682300	6-month durability study	1-dose $5x10^{10}$ vp Ad26.COV2.S	14	1.70
3	1683160	6-month durability study	1-dose $5x10^{10}$ vp Ad26.COV2.S	14	1.70
3	1801551	6-month durability study	1-dose $5x10^{10}$ vp Ad26.COV2.S	14	1.70

sgRNA: subgenomic RNA; vp: viral particles

Panel d

Animal Group	Animal_ID	Study	Vaccine	Timepoint	Lung Viral Load (Log10 sgRNA copies/mL)
4	158278	6-month durability study	2-dose $5x10^{10}$ vp Ad26.COV2.S (0-4)	-1	1.70
4	158282	6-month durability study	2-dose $5x10^{10}$ vp Ad26.COV2.S (0-4)	-1	1.70
4	1600560	6-month durability study	2-dose $5x10^{10}$ vp Ad26.COV2.S (0-4)	-1	1.70
4	1680120	6-month durability study	2-dose $5x10^{10}$ vp Ad26.COV2.S (0-4)	-1	1.70
4	1683440	6-month durability study	2-dose $5x10^{10}$ vp Ad26.COV2.S (0-4)	-1	1.70
4	1701220	6-month durability study	2-dose $5x10^{10}$ vp Ad26.COV2.S (0-4)	-1	1.70
4	1706171	6-month durability study	2-dose $5x10^{10}$ vp Ad26.COV2.S (0-4)	-1	1.70
4	158278	6-month durability study	2-dose $5x10^{10}$ vp Ad26.COV2.S (0-4)	1	1.70
4	158282	6-month durability study	2-dose $5x10^{10}$ vp Ad26.COV2.S (0-4)	1	1.70
4	1600560	6-month durability study	2-dose $5x10^{10}$ vp Ad26.COV2.S (0-4)	1	1.70
4	1680120	6-month durability study	2-dose $5x10^{10}$ vp Ad26.COV2.S (0-4)	1	1.70
4	1683440	6-month durability study	2-dose $5x10^{10}$ vp Ad26.COV2.S (0-4)	1	1.70
4	1701220	6-month durability study	2-dose $5x10^{10}$ vp Ad26.COV2.S (0-4)	1	1.70
4	1706171	6-month durability study	2-dose $5x10^{10}$ vp Ad26.COV2.S (0-4)	1	1.70
4	158278	6-month durability study	2-dose $5x10^{10}$ vp Ad26.COV2.S (0-4)	2	1.70
4	158282	6-month durability study	2-dose $5x10^{10}$ vp Ad26.COV2.S (0-4)	2	1.70
4	1600560	6-month durability study	2-dose $5x10^{10}$ vp Ad26.COV2.S (0-4)	2	1.70
4	1680120	6-month durability study	2-dose $5x10^{10}$ vp Ad26.COV2.S (0-4)	2	1.70
4	1683440	6-month durability study	2-dose $5x10^{10}$ vp Ad26.COV2.S (0-4)	2	1.70
4	1701220	6-month durability study	2-dose $5x10^{10}$ vp Ad26.COV2.S (0-4)	2	1.70
4	1706171	6-month durability study	2-dose $5x10^{10}$ vp Ad26.COV2.S (0-4)	2	1.70
4	158278	6-month durability study	2-dose $5x10^{10}$ vp Ad26.COV2.S (0-4)	4	1.70
4	158282	6-month durability study	2-dose $5x10^{10}$ vp Ad26.COV2.S (0-4)	4	1.70
4	1600560	6-month durability study	2-dose $5x10^{10}$ vp Ad26.COV2.S (0-4)	4	1.70
4	1680120	6-month durability study	2-dose $5x10^{10}$ vp Ad26.COV2.S (0-4)	4	1.70
4	1683440	6-month durability study	2-dose $5x10^{10}$ vp Ad26.COV2.S (0-4)	4	1.70
4	1701220	6-month durability study	2-dose $5x10^{10}$ vp Ad26.COV2.S (0-4)	4	1.70

Animal Group	Animal_ID	Study	Vaccine	Timepoint	Lung Viral Load (Log10 sgRNA copies/mL)
4	1706171	6-month durability study	2-dose $5x10^{10}$ vp Ad26.COV2.S (0-4)	4	1.70
4	158278	6-month durability study	2-dose $5x10^{10}$ vp Ad26.COV2.S (0-4)	7	1.70
4	158282	6-month durability study	2-dose $5x10^{10}$ vp Ad26.COV2.S (0-4)	7	1.70
4	1600560	6-month durability study	2-dose $5x10^{10}$ vp Ad26.COV2.S (0-4)	7	1.70
4	1680120	6-month durability study	2-dose $5x10^{10}$ vp Ad26.COV2.S (0-4)	7	1.70
4	1683440	6-month durability study	2-dose $5x10^{10}$ vp Ad26.COV2.S (0-4)	7	1.70
4	1701220	6-month durability study	2-dose $5x10^{10}$ vp Ad26.COV2.S (0-4)	7	1.70
4	1706171	6-month durability study	2-dose $5x10^{10}$ vp Ad26.COV2.S (0-4)	7	1.70
4	158278	6-month durability study	2-dose $5x10^{10}$ vp Ad26.COV2.S (0-4)	10	1.70
4	158282	6-month durability study	2-dose $5x10^{10}$ vp Ad26.COV2.S (0-4)	10	1.70
4	1600560	6-month durability study	2-dose $5x10^{10}$ vp Ad26.COV2.S (0-4)	10	1.70
4	1680120	6-month durability study	2-dose $5x10^{10}$ vp Ad26.COV2.S (0-4)	10	1.70
4	1683440	6-month durability study	2-dose $5x10^{10}$ vp Ad26.COV2.S (0-4)	10	1.70
4	1701220	6-month durability study	2-dose $5x10^{10}$ vp Ad26.COV2.S (0-4)	10	1.70
4	1706171	6-month durability study	2-dose $5x10^{10}$ vp Ad26.COV2.S (0-4)	10	1.70
4	158278	6-month durability study	2-dose $5x10^{10}$ vp Ad26.COV2.S (0-4)	14	1.70
4	158282	6-month durability study	2-dose $5x10^{10}$ vp Ad26.COV2.S (0-4)	14	1.70
4	1600560	6-month durability study	2-dose $5x10^{10}$ vp Ad26.COV2.S (0-4)	14	1.70
4	1680120	6-month durability study	2-dose $5x10^{10}$ vp Ad26.COV2.S (0-4)	14	1.70
4	1683440	6-month durability study	2-dose $5x10^{10}$ vp Ad26.COV2.S (0-4)	14	1.70
4	1701220	6-month durability study	2-dose $5x10^{10}$ vp Ad26.COV2.S (0-4)	14	1.70
4	1706171	6-month durability study	2-dose $5x10^{10}$ vp Ad26.COV2.S (0-4)	14	1.70

sgRNA: subgenomic RNA; vp: viral particles

Panel e

Animal Group	Animal_ID	Study	Vaccine	Timepoint	Lung Viral Load (Log10 sgRNA copies/mL)
5	158280	6-month durability study	2-dose $5x10^{10}$ vp Ad26.COV2.S (0-8)	-1	1.70
5	1606520	6-month durability study	2-dose $5x10^{10}$ vp Ad26.COV2.S (0-8)	-1	1.70
5	1680260	6-month durability study	2-dose $5x10^{10}$ vp Ad26.COV2.S (0-8)	-1	1.70
5	1682671	6-month durability study	2-dose $5x10^{10}$ vp Ad26.COV2.S (0-8)	-1	1.70
5	1683140	6-month durability study	2-dose $5x10^{10}$ vp Ad26.COV2.S (0-8)	-1	1.70
5	1683520	6-month durability study	2-dose $5x10^{10}$ vp Ad26.COV2.S (0-8)	-1	1.70
5	1683640	6-month durability study	2-dose $5x10^{10}$ vp Ad26.COV2.S (0-8)	-1	1.70
5	158280	6-month durability study	2-dose $5x10^{10}$ vp Ad26.COV2.S (0-8)	1	1.70
5	1606520	6-month durability study	2-dose $5x10^{10}$ vp Ad26.COV2.S (0-8)	1	1.70
5	1680260	6-month durability study	2-dose $5x10^{10}$ vp Ad26.COV2.S (0-8)	1	1.70
5	1682671	6-month durability study	2-dose $5x10^{10}$ vp Ad26.COV2.S (0-8)	1	3.85
5	1683140	6-month durability study	2-dose $5x10^{10}$ vp Ad26.COV2.S (0-8)	1	1.70
5	1683520	6-month durability study	2-dose $5x10^{10}$ vp Ad26.COV2.S (0-8)	1	1.70
5	1683640	6-month durability study	2-dose $5x10^{10}$ vp Ad26.COV2.S (0-8)	1	1.70
5	158280	6-month durability study	2-dose $5x10^{10}$ vp Ad26.COV2.S (0-8)	2	1.70
5	1606520	6-month durability study	2-dose $5x10^{10}$ vp Ad26.COV2.S (0-8)	2	1.70
5	1680260	6-month durability study	2-dose $5x10^{10}$ vp Ad26.COV2.S (0-8)	2	1.70
5	1682671	6-month durability study	2-dose $5x10^{10}$ vp Ad26.COV2.S (0-8)	2	3.87
5	1683140	6-month durability study	2-dose $5x10^{10}$ vp Ad26.COV2.S (0-8)	2	1.70
5	1683520	6-month durability study	2-dose $5x10^{10}$ vp Ad26.COV2.S (0-8)	2	3.32
5	1683640	6-month durability study	2-dose $5x10^{10}$ vp Ad26.COV2.S (0-8)	2	1.70
5	158280	6-month durability study	2-dose $5x10^{10}$ vp Ad26.COV2.S (0-8)	4	1.70
5	1606520	6-month durability study	2-dose $5x10^{10}$ vp Ad26.COV2.S (0-8)	4	1.70
5	1680260	6-month durability study	2-dose $5x10^{10}$ vp Ad26.COV2.S (0-8)	4	1.70
5	1682671	6-month durability study	2-dose $5x10^{10}$ vp Ad26.COV2.S (0-8)	4	3.14
5	1683140	6-month durability study	2-dose $5x10^{10}$ vp Ad26.COV2.S (0-8)	4	1.70
5	1683520	6-month durability study	2-dose $5x10^{10}$ vp Ad26.COV2.S (0-8)	4	1.70

Animal Group	Animal_ID	Study	Vaccine	Timepoint	Lung Viral Load (Log10 sgRNA copies/mL)
5	1683640	6-month durability study	2-dose $5x10^{10}$ vp Ad26.COV2.S (0-8)	4	1.70
5	158280	6-month durability study	2-dose $5x10^{10}$ vp Ad26.COV2.S (0-8)	7	1.70
5	1606520	6-month durability study	2-dose $5x10^{10}$ vp Ad26.COV2.S (0-8)	7	1.70
5	1680260	6-month durability study	2-dose $5x10^{10}$ vp Ad26.COV2.S (0-8)	7	1.70
5	1682671	6-month durability study	2-dose $5x10^{10}$ vp Ad26.COV2.S (0-8)	7	1.70
5	1683140	6-month durability study	2-dose $5x10^{10}$ vp Ad26.COV2.S (0-8)	7	1.70
5	1683520	6-month durability study	2-dose $5x10^{10}$ vp Ad26.COV2.S (0-8)	7	1.70
5	1683640	6-month durability study	2-dose $5x10^{10}$ vp Ad26.COV2.S (0-8)	7	1.95
5	158280	6-month durability study	2-dose $5x10^{10}$ vp Ad26.COV2.S (0-8)	10	1.70
5	1606520	6-month durability study	2-dose $5x10^{10}$ vp Ad26.COV2.S (0-8)	10	1.70
5	1680260	6-month durability study	2-dose $5x10^{10}$ vp Ad26.COV2.S (0-8)	10	1.70
5	1682671	6-month durability study	2-dose $5x10^{10}$ vp Ad26.COV2.S (0-8)	10	1.70
5	1683140	6-month durability study	2-dose $5x10^{10}$ vp Ad26.COV2.S (0-8)	10	1.70
5	1683520	6-month durability study	2-dose $5x10^{10}$ vp Ad26.COV2.S (0-8)	10	1.70
5	1683640	6-month durability study	2-dose $5x10^{10}$ vp Ad26.COV2.S (0-8)	10	1.70
5	158280	6-month durability study	2-dose $5x10^{10}$ vp Ad26.COV2.S (0-8)	14	1.70
5	1606520	6-month durability study	2-dose $5x10^{10}$ vp Ad26.COV2.S (0-8)	14	1.70
5	1680260	6-month durability study	2-dose $5x10^{10}$ vp Ad26.COV2.S (0-8)	14	1.70
5	1682671	6-month durability study	2-dose $5x10^{10}$ vp Ad26.COV2.S (0-8)	14	1.70
5	1683140	6-month durability study	2-dose $5x10^{10}$ vp Ad26.COV2.S (0-8)	14	1.70
5	1683520	6-month durability study	2-dose $5x10^{10}$ vp Ad26.COV2.S (0-8)	14	1.70
5	1683640	6-month durability study	2-dose $5x10^{10}$ vp Ad26.COV2.S (0-8)	14	1.70

sgRNA: subgenomic RNA; vp: viral particles

Panel f

Animal Group	Animal_ID	Study	Vaccine	No. sampling days with viral load>LOD (Lung)
1	158274	6-month durability study	Sham	3
1	158411	6-month durability study	Sham	2
1	158422	6-month durability study	Sham	3
1	1606400	6-month durability study	Sham	3
1	1680940	6-month durability study	Sham	1
1	1682980	6-month durability study	Sham	4
1	1683040	6-month durability study	Sham	4
2	158270	6-month durability study	1-dose $1x10^{11}$ vp Ad26.COV2.S	0
2	158272	6-month durability study	1-dose $1x10^{11}$ vp Ad26.COV2.S	0
2	158300	6-month durability study	1-dose $1x10^{11}$ vp Ad26.COV2.S	0
2	1606460	6-month durability study	1-dose $1x10^{11}$ vp Ad26.COV2.S	0
2	1681960	6-month durability study	1-dose $1x10^{11}$ vp Ad26.COV2.S	0
2	1683000	6-month durability study	1-dose $1x10^{11}$ vp Ad26.COV2.S	0
2	1683360	6-month durability study	1-dose $1x10^{11}$ vp Ad26.COV2.S	0
3	1506260	6-month durability study	1-dose $5x10^{10}$ vp Ad26.COV2.S	0
3	158284	6-month durability study	1-dose $5x10^{10}$ vp Ad26.COV2.S	2
3	158298	6-month durability study	1-dose $5x10^{10}$ vp Ad26.COV2.S	0
3	1680780	6-month durability study	1-dose $5x10^{10}$ vp Ad26.COV2.S	0
3	1682300	6-month durability study	1-dose $5x10^{10}$ vp Ad26.COV2.S	0
3	1683160	6-month durability study	1-dose $5x10^{10}$ vp Ad26.COV2.S	0
3	1801551	6-month durability study	1-dose $5x10^{10}$ vp Ad26.COV2.S	0
4	158278	6-month durability study	2-dose $5x10^{10}$ vp Ad26.COV2.S (0-4)	0
4	158282	6-month durability study	2-dose $5x10^{10}$ vp Ad26.COV2.S (0-4)	0
4	1600560	6-month durability study	2-dose $5x10^{10}$ vp Ad26.COV2.S (0-4)	0
4	1680120	6-month durability study	2-dose $5x10^{10}$ vp Ad26.COV2.S (0-4)	0
4	1683440	6-month durability study	2-dose $5x10^{10}$ vp Ad26.COV2.S (0-4)	0
4	1701220	6-month durability study	2-dose $5x10^{10}$ vp Ad26.COV2.S (0-4)	0
4	1706171	6-month durability study	2-dose $5x10^{10}$ vp Ad26.COV2.S (0-4)	0
5	158280	6-month durability study	2-dose $5x10^{10}$ vp Ad26.COV2.S (0-8)	0
5	1606520	6-month durability study	2-dose $5x10^{10}$ vp Ad26.COV2.S (0-8)	0

Animal Group	Animal_ID	Study	Vaccine	No. sampling days with viral load>LOD (Lung)
5	1680260	6-month durability study	2-dose $5x10^{10}$ vp Ad26.COV2.S (0-8)	0
5	1682671	6-month durability study	2-dose $5x10^{10}$ vp Ad26.COV2.S (0-8)	3
5	1683140	6-month durability study	2-dose $5x10^{10}$ vp Ad26.COV2.S (0-8)	0
5	1683520	6-month durability study	2-dose $5x10^{10}$ vp Ad26.COV2.S (0-8)	1
5	1683640	6-month durability study	2-dose $5x10^{10}$ vp Ad26.COV2.S (0-8)	0

LOD: limit of detection; vp: viral particles

Supplementary Table 10: Source data file for Supplementary Figure 4

Panel a

Animal Group	Animal_ID	Study	Vaccine	Timepoint	Nose Viral Load (Log10 sgRNA copies/swab)
1	158274	6-month durability study	Sham	-1	1.70
1	158411	6-month durability study	Sham	-1	1.70
1	158422	6-month durability study	Sham	-1	1.70
1	1606400	6-month durability study	Sham	-1	1.70
1	1680940	6-month durability study	Sham	-1	1.70
1	1682980	6-month durability study	Sham	-1	1.70
1	1683040	6-month durability study	Sham	-1	1.70
1	158274	6-month durability study	Sham	1	2.91
1	158411	6-month durability study	Sham	1	3.93
1	158422	6-month durability study	Sham	1	2.58
1	1606400	6-month durability study	Sham	1	3.95
1	1680940	6-month durability study	Sham	1	5.43
1	1682980	6-month durability study	Sham	1	3.66
1	1683040	6-month durability study	Sham	1	4.53
1	158274	6-month durability study	Sham	2	6.11
1	158411	6-month durability study	Sham	2	5.83
1	158422	6-month durability study	Sham	2	4.47
1	1606400	6-month durability study	Sham	2	5.04
1	1680940	6-month durability study	Sham	2	5.83
1	1682980	6-month durability study	Sham	2	5.64
1	1683040	6-month durability study	Sham	2	6.72
1	158274	6-month durability study	Sham	4	5.28
1	158411	6-month durability study	Sham	4	4.20
1	158422	6-month durability study	Sham	4	4.24
1	1606400	6-month durability study	Sham	4	3.68
1	1680940	6-month durability study	Sham	4	3.82
1	1682980	6-month durability study	Sham	4	4.22
1	1683040	6-month durability study	Sham	4	4.28
1	158274	6-month durability study	Sham	7	2.76
1	158411	6-month durability study	Sham	7	2.56
1	158422	6-month durability study	Sham	7	3.20
1	1606400	6-month durability study	Sham	7	3.85
1	1680940	6-month durability study	Sham	7	3.89
1	1682980	6-month durability study	Sham	7	3.13
1	1683040	6-month durability study	Sham	7	1.70
1	158274	6-month durability study	Sham	10	1.70
1	158411	6-month durability study	Sham	10	1.70
1	158422	6-month durability study	Sham	10	1.70
1	1606400	6-month durability study	Sham	10	3.94
1	1680940	6-month durability study	Sham	10	4.18
1	1682980	6-month durability study	Sham	10	1.70

Animal Group	Animal_ID	Study	Vaccine	Timepoint	Nose Viral Load (Log10 sgRNA copies/swab)
1	1683040	6-month durability study	Sham	10	3.92
1	158274	6-month durability study	Sham	14	1.70
1	158411	6-month durability study	Sham	14	1.70
1	158422	6-month durability study	Sham	14	1.70
1	1606400	6-month durability study	Sham	14	3.27
1	1680940	6-month durability study	Sham	14	3.39
1	1682980	6-month durability study	Sham	14	1.70
1	1683040	6-month durability study	Sham	14	1.70

sgRNA: subgenomic RNA

Panel b

Animal Group	Animal_ID	Study	Vaccine	Timepoint	Nose Viral Load (Log10 sgRNA copies/swab)
2	158270	6-month durability study	1-dose $1x10^{11}$ vp Ad26.COV2.S	-1	1.70
2	158272	6-month durability study	1-dose $1x10^{11}$ vp Ad26.COV2.S	-1	1.70
2	158300	6-month durability study	1-dose $1x10^{11}$ vp Ad26.COV2.S	-1	1.70
2	1606460	6-month durability study	1-dose $1x10^{11}$ vp Ad26.COV2.S	-1	1.70
2	1681960	6-month durability study	1-dose $1x10^{11}$ vp Ad26.COV2.S	-1	1.70
2	1683000	6-month durability study	1-dose $1x10^{11}$ vp Ad26.COV2.S	-1	1.70
2	1683360	6-month durability study	1-dose $1x10^{11}$ vp Ad26.COV2.S	-1	1.70
2	158270	6-month durability study	1-dose $1x10^{11}$ vp Ad26.COV2.S	1	1.70
2	158272	6-month durability study	1-dose $1x10^{11}$ vp Ad26.COV2.S	1	1.70
2	158300	6-month durability study	1-dose $1x10^{11}$ vp Ad26.COV2.S	1	4.95
2	1606460	6-month durability study	1-dose $1x10^{11}$ vp Ad26.COV2.S	1	4.25
2	1681960	6-month durability study	1-dose $1x10^{11}$ vp Ad26.COV2.S	1	3.80
2	1683000	6-month durability study	1-dose $1x10^{11}$ vp Ad26.COV2.S	1	3.78
2	1683360	6-month durability study	1-dose $1x10^{11}$ vp Ad26.COV2.S	1	3.42
2	158270	6-month durability study	1-dose $1x10^{11}$ vp Ad26.COV2.S	2	2.86
2	158272	6-month durability study	1-dose $1x10^{11}$ vp Ad26.COV2.S	2	1.70
2	158300	6-month durability study	1-dose $1x10^{11}$ vp Ad26.COV2.S	2	5.72
2	1606460	6-month durability study	1-dose $1x10^{11}$ vp Ad26.COV2.S	2	5.50
2	1681960	6-month durability study	1-dose $1x10^{11}$ vp Ad26.COV2.S	2	3.71
2	1683000	6-month durability study	1-dose $1x10^{11}$ vp Ad26.COV2.S	2	5.58
2	1683360	6-month durability study	1-dose $1x10^{11}$ vp Ad26.COV2.S	2	4.96
2	158270	6-month durability study	1-dose $1x10^{11}$ vp Ad26.COV2.S	4	4.32
2	158272	6-month durability study	1-dose $1x10^{11}$ vp Ad26.COV2.S	4	1.70
2	158300	6-month durability study	1-dose $1x10^{11}$ vp Ad26.COV2.S	4	3.67
2	1606460	6-month durability study	1-dose $1x10^{11}$ vp Ad26.COV2.S	4	3.84
2	1681960	6-month durability study	1-dose $1x10^{11}$ vp Ad26.COV2.S	4	5.14
2	1683000	6-month durability study	1-dose $1x10^{11}$ vp Ad26.COV2.S	4	1.70

Animal Group	Animal_ID	Study	Vaccine	Timepoint	Nose Viral Load (Log10 sgRNA copies/swab)
2	1683360	6-month durability study	1-dose 1x10^{11} vp Ad26.COV2.S	4	4.20
2	158270	6-month durability study	1-dose 1x10^{11} vp Ad26.COV2.S	7	1.70
2	158272	6-month durability study	1-dose 1x10^{11} vp Ad26.COV2.S	7	1.70
2	158300	6-month durability study	1-dose 1x10^{11} vp Ad26.COV2.S	7	1.70
2	1606460	6-month durability study	1-dose 1x10^{11} vp Ad26.COV2.S	7	1.70
2	1681960	6-month durability study	1-dose 1x10^{11} vp Ad26.COV2.S	7	5.19
2	1683000	6-month durability study	1-dose 1x10^{11} vp Ad26.COV2.S	7	2.88
2	1683360	6-month durability study	1-dose 1x10^{11} vp Ad26.COV2.S	7	4.76
2	158270	6-month durability study	1-dose 1x10^{11} vp Ad26.COV2.S	10	1.70
2	158272	6-month durability study	1-dose 1x10^{11} vp Ad26.COV2.S	10	1.70
2	158300	6-month durability study	1-dose 1x10^{11} vp Ad26.COV2.S	10	1.70
2	1606460	6-month durability study	1-dose 1x10^{11} vp Ad26.COV2.S	10	1.70
2	1681960	6-month durability study	1-dose 1x10^{11} vp Ad26.COV2.S	10	1.70
2	1683000	6-month durability study	1-dose 1x10^{11} vp Ad26.COV2.S	10	1.70
2	1683360	6-month durability study	1-dose 1x10^{11} vp Ad26.COV2.S	10	1.70
2	158270	6-month durability study	1-dose 1x10^{11} vp Ad26.COV2.S	14	1.70
2	158272	6-month durability study	1-dose 1x10^{11} vp Ad26.COV2.S	14	1.70
2	158300	6-month durability study	1-dose 1x10^{11} vp Ad26.COV2.S	14	1.70
2	1606460	6-month durability study	1-dose 1x10^{11} vp Ad26.COV2.S	14	1.70
2	1681960	6-month durability study	1-dose 1x10^{11} vp Ad26.COV2.S	14	1.70
2	1683000	6-month durability study	1-dose 1x10^{11} vp Ad26.COV2.S	14	1.70
2	1683360	6-month durability study	1-dose 1x10^{11} vp Ad26.COV2.S	14	1.70

sgRNA: subgenomic RNA; vp: viral particles

Panel c

Animal Group	Animal_ID	Study	Vaccine	Timepoint	Nose Viral Load (Log10 sgRNA copies/swab)
3	1506260	6-month durability study	1-dose $5x10^{10}$ vp Ad26.COV2.S	-1	1.70
3	158284	6-month durability study	1-dose $5x10^{10}$ vp Ad26.COV2.S	-1	1.70
3	158298	6-month durability study	1-dose $5x10^{10}$ vp Ad26.COV2.S	-1	1.70
3	1680780	6-month durability study	1-dose $5x10^{10}$ vp Ad26.COV2.S	-1	1.70
3	1682300	6-month durability study	1-dose $5x10^{10}$ vp Ad26.COV2.S	-1	1.70
3	1683160	6-month durability study	1-dose $5x10^{10}$ vp Ad26.COV2.S	-1	1.70
3	1801551	6-month durability study	1-dose $5x10^{10}$ vp Ad26.COV2.S	-1	1.70
3	1506260	6-month durability study	1-dose $5x10^{10}$ vp Ad26.COV2.S	1	4.09
3	158284	6-month durability study	1-dose $5x10^{10}$ vp Ad26.COV2.S	1	4.70
3	158298	6-month durability study	1-dose $5x10^{10}$ vp Ad26.COV2.S	1	1.81
3	1680780	6-month durability study	1-dose $5x10^{10}$ vp Ad26.COV2.S	1	4.38
3	1682300	6-month durability study	1-dose $5x10^{10}$ vp Ad26.COV2.S	1	4.84
3	1683160	6-month durability study	1-dose $5x10^{10}$ vp Ad26.COV2.S	1	3.26
3	1801551	6-month durability study	1-dose $5x10^{10}$ vp Ad26.COV2.S	1	4.71
3	1506260	6-month durability study	1-dose $5x10^{10}$ vp Ad26.COV2.S	2	5.78
3	158284	6-month durability study	1-dose $5x10^{10}$ vp Ad26.COV2.S	2	6.27
3	158298	6-month durability study	1-dose $5x10^{10}$ vp Ad26.COV2.S	2	1.73
3	1680780	6-month durability study	1-dose $5x10^{10}$ vp Ad26.COV2.S	2	6.18
3	1682300	6-month durability study	1-dose $5x10^{10}$ vp Ad26.COV2.S	2	6.88
3	1683160	6-month durability study	1-dose $5x10^{10}$ vp Ad26.COV2.S	2	4.98
3	1801551	6-month durability study	1-dose $5x10^{10}$ vp Ad26.COV2.S	2	5.50
3	1506260	6-month durability study	1-dose $5x10^{10}$ vp Ad26.COV2.S	4	4.82
3	158284	6-month durability study	1-dose $5x10^{10}$ vp Ad26.COV2.S	4	3.38
3	158298	6-month durability study	1-dose $5x10^{10}$ vp Ad26.COV2.S	4	1.70
3	1680780	6-month durability study	1-dose $5x10^{10}$ vp Ad26.COV2.S	4	5.02
3	1682300	6-month durability study	1-dose $5x10^{10}$ vp Ad26.COV2.S	4	3.93
3	1683160	6-month durability study	1-dose $5x10^{10}$ vp Ad26.COV2.S	4	3.74

Animal Group	Animal_ID	Study	Vaccine	Timepoint	Nose Viral Load (Log10 sgRNA copies/swab)
3	1801551	6-month durability study	1-dose 5x10^{10} vp Ad26.COV2.S	4	5.11
3	1506260	6-month durability study	1-dose 5x10^{10} vp Ad26.COV2.S	7	3.18
3	158284	6-month durability study	1-dose 5x10^{10} vp Ad26.COV2.S	7	1.70
3	158298	6-month durability study	1-dose 5x10^{10} vp Ad26.COV2.S	7	1.70
3	1680780	6-month durability study	1-dose 5x10^{10} vp Ad26.COV2.S	7	4.72
3	1682300	6-month durability study	1-dose 5x10^{10} vp Ad26.COV2.S	7	1.70
3	1683160	6-month durability study	1-dose 5x10^{10} vp Ad26.COV2.S	7	3.55
3	1801551	6-month durability study	1-dose 5x10^{10} vp Ad26.COV2.S	7	4.24
3	1506260	6-month durability study	1-dose 5x10^{10} vp Ad26.COV2.S	10	1.70
3	158284	6-month durability study	1-dose 5x10^{10} vp Ad26.COV2.S	10	1.70
3	158298	6-month durability study	1-dose 5x10^{10} vp Ad26.COV2.S	10	1.70
3	1680780	6-month durability study	1-dose 5x10^{10} vp Ad26.COV2.S	10	1.70
3	1682300	6-month durability study	1-dose 5x10^{10} vp Ad26.COV2.S	10	1.70
3	1683160	6-month durability study	1-dose 5x10^{10} vp Ad26.COV2.S	10	1.70
3	1801551	6-month durability study	1-dose 5x10^{10} vp Ad26.COV2.S	10	1.70
3	1506260	6-month durability study	1-dose 5x10^{10} vp Ad26.COV2.S	14	1.70
3	158284	6-month durability study	1-dose 5x10^{10} vp Ad26.COV2.S	14	1.70
3	158298	6-month durability study	1-dose 5x10^{10} vp Ad26.COV2.S	14	1.70
3	1680780	6-month durability study	1-dose 5x10^{10} vp Ad26.COV2.S	14	1.70
3	1682300	6-month durability study	1-dose 5x10^{10} vp Ad26.COV2.S	14	1.70
3	1683160	6-month durability study	1-dose 5x10^{10} vp Ad26.COV2.S	14	1.70
3	1801551	6-month durability study	1-dose 5x10^{10} vp Ad26.COV2.S	14	1.70

sgRNA: subgenomic RNA; vp: viral particles

Panel d

Animal Group	Animal_ID	Study	Vaccine	Timepoint	Nose Viral Load (Log10 sgRNA copies/swab)
4	158278	6-month durability study	2-dose $5x10^{10}$ vp Ad26.COV2.S (0-4)	-1	1.70
4	158282	6-month durability study	2-dose $5x10^{10}$ vp Ad26.COV2.S (0-4)	-1	1.70
4	1600560	6-month durability study	2-dose $5x10^{10}$ vp Ad26.COV2.S (0-4)	-1	1.70
4	1680120	6-month durability study	2-dose $5x10^{10}$ vp Ad26.COV2.S (0-4)	-1	1.70
4	1683440	6-month durability study	2-dose $5x10^{10}$ vp Ad26.COV2.S (0-4)	-1	1.70
4	1701220	6-month durability study	2-dose $5x10^{10}$ vp Ad26.COV2.S (0-4)	-1	1.70
4	1706171	6-month durability study	2-dose $5x10^{10}$ vp Ad26.COV2.S (0-4)	-1	1.70
4	158278	6-month durability study	2-dose $5x10^{10}$ vp Ad26.COV2.S (0-4)	1	3.75
4	158282	6-month durability study	2-dose $5x10^{10}$ vp Ad26.COV2.S (0-4)	1	4.02
4	1600560	6-month durability study	2-dose $5x10^{10}$ vp Ad26.COV2.S (0-4)	1	4.49
4	1680120	6-month durability study	2-dose $5x10^{10}$ vp Ad26.COV2.S (0-4)	1	3.17
4	1683440	6-month durability study	2-dose $5x10^{10}$ vp Ad26.COV2.S (0-4)	1	3.96
4	1701220	6-month durability study	2-dose $5x10^{10}$ vp Ad26.COV2.S (0-4)	1	4.04
4	1706171	6-month durability study	2-dose $5x10^{10}$ vp Ad26.COV2.S (0-4)	1	1.70
4	158278	6-month durability study	2-dose $5x10^{10}$ vp Ad26.COV2.S (0-4)	2	5.44
4	158282	6-month durability study	2-dose $5x10^{10}$ vp Ad26.COV2.S (0-4)	2	4.13
4	1600560	6-month durability study	2-dose $5x10^{10}$ vp Ad26.COV2.S (0-4)	2	6.66
4	1680120	6-month durability study	2-dose $5x10^{10}$ vp Ad26.COV2.S (0-4)	2	4.96
4	1683440	6-month durability study	2-dose $5x10^{10}$ vp Ad26.COV2.S (0-4)	2	4.74
4	1701220	6-month durability study	2-dose $5x10^{10}$ vp Ad26.COV2.S (0-4)	2	6.42
4	1706171	6-month durability study	2-dose $5x10^{10}$ vp Ad26.COV2.S (0-4)	2	1.70
4	158278	6-month durability study	2-dose $5x10^{10}$ vp Ad26.COV2.S (0-4)	4	4.98
4	158282	6-month durability study	2-dose $5x10^{10}$ vp Ad26.COV2.S (0-4)	4	5.15
4	1600560	6-month durability study	2-dose $5x10^{10}$ vp Ad26.COV2.S (0-4)	4	5.69
4	1680120	6-month durability study	2-dose $5x10^{10}$ vp Ad26.COV2.S (0-4)	4	5.52
4	1683440	6-month durability study	2-dose $5x10^{10}$ vp Ad26.COV2.S (0-4)	4	3.71
4	1701220	6-month durability study	2-dose $5x10^{10}$ vp Ad26.COV2.S (0-4)	4	6.21

Animal Group	Animal_ID	Study	Vaccine	Timepoint	Nose Viral Load (Log10 sgRNA copies/swab)
4	1706171	6-month durability study	2-dose $5x10^{10}$ vp Ad26.COV2.S (0-4)	4	1.70
4	158278	6-month durability study	2-dose $5x10^{10}$ vp Ad26.COV2.S (0-4)	7	2.95
4	158282	6-month durability study	2-dose $5x10^{10}$ vp Ad26.COV2.S (0-4)	7	1.70
4	1600560	6-month durability study	2-dose $5x10^{10}$ vp Ad26.COV2.S (0-4)	7	4.00
4	1680120	6-month durability study	2-dose $5x10^{10}$ vp Ad26.COV2.S (0-4)	7	2.11
4	1683440	6-month durability study	2-dose $5x10^{10}$ vp Ad26.COV2.S (0-4)	7	2.79
4	1701220	6-month durability study	2-dose $5x10^{10}$ vp Ad26.COV2.S (0-4)	7	3.26
4	1706171	6-month durability study	2-dose $5x10^{10}$ vp Ad26.COV2.S (0-4)	7	1.70
4	158278	6-month durability study	2-dose $5x10^{10}$ vp Ad26.COV2.S (0-4)	10	1.70
4	158282	6-month durability study	2-dose $5x10^{10}$ vp Ad26.COV2.S (0-4)	10	1.70
4	1600560	6-month durability study	2-dose $5x10^{10}$ vp Ad26.COV2.S (0-4)	10	1.70
4	1680120	6-month durability study	2-dose $5x10^{10}$ vp Ad26.COV2.S (0-4)	10	1.70
4	1683440	6-month durability study	2-dose $5x10^{10}$ vp Ad26.COV2.S (0-4)	10	2.20
4	1701220	6-month durability study	2-dose $5x10^{10}$ vp Ad26.COV2.S (0-4)	10	1.70
4	1706171	6-month durability study	2-dose $5x10^{10}$ vp Ad26.COV2.S (0-4)	10	1.70
4	158278	6-month durability study	2-dose $5x10^{10}$ vp Ad26.COV2.S (0-4)	14	1.70
4	158282	6-month durability study	2-dose $5x10^{10}$ vp Ad26.COV2.S (0-4)	14	1.70
4	1600560	6-month durability study	2-dose $5x10^{10}$ vp Ad26.COV2.S (0-4)	14	1.70
4	1680120	6-month durability study	2-dose $5x10^{10}$ vp Ad26.COV2.S (0-4)	14	1.70
4	1683440	6-month durability study	2-dose $5x10^{10}$ vp Ad26.COV2.S (0-4)	14	1.70
4	1701220	6-month durability study	2-dose $5x10^{10}$ vp Ad26.COV2.S (0-4)	14	1.70
4	1706171	6-month durability study	2-dose $5x10^{10}$ vp Ad26.COV2.S (0-4)	14	1.70

sgRNA: subgenomic RNA; vp: viral particles

Panel e

Animal Group	Animal_ID	Study	Vaccine	Timepoint	Nose Viral Load (Log10 sgRNA copies/swab)
5	158280	6-month durability study	2-dose $5x10^{10}$ vp Ad26.COV2.S (0-8)	-1	1.70
5	1606520	6-month durability study	2-dose $5x10^{10}$ vp Ad26.COV2.S (0-8)	-1	1.70
5	1680260	6-month durability study	2-dose $5x10^{10}$ vp Ad26.COV2.S (0-8)	-1	1.70
5	1682671	6-month durability study	2-dose $5x10^{10}$ vp Ad26.COV2.S (0-8)	-1	1.70
5	1683140	6-month durability study	2-dose $5x10^{10}$ vp Ad26.COV2.S (0-8)	-1	1.70
5	1683520	6-month durability study	2-dose $5x10^{10}$ vp Ad26.COV2.S (0-8)	-1	1.70
5	1683640	6-month durability study	2-dose $5x10^{10}$ vp Ad26.COV2.S (0-8)	-1	1.70
5	158280	6-month durability study	2-dose $5x10^{10}$ vp Ad26.COV2.S (0-8)	1	5.74
5	1606520	6-month durability study	2-dose $5x10^{10}$ vp Ad26.COV2.S (0-8)	1	1.70
5	1680260	6-month durability study	2-dose $5x10^{10}$ vp Ad26.COV2.S (0-8)	1	3.78
5	1682671	6-month durability study	2-dose $5x10^{10}$ vp Ad26.COV2.S (0-8)	1	1.70
5	1683140	6-month durability study	2-dose $5x10^{10}$ vp Ad26.COV2.S (0-8)	1	1.70
5	1683520	6-month durability study	2-dose $5x10^{10}$ vp Ad26.COV2.S (0-8)	1	2.73
5	1683640	6-month durability study	2-dose $5x10^{10}$ vp Ad26.COV2.S (0-8)	1	1.70
5	158280	6-month durability study	2-dose $5x10^{10}$ vp Ad26.COV2.S (0-8)	2	5.72
5	1606520	6-month durability study	2-dose $5x10^{10}$ vp Ad26.COV2.S (0-8)	2	3.22
5	1680260	6-month durability study	2-dose $5x10^{10}$ vp Ad26.COV2.S (0-8)	2	3.67
5	1682671	6-month durability study	2-dose $5x10^{10}$ vp Ad26.COV2.S (0-8)	2	1.70
5	1683140	6-month durability study	2-dose $5x10^{10}$ vp Ad26.COV2.S (0-8)	2	4.15
5	1683520	6-month durability study	2-dose $5x10^{10}$ vp Ad26.COV2.S (0-8)	2	4.62
5	1683640	6-month durability study	2-dose $5x10^{10}$ vp Ad26.COV2.S (0-8)	2	3.49
5	158280	6-month durability study	2-dose $5x10^{10}$ vp Ad26.COV2.S (0-8)	4	4.44
5	1606520	6-month durability study	2-dose $5x10^{10}$ vp Ad26.COV2.S (0-8)	4	1.70
5	1680260	6-month durability study	2-dose $5x10^{10}$ vp Ad26.COV2.S (0-8)	4	2.80
5	1682671	6-month durability study	2-dose $5x10^{10}$ vp Ad26.COV2.S (0-8)	4	1.70
5	1683140	6-month durability study	2-dose $5x10^{10}$ vp Ad26.COV2.S (0-8)	4	4.16
5	1683520	6-month durability study	2-dose $5x10^{10}$ vp Ad26.COV2.S (0-8)	4	5.75

Animal Group	Animal_ID	Study	Vaccine	Timepoint	Nose Viral Load (Log10 sgRNA copies/swab)
5	1683640	6-month durability study	2-dose 5x10^{10} vp Ad26.COV2.S (0-8)	4	1.70
5	158280	6-month durability study	2-dose 5x10^{10} vp Ad26.COV2.S (0-8)	7	3.42
5	1606520	6-month durability study	2-dose 5x10^{10} vp Ad26.COV2.S (0-8)	7	1.70
5	1680260	6-month durability study	2-dose 5x10^{10} vp Ad26.COV2.S (0-8)	7	1.70
5	1682671	6-month durability study	2-dose 5x10^{10} vp Ad26.COV2.S (0-8)	7	1.70
5	1683140	6-month durability study	2-dose 5x10^{10} vp Ad26.COV2.S (0-8)	7	1.70
5	1683520	6-month durability study	2-dose 5x10^{10} vp Ad26.COV2.S (0-8)	7	2.91
5	1683640	6-month durability study	2-dose 5x10^{10} vp Ad26.COV2.S (0-8)	7	5.20
5	158280	6-month durability study	2-dose 5x10^{10} vp Ad26.COV2.S (0-8)	10	1.70
5	1606520	6-month durability study	2-dose 5x10^{10} vp Ad26.COV2.S (0-8)	10	1.70
5	1680260	6-month durability study	2-dose 5x10^{10} vp Ad26.COV2.S (0-8)	10	1.70
5	1682671	6-month durability study	2-dose 5x10^{10} vp Ad26.COV2.S (0-8)	10	1.70
5	1683140	6-month durability study	2-dose 5x10^{10} vp Ad26.COV2.S (0-8)	10	1.70
5	1683520	6-month durability study	2-dose 5x10^{10} vp Ad26.COV2.S (0-8)	10	1.70
5	1683640	6-month durability study	2-dose 5x10^{10} vp Ad26.COV2.S (0-8)	10	2.90
5	158280	6-month durability study	2-dose 5x10^{10} vp Ad26.COV2.S (0-8)	14	1.70
5	1606520	6-month durability study	2-dose 5x10^{10} vp Ad26.COV2.S (0-8)	14	1.70
5	1680260	6-month durability study	2-dose 5x10^{10} vp Ad26.COV2.S (0-8)	14	1.70
5	1682671	6-month durability study	2-dose 5x10^{10} vp Ad26.COV2.S (0-8)	14	1.70
5	1683140	6-month durability study	2-dose 5x10^{10} vp Ad26.COV2.S (0-8)	14	1.70
5	1683520	6-month durability study	2-dose 5x10^{10} vp Ad26.COV2.S (0-8)	14	1.70
5	1683640	6-month durability study	2-dose 5x10^{10} vp Ad26.COV2.S (0-8)	14	1.70

sgRNA: subgenomic RNA; vp: viral particles

Panel f

Animal Group	Animal_ID	Study	Vaccine	No. sampling days with viral load>LOD (Nose)
1	158274	6-month durability study	Sham	4
1	158411	6-month durability study	Sham	4
1	158422	6-month durability study	Sham	4
1	1606400	6-month durability study	Sham	6
1	1680940	6-month durability study	Sham	6
1	1682980	6-month durability study	Sham	4
1	1683040	6-month durability study	Sham	4
2	158270	6-month durability study	1-dose $1x10^{11}$ vp Ad26.COV2.S	2
2	158272	6-month durability study	1-dose $1x10^{11}$ vp Ad26.COV2.S	0
2	158300	6-month durability study	1-dose $1x10^{11}$ vp Ad26.COV2.S	3
2	1606460	6-month durability study	1-dose $1x10^{11}$ vp Ad26.COV2.S	3
2	1681960	6-month durability study	1-dose $1x10^{11}$ vp Ad26.COV2.S	4
2	1683000	6-month durability study	1-dose $1x10^{11}$ vp Ad26.COV2.S	3
2	1683360	6-month durability study	1-dose $1x10^{11}$ vp Ad26.COV2.S	4
3	1506260	6-month durability study	1-dose $5x10^{10}$ vp Ad26.COV2.S	4
3	158284	6-month durability study	1-dose $5x10^{10}$ vp Ad26.COV2.S	3
3	158298	6-month durability study	1-dose $5x10^{10}$ vp Ad26.COV2.S	0
3	1680780	6-month durability study	1-dose $5x10^{10}$ vp Ad26.COV2.S	4
3	1682300	6-month durability study	1-dose $5x10^{10}$ vp Ad26.COV2.S	3
3	1683160	6-month durability study	1-dose $5x10^{10}$ vp Ad26.COV2.S	4
3	1801551	6-month durability study	1-dose $5x10^{10}$ vp Ad26.COV2.S	4
4	158278	6-month durability study	2-dose $5x10^{10}$ vp Ad26.COV2.S (0-4)	4
4	158282	6-month durability study	2-dose $5x10^{10}$ vp Ad26.COV2.S (0-4)	3
4	1600560	6-month durability study	2-dose $5x10^{10}$ vp Ad26.COV2.S (0-4)	4
4	1680120	6-month durability study	2-dose $5x10^{10}$ vp Ad26.COV2.S (0-4)	4
4	1683440	6-month durability study	2-dose $5x10^{10}$ vp Ad26.COV2.S (0-4)	5
4	1701220	6-month durability study	2-dose $5x10^{10}$ vp Ad26.COV2.S (0-4)	4
4	1706171	6-month durability study	2-dose $5x10^{10}$ vp Ad26.COV2.S (0-4)	0
5	158280	6-month durability study	2-dose $5x10^{10}$ vp Ad26.COV2.S (0-8)	4
5	1606520	6-month durability study	2-dose $5x10^{10}$ vp Ad26.COV2.S (0-8)	1

**Animal Group**	**Animal_ID**	**Study**	**Vaccine**	**No. sampling days with viral load>LOD (Nose)**
5	1680260	6-month durability study	2-dose $5x10^{10}$ vp Ad26.COV2.S (0-8)	3
5	1682671	6-month durability study	2-dose $5x10^{10}$ vp Ad26.COV2.S (0-8)	0
5	1683140	6-month durability study	2-dose $5x10^{10}$ vp Ad26.COV2.S (0-8)	2
5	1683520	6-month durability study	2-dose $5x10^{10}$ vp Ad26.COV2.S (0-8)	4
5	1683640	6-month durability study	2-dose $5x10^{10}$ vp Ad26.COV2.S (0-8)	3

LOD: limit of detection; vp: viral particles

Supplementary Table 11: Source data file for Supplementary Figure 5

		Lung One-dose	Lung Two-dose	Lung Combined	Nose One-dose	Nose Two-dose	Nose Combined
**S-ELISA**	Observed (Mean)	92.9%	85.7%	89.3%	14.3%	14.3%	14.3%
	[95% CI]	[58.0-99.2%]	[53.5-96.9%]	[70.4-96.7%]	[3.1-46.5%]	[3.1-46.5%]	[5.2-33.5%]
	Prediction Analysis A[1] (Mean)	72.6%	74.4%	73.5%	45.5%	49.3%	47.4%
	[95% CI]	[57.0-86.8%]	[60.8-87.4%]	[59.9-86.9%]	[27.6-62.2%]	[32.2-64.8%]	[30.6-62.7%]
	Prediction Analysis B[2] (Mean)	29.0%	30.0%	30.0%	0.0%	0.0%	0.0%
	[95% CI]	[0.0-83.0%]	[0.0-93.0%]	[0.0-86.0%]	[0.0-7.0%]	[0.0-7.0%]	[0.0-7.0%]
**psVNA**	Observed (Mean)	92.9%	85.7%	89.3%	14.3%	14.3%	14.3%
	[95% CI]	[58.0-99.2%]	[53.5-96.9%]	[70.4-96.7%]	[3.1-46.5%]	[3.1-46.5%]	[5.2-33.5%]
	Prediction Analysis A[1] (Mean)	86.7%	88.2%	87.5%	75.7%	79.8%	77.7%
	[95% CI]	[76.0-95.0%]	[79.3-95.9%]	[78.6-95.2%]	[58.3-90.9%]	[64.7-93.0%]	[63.5-90.2%]
	Prediction Analysis B[2] (Mean)	81.0%	90.0%	86.0%	53.0%	53.0%	53.0%
	[95% CI]	[57.0-93.0%]	[57.0-100.0%]	[57.0-96.0%]	[36.0-86.0%]	[14.0-86.0%]	[25.0-82.0%]

[1]logistic models; [2]sigmoid Emax models
S-ELISA: spike protein enzyme-linked immunosorbent assay ; psVNA: pseudotyped virus neutralization assay
