# Supplementary material for: SARS-CoV-2 binding and neutralizing antibody levels after Ad26.COV2.S vaccination predict durable protection in rhesus macaques
Source: Nat Commun. 2021 Oct 7;12:5877. doi: 10.1038/s41467-021-26117-x (PMC8497464; doi:10.1038/s41467-021-26117-x)
Supplement: Supplementary file 2 — Reporting Summary [file 41467_2021_26117_MOESM2_ESM.pdf]

nature portfolio

Corresponding author(s): Roland Zahn
Last updated by author(s): August 27th, 2021



# Reporting Summary

Nature Portfolio wishes to improve the reproducibility of the work that we publish. This form provides structure for consistency and transparency in reporting. For further information on Nature Portfolio policies, see our Editorial Policies and the Editorial Policy Checklist.

## Statistics

For all statistical analyses, confirm that the following items are present in the figure legend, table legend, main text, or Methods section.

| n/a | Confirmed | |
|---|---|---|
| ☐ | ☒ | The exact sample size (*n*) for each experimental group/condition, given as a discrete number and unit of measurement |
| ☐ | ☒ | A statement on whether measurements were taken from distinct samples or whether the same sample was measured repeatedly |
| ☐ | ☒ | The statistical test(s) used AND whether they are one- or two-sided<br>*Only common tests should be described solely by name; describe more complex techniques in the Methods section.* |
| ☐ | ☒ | A description of all covariates tested |
| ☐ | ☒ | A description of any assumptions or corrections, such as tests of normality and adjustment for multiple comparisons |
| ☐ | ☒ | A full description of the statistical parameters including central tendency (e.g. means) or other basic estimates (e.g. regression coefficient) AND variation (e.g. standard deviation) or associated estimates of uncertainty (e.g. confidence intervals) |
| ☐ | ☒ | For null hypothesis testing, the test statistic (e.g. *F*, *t*, *r*) with confidence intervals, effect sizes, degrees of freedom and *P* value noted<br>*Give P values as exact values whenever suitable.* |
| ☒ | ☐ | For Bayesian analysis, information on the choice of priors and Markov chain Monte Carlo settings |
| ☒ | ☐ | For hierarchical and complex designs, identification of the appropriate level for tests and full reporting of outcomes |
| ☒ | ☐ | Estimates of effect sizes (e.g. Cohen's *d*, Pearson's *r*), indicating how they were calculated |

*Our web collection on statistics for biologists contains articles on many of the points above.*

## Software and code

Policy information about availability of computer code

| | |
|---|---|
| Data collection | Simulations reported here were conducted using R version 3.6.0 on an x86_64-redhat-linux-gnu (64-bit) platform running under a Red Hat Enterprise Linux Server 7.4 (Maipo) using Rstudio Version 1.1.453. |
| Data analysis | Simulations reported here were conducted using R version 3.6.0 on an x86_64-redhat-linux-gnu (64-bit) platform running under a Red Hat Enterprise Linux Server 7.4 (Maipo) using Rstudio Version 1.1.453. The viral kinetic analysis was performed using NONMEM 7.4 (ICON plc, Hanover, MD, USA) in a Windows 7 operating system. Perl-speaks-NONMEM (PsN, version 4.2.0, [http://psn.sourceforge.net/docs.php]).<br>The estimated mean population probability of protection for the challenged macaques, together with a 95%CI based on their viral load measurements after challenge, are analyzed using a logistic regression model with overall intercept only using the glm functionality (lme4 package R). |

For manuscripts utilizing custom algorithms or software that are central to the research but not yet described in published literature, software must be made available to editors and reviewers. We strongly encourage code deposition in a community repository (e.g. GitHub). See the Nature Portfolio guidelines for submitting code & software for further information.

## Data

Policy information about availability of data

All manuscripts must include a data availability statement. This statement should provide the following information, where applicable:

- Accession codes, unique identifiers, or web links for publicly available datasets
- A description of any restrictions on data availability
- For clinical datasets or third party data, please ensure that the statement adheres to our policy

The data sharing policy of Janssen, Pharmaceutical Companies of Johnson & Johnson, is available at https://www.janssen.com/transparency. All data presented in

the manuscript are available in Supplementary Tables 4, 5, 6, 7, 8, 9, 10 and 11.

# Field-specific reporting

Please select the one below that is the best fit for your research. If you are not sure, read the appropriate sections before making your selection.

- [x] Life sciences
- [ ] Behavioural & social sciences
- [ ] Ecological, evolutionary & environmental sciences

For a reference copy of the document with all sections, see nature.com/documents/nr-reporting-summary-flat.pdf

# Life sciences study design

All studies must disclose on these points even when the disclosure is negative.

| | |
|---|---|
| Sample size | Sample size was not pre-determined. Data were obtained from 3 independent NHP studies, and data from a total of 81 NHP was included in modeling. Predictions were compared against data from a single study, testing durability of protection 6 months after vaccination with Ad26.COV2.S, in a total of 28 NHP. |
| Data exclusions | No data were excluded from the analysis |
| Replication | Modeling was based on 3 independent NHP studies. Modeling outcomes were compared against data from a single study. |
| Randomization | Group allocations of rhesus macaques was done based on block randomization, harmonized for bodyweight and sex |
| Blinding | Investigators were blinded to group allocation at the time of data collection. |

# Reporting for specific materials, systems and methods

We require information from authors about some types of materials, experimental systems and methods used in many studies. Here, indicate whether each material, system or method listed is relevant to your study. If you are not sure if a list item applies to your research, read the appropriate section before selecting a response.

## Materials & experimental systems

| n/a | Involved in the study | |
|---|---|---|
| x | | Antibodies |
| | x | Eukaryotic cell lines |
| x | | Palaeontology and archaeology |
| | x | Animals and other organisms |
| x | | Human research participants |
| x | | Clinical data |
| x | | Dual use research of concern |

## Methods

| n/a | Involved in the study | |
|---|---|---|
| x | | ChIP-seq |
| x | | Flow cytometry |
| x | | MRI-based neuroimaging |

## Eukaryotic cell lines

Policy information about cell lines

| | |
|---|---|
| Cell line source(s) | HEK293T-hACE2 cells were obtained from BEI Resource |
| Authentication | Morphology, growth properties, protein expression, species specific PCR |
| Mycoplasma contamination | Tested negative |
| Commonly misidentified lines (See ICLAC register) | No commonly misidentified lines were used |

## Animals and other organisms

Policy information about studies involving animals; ARRIVE guidelines recommended for reporting animal research

| | |
|---|---|
| Laboratory animals | Male and female adult (>3 years of age) rhesus macaques were obtained from Kunmings Biomed international Ltd, China. Prior to transfer from test facility colony, all animals were subjected to a health assessment and tested at least once for tuberculosis by intradermal injection of tuberculin. An anthelmintic treatment was administered to each animal by subcutaneous injection. The evaluations were performed in accordance with the standard operating procedures by technical staff. |
| Wild animals | No wild animals were used. |

| | |
|---|---|
| Field-collected samples | No field-collected samples were used. |
| Ethics oversight | Animal experiments were approval by the Institutional Animal Care and Use Committees (IACUC) of Charles River (Laval, Canada) and Bioqual (Rockville, MD, US). Animal facilities were accredited by the Association for Assessment and Accreditation of Laboratory Animal Care. Experiments were conducted in compliance with national regulations. |

Note that full information on the approval of the study protocol must also be provided in the manuscript.
